# Selective Electrosynthetic Hydrocarboxylation of α,β‐Unsaturated Esters with Carbon Dioxide**

**DOI:** 10.1002/anie.202105490

**Published:** 2021-09-06

**Authors:** Ahmed M. Sheta, Anas Alkayal, Mohammad A. Mashaly, Samy B. Said, Saad S. Elmorsy, Andrei V. Malkov, Benjamin R. Buckley

**Affiliations:** ^1^ Department of Chemistry Loughborough University Loughborough Leicestershire LE11 3TU UK; ^2^ Department of Chemistry Damietta University Damietta El-Gadeeda City, Kafr Saad, Damietta Governorate 34511 Egypt; ^3^ Department of Chemistry Mansoura University 25 El Gomhouria St Dakahlia Governorate 35516 Egypt

**Keywords:** acrylate, carbon dioxide, electron transfer, electrochemistry, reduction

## Abstract

The carboxylation of low‐value commodity chemicals to provide higher‐value carboxylic acids is of significant interest. Recently alternative routes to the traditional hydroformylation processes that used potentially toxic carbon monoxide and a transition metal catalyst have appeared. A significant challenge has been the selectivity observed for olefin carboxylation. Photochemical methods have shown a viable route towards the hydrocarboxylation of α,β‐unsaturated alkenes but rely on the use of an excess reducing or amine reagent. Herein we report our investigations of an electrochemical approach that is able to hydrocarboxylate α,β‐unsaturated alkenes with excellent regioselectivity and the ability to carboxylate hindered substrates to afford α‐quaternary center carboxylic acids. The reported process requires no chromatography and the products are purified by simple crystallization from the reaction mixture after work‐up.

## Graphical abstract

Electrosynthetic hydrocarboxylation of α,β‐unsaturated olefins with excellent regioselectivity is reported. The method requires no sacrificial electrode and is thus amenable to a flow configuration. The products are purified by simple crystallization. The synthesis of a precursor to ethosuximide, which contains an all‐carbon quaternary center, illustrates the potential of the process. Finally, a robustness study has benchmarked the process for future users.

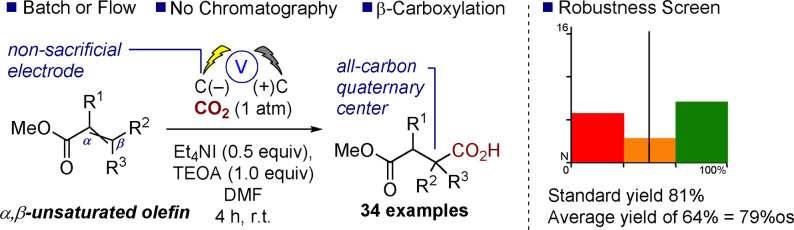

## Introduction

**Scheme 1:**
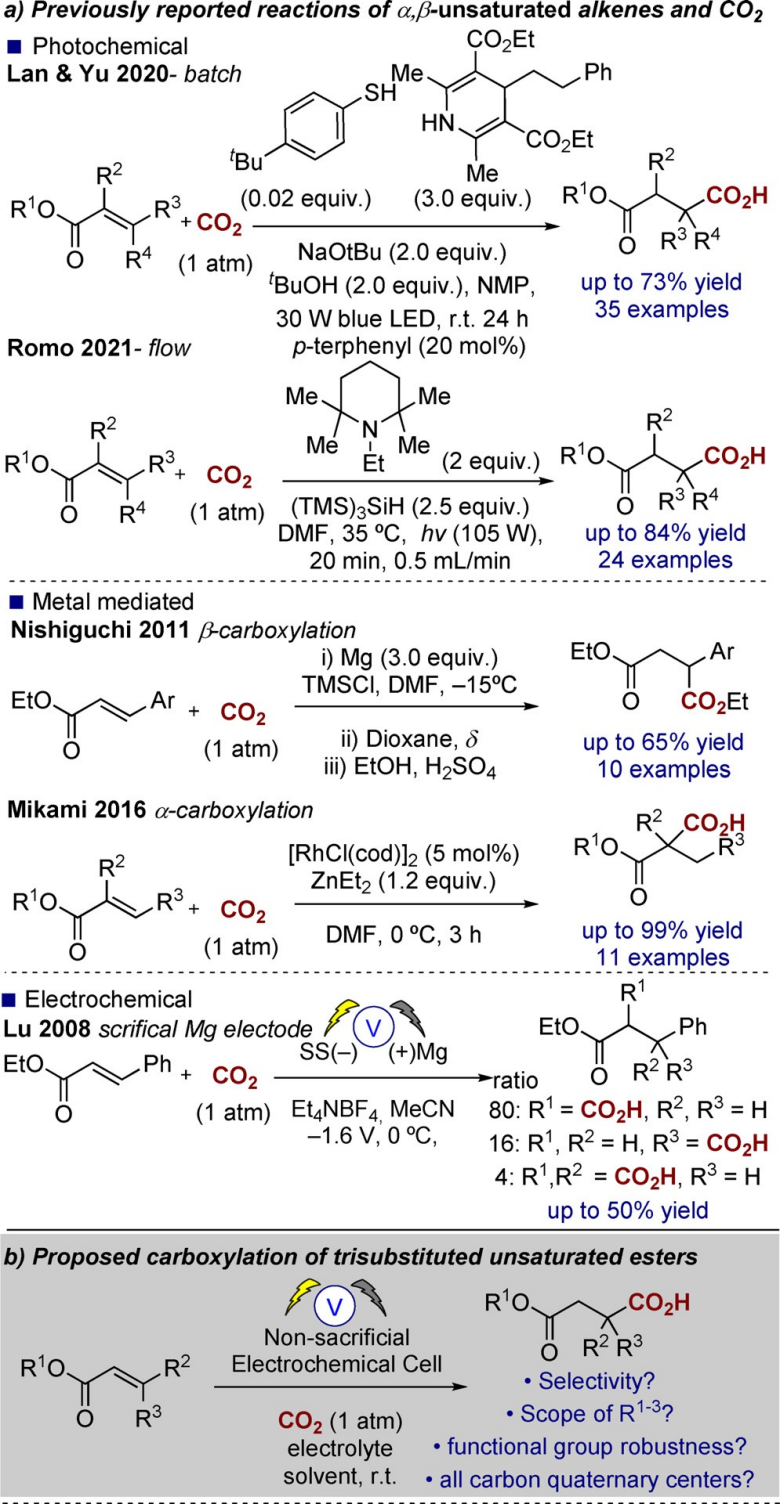
Current/proposed approach to α,β‐unsaturated ester carboxylation.

Direct selective carboxylation of α,β‐unsaturated esters with carbon dioxide remains a challenging task despite a number of successful attempts via either metal‐mediated, electrochemical, or photochemical means (Scheme 1 a).[^1^, ^2^, ^3^] Recently Lan and Yu introduced a photochemical approach to a range of α,β‐unsaturated esters providing good yields of the corresponding β‐hydrocarboxylated products, using stoichiometric or catalytic aromatic thiols.^[3a]^ Romo, utilizing the conditions originally reported by Jamison for the hydrocarboxylation of styrenes,^[4]^ has shown that the photochemical hydrocarboxylation of α,β‐unsaturated esters can also be carried out under flow conditions and some of the products containing all‐carbon quaternary centers can be accessed in good to excellent yield. Additionally, they showed that these products are versatile building blocks for lactonization.^[3b]^ A magnesium‐mediated (3 equivalents) approach has provided β‐carboxylated products from aryl cinnamate esters through a sequence of dicarboxylation and mono‐decarboxylation whereas Makami has disclosed a rhodium‐catalyzed approach to α‐carboxylation requiring the use of diethylzinc (1.2 equivalents) to successfully provide the desired products.[^1a^, ^1b^]

At present, there are no satisfactory reports of utilizing electrosynthesis in the carboxylation of non‐aryl α,β‐unsaturated esters^[2a]^ and those reported with aryl substituents, such as that by Lu with *trans*‐cinnamate esters, provide a mixture of mono‐ and dicarboxylated products and rely on a sacrificial magnesium anode (or in the case of Wang and Zhang an aluminum sacrificial anode).[^2b^, ^2c^]

The formation of all‐carbon quaternary centers bearing a potentially labile carboxylic acid group represents a significant challenge in organic synthesis.^[5]^ Interest in this area stems from the unique structural features of quaternary centers for applications in synthesis, which is further enabled by the resultant synthetic flexibility of the carboxylate group. Although many excellent methods have been reported that allow for the carboxylation of olefins these are limited to styrenes with little or no substitution about the double bond.^[6]^ Recently, research efforts in this laboratory have explored the utility of electrosynthesis for carbon dioxide incorporation including aryl alkenes and dienes,[^7a^, ^7b^] with a limited range of trisubstituted alkenes affording all‐carbon quaternary centers.^[7a]^ Pivotal to the success of this work was the development of a cell that comprised a non‐sacrificial electrode.

In order to directly form all‐carbon quaternary centers bearing a carboxylic acid group, one would typically rely on hydroformylation followed by oxidation to access these types of compounds, thus requiring precious metal catalysts (typically Rh) at high pressures of CO (>20 atm) and high temperatures.^[8]^ Often these conditions result in migration of the double bond thus giving rise to carbonylation at a different site to the original location of the double bond.^[9]^ To address this challenge, we envisioned applying our recently reported hydrocarboxylation approach to provide direct access to the desired functionalized quaternary center,^[7a]^ avoiding migration of the double bond and thus expanding the methods by which to access these highly desirable building blocks.

With our operationally simpler methodology that was previously applied to aryl alkenes, we wished to understand the scope and limitations of this electrochemical approach and crucially determine the robustness of our methodology so that it could be future benchmarked. To this end, we herein report the first highly selective electrochemical hydrocarboxylation of α,β‐unsaturated esters that requires no column chromatography, with particular focus on the development of all‐carbon quaternary centers.

## Results and Discussion

Our approach was to gain an understanding of the scope of the substituents attached to the alkene. We, therefore, utilized our previously reported conditions for aryl alkene hydrocarboxylation and screened a range of α,β‐unsaturated alkenes (**1 a**–**p** Table 1).^[7a]^ Replacement of triethanolamine (TEOA) with water as a proton source did not affect regioselectivity, however resulted in slightly lower yields of **1 a** (60 % vs. 76 %). Although the use of H_2_O is attractive, in this instance we chose to utilize the higher‐yielding TEOA conditions, one possible reason for the higher yield in the case of TEOA is its ability to solubilize CO_2_.^[10]^ Under our standardized conditions, we noticed that a number of alkenes rapidly underwent reduction to the corresponding alkane (**1 b**,**m**–**o**) or did not react (**1 c,d**). In the case of **1 d**, this lack of reactivity could be due to the reduced electrophilicity of cyclic unsaturated lactones due to the fixed (*Z*)‐geometry of the *s‐trans* configured π‐system when compared to **1 a**.^[11]^ In addition, we also attempted the hydrocarboxylation of dimethyl vinylphosphonate, *N*,*N*‐dimethylacrylamide, vinyl acetate, *trans*‐1‐phenyl‐2‐buten‐1‐one, vinyl benzoate, all of which were reduced to the corresponding alkanes. For phenyl vinyl sulfone we did observe the carboxylate by GC–MS but were unable to isolate the product after work‐up. We wondered whether this could be due to the reduction potential of the alkene, however, at this point there does not seem to be a logical correlation between reduction potential and reduction/carboxylation, for example, **2 a** *E*_1/2_=−2.10 V and **2 c** *E*_1/2_=−2.14 V vs. SCE in MeCN.^[2a]^ A reduction in the voltage applied to the system did not change the outcome of the reaction when dropped to 5 V, below this level we saw little to no reaction of the alkenes. It is noteworthy that α,β‐unsaturated esters such as **1 a** worked particularly well with excellent regioselectivity for carboxylation at the β‐position and required no column chromatography for purification, they could simply be crystallized after isolation from the reaction mixture.

**Table 1:**
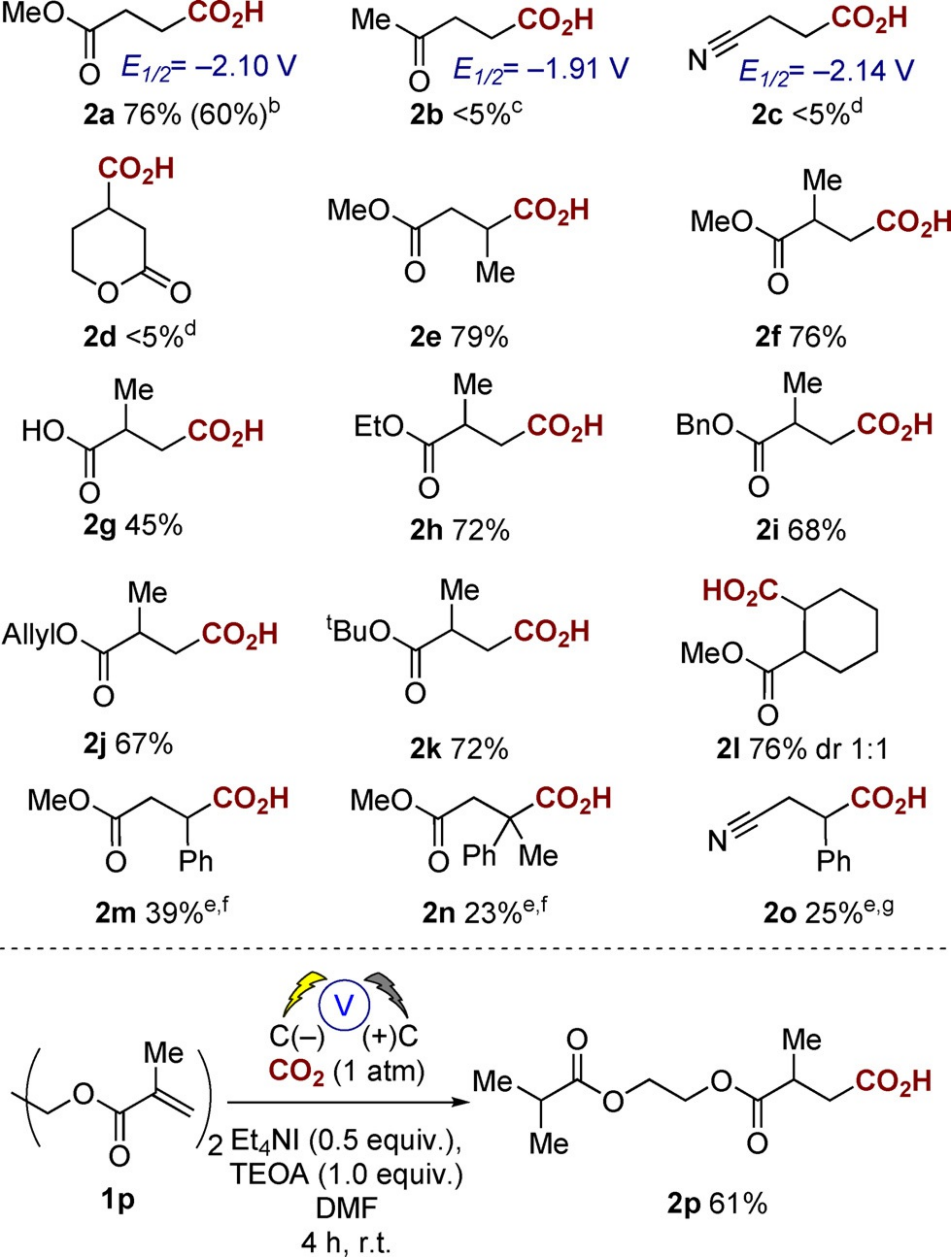
Screening of α,β‐unsaturated alkenes.^[a]^ 
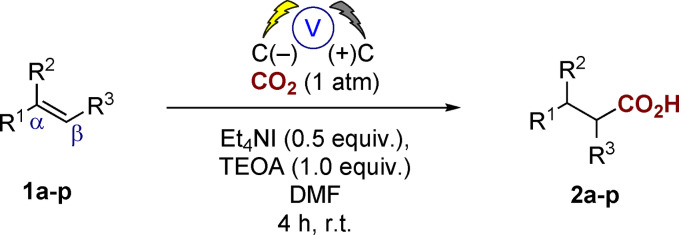

[a] General conditions: CO_2_ (1 atm), carbon anode and cathode, Et_4_NI (0.5 equiv), TEOA (1.0 equiv), DMF, single compartment cell, 10 V (60–100 mA), 4 h rt. [b] Yield in parenthesis refers to the corresponding reaction using H_2_O instead of TEOA as a proton source. [c] Major product identified by GC–MS was butan‐2‐one. [d] Major product identified by GC–MS was unreacted starting material. [e] Competing product of these reactions was the reduced compound by GC–MS analysis. [f] Purified by column chromatography. [g] Unable to isolate pure, **2 o** identified by GC–MS analysis.

We then screened a range of substitution patterns and substituents on the ester (**1 f**–**k**) to evaluate the scope of the approach (Table 1). Acids were isolated by crystallization and a range of common esters were tolerated under the reaction conditions (**1 f**–**k**). In addition, the reaction also proceeded with the acrylic acid **1 g** as the starting material, the yield was somewhat lower than for the corresponding esters (e.g. **2 f**) but we believe this is due to the increased water solubility of the diacid product.^[12]^ Substitution at either or both the α‐ or β‐positions also provided good yields of the corresponding products (**2 e,f** or **2 l**), and at no point did we observe carboxylation at the α‐position or dicarboxylation at both the α,β‐positions (monitored by GC–MS and ^1^H NMR spectroscopy). Carboxylation of the conjugated aromatic alkenes **1 m,n** and the nitrile containing **1 o** resulted in exclusive carboxylation at the β‐carbon albeit in somewhat lower yield due to competing reduction of the double bond to the alkane (observed by GC–MS analysis). Interestingly the symmetrical diester **1 p** resulted in mono‐carboxylation with concomitant reduction of the second double bond as the major product, the expected symmetrical carboxylated product was not observed.^[13]^

Considering the synthetic usefulness of this carboxylation approach we were interested in the application of this to tri‐ or tetra‐substituted alkenes which upon carboxylation would yield an all‐carbon quaternary center at the β‐position. Initial reaction with the β,β‐dimethyl substrate **1 q** gave an excellent yield of the mono‐carboxylated product **2 q** (81 %) as a single regioisomer. Pleasingly we were able to apply these conditions to a range of unfunctionalized β,β‐disubstituted cyclic esters (**1 r**–**w**) and to the amino‐ and oxygen‐containing heterocyclic systems with varying ring sizes (**1 y**–**ae**). In each case good yields of the corresponding hydrocarboxylated all‐carbon quaternary center products were observed, again requiring no chromatography during isolation. Interestingly we observed a mixture of products from the reaction of **1 ac**, with ring expansion also taking place to afford **2 ac′**, initially we thought this may arise through a similar ring expansion of oxetanes reported by us utilizing a magnesium sacrificial electrode and Bu_4_NI,^[14]^ however on replacement of the Et_4_NI with LiBF_4_ we still observed ring opening (although in a 1:1 ratio). We did not observe any ring expansion for the corresponding **1 ab** substrate, perhaps indicating that steric hindrance around the oxetane prevents this from occurring. The sulfur‐containing substrate **1 af** was, however, problematic: we could observe carboxylation by GC–MS analysis but were unable to isolate the final product. This was also true when we attempted carboxylation of the corresponding sulfone substrate. A highlight of the approach is exemplified in the highly diastereoselective hydrocarboxylation of the alkene **1 x** derived from estrone. The sterically crowded alkene afforded the all‐carbon quaternary center on the 5‐membered ring of **2 x** in good yield and >99:1 d.r.

**Scheme 2:**
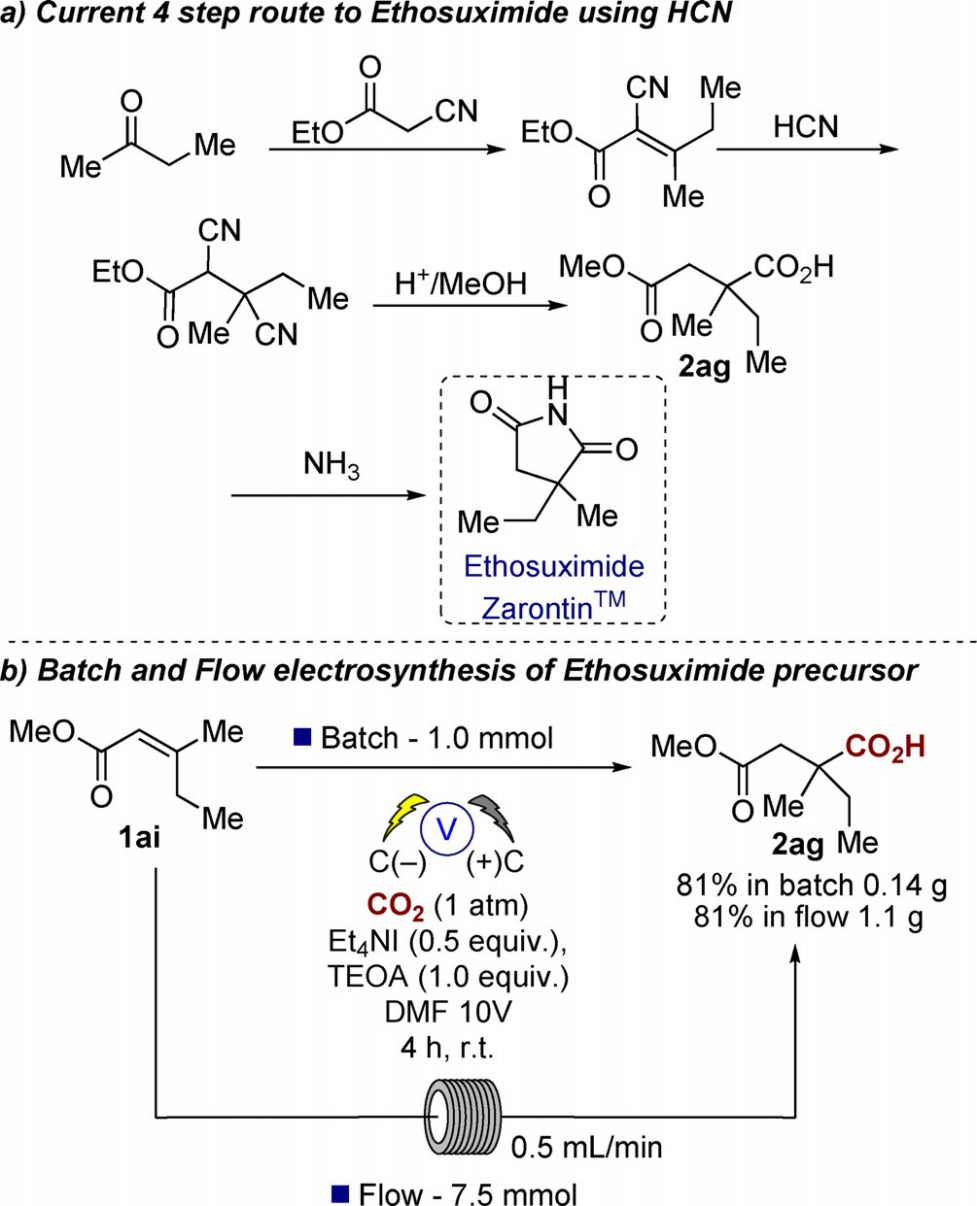
Application of the hydrocarboxylation process to the flow synthesis of anti‐epilepsy and absence seizure drug ethosuximide precursor **2 ag**.

We subsequently utilized our approach to successfully install a β‐all‐carbon quaternary center to prepare compound **2 ag** in 81 % yield (Scheme 2). **2 ag** is in fact a precursor to the anti‐epilepsy and absence seizure drug ethosuximide (Zarontin^TM^) which is manufactured in 4 steps (Scheme 2 a).^[15]^ Our approach removes the requirement for the use of toxic hydrogen cyanide through the direct carboxylation of the α,β‐unsaturated ester **1 ag** (Scheme 2 b).

We then explored the possibility of converting our process from a commercial batch reactor to a commercial flow reactor and our unoptimized conditions utilizing carbon–carbon sheet electrodes, Et_4_NI (0.5 equiv), TEOA (1.0 equiv), DMF resulted in an 81 % yield of the desired product after 4 hours at a 7.5 mmol scale of **1 ag**, delivering the ethosuximide precursor **2 ag** on a gram scale, thus illustrating the simplicity of our approach and the requirement for no bespoke equipment.

**Scheme 3:**
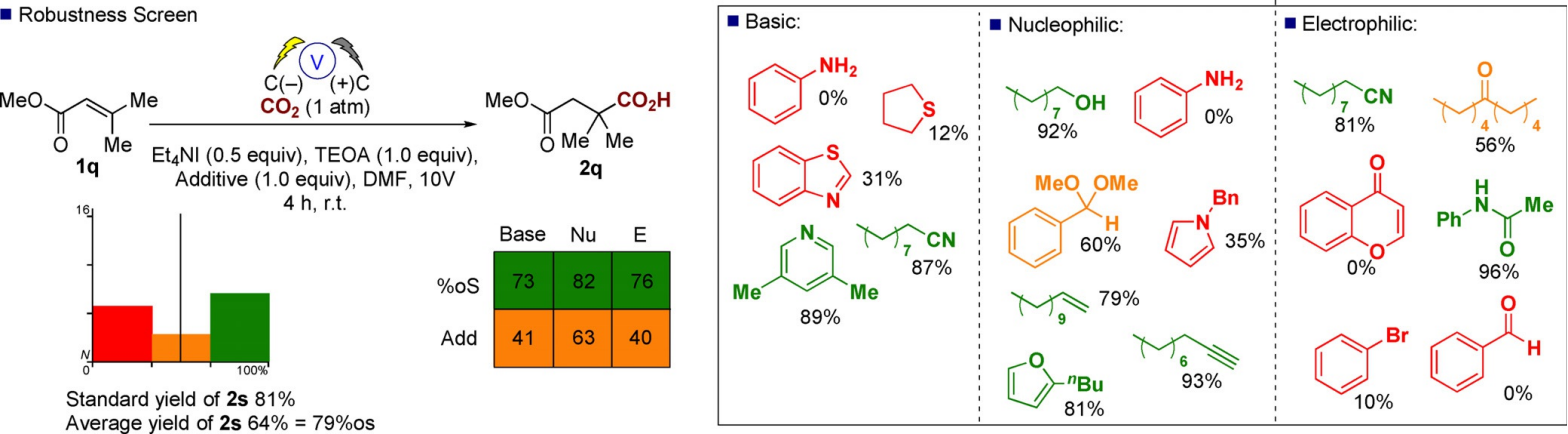
Application of Glorius’ robustness screen (percentage yields shown next to the additives refer to remaining additive quantified by GC–MS analysis at the end of the reaction).

**Scheme 4:**
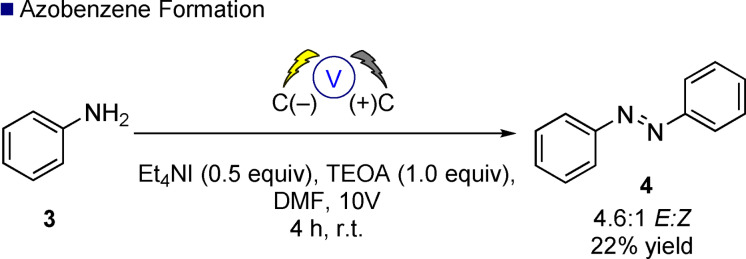
Blank reaction containing only the aniline **3** additive from the robustness screen.

Next, we investigated the scope and limitations of this electrochemical approach to alkene functionalization and enable future users to be able to benchmark against our process. Glorius has developed a useful approach to examine functional group tolerance through utilization of a protocol to rapidly determine reaction condition compatibility.^[16]^ A range of photochemical processes have been examined, identifying photobleaching as an issue, but to the best of our knowledge, this approach has not been applied to examine the functional group robustness of electrosynthetic reactions. Utilizing Glorius’ truncated robustness screen^[16c]^ chemistry we screened substrate **1 q** under our standard batch reactor conditions and observed an overall good level of tolerance against a range of basic, nucleophilic, and electrophilic additives (Scheme 3). The standard yield of the reaction as highlighted in Table 2 was 81 %, the average yield (by GC–MS) from the 16 additive reactions studied was 64 % which equates to a functional group robustness of 79 %os (percent of the standard yield), which was fairly consistent across the basic (73 %), nucleophilic (82 %), and electrophilic (76 %) additives.^[17]^ The percentage of additives unreacted was somewhat lower, showing that whilst the desired reaction **1 q** to **2 q** was only marginally affected by the additives, the additives themselves could undergo other reactions under the electrochemical conditions. For example, aniline **3** was completely consumed under the reaction conditions although **2 q** was produced in 62 %. Further examination with a blank reaction in the absence of **1 q** but with aniline present resulted in the isolation of azobenzene **4** in 22 % unoptimized yield (Scheme 4), possibly opening up a new route to these important molecules and a complementary process to the photochemical route recently reported by Vannucci.^[18]^

**Table 2:**
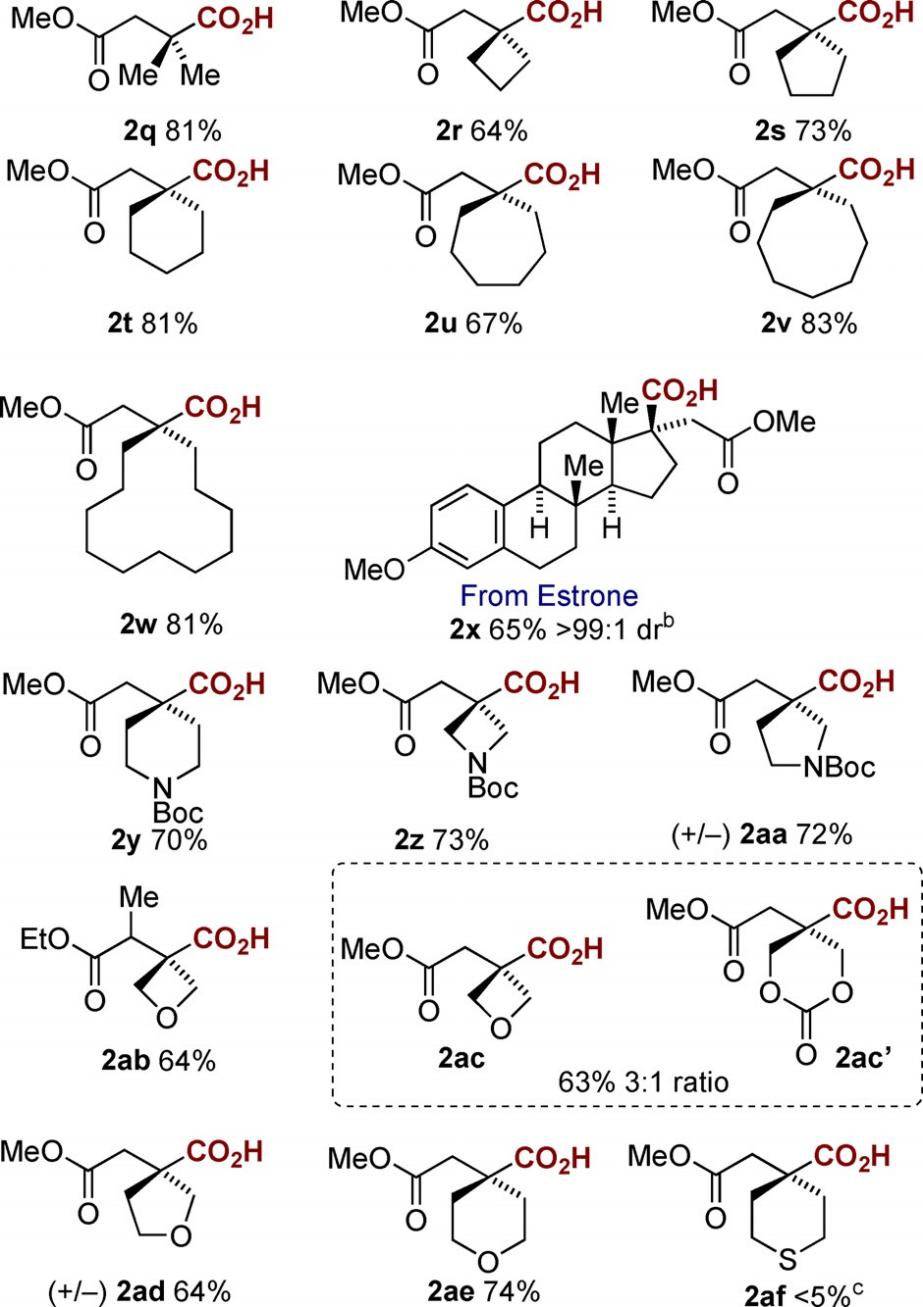
Screening of α,β‐unsaturated esters.^[a]^ 
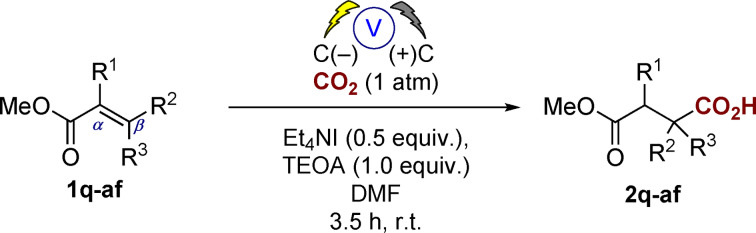

[a] General conditions: CO_2_ (1 atm), carbon anode and cathode, Et_4_NI (0.5 equiv), TEOA (1.0 equiv), DMF, single compartment cell, 10 V (60–100 mA), 4 h rt. [b] Isolated analytically pure by column chromatography. [c] Carboxylation was observed by GC–MS however we were not successful in isolating the product.

**Scheme 5:**
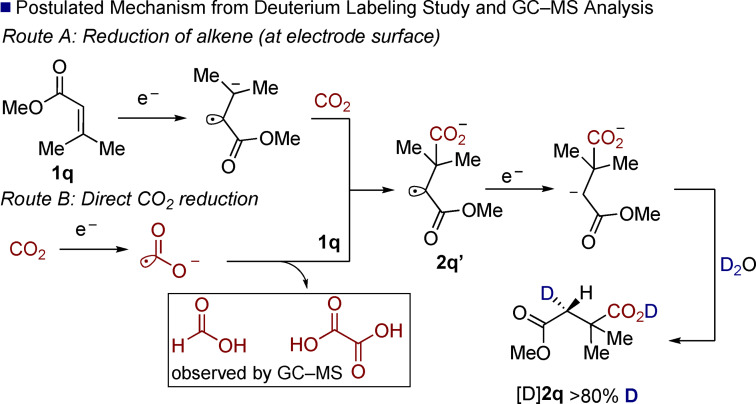
Proposed mechanistic routes to the hydrocarboxylated product after deuterium labeling study and GC–MS analysis.

We recently investigated the mechanism of hydrocarboxylation of both styrenes and dienes.[^7a^, ^7b^] The reduction potentials of CO_2_ and styrenes have been reported to be very similar, ^.^CO_2_^−^ *E*_1/2_=−2.21 V, styrene *E*_1/2_=−2.58 V vs. SCE in DMF,^[19]^ and those of the acrylates studied in this work are also in a similar range, for example, methyl acrylate **1 a** *E*_1/2_=−2.10 V and methyl crotonate **1 e** *E*_1/2_=−2.41 V vs. SCE in MeCN).^[20]^ In our previous studies, we noted that in the cathodic regime and in the absence of CO_2_, the onset of radical anion formation leads to a steep and irreversible reduction of the alkene due to cathodic polymerization, but when the solution was saturated with CO_2_,^[7a]^ the onset of the reduction process did not change, we did not observe CO_2_ reduction but carboxylation of the alkene proceeded. The ability to be able to carry out the successful reduction of the acrylates in this study in the absence of carbon dioxide points to the possible activation by addition of an initial electron to the surface‐bound alkene and subsequent carbon dioxide capture by the anion (Scheme 5). However, Jui has recently described the photochemical generation of ^.^CO_2_^−^ and that Michael acceptors that possess a more negative reduction potential undergo hydrocarboxylation while those with more positive reduction potentials undergo reduction.^[21]^ This does not fully explain our electrochemical carboxylation but there are some similarities, for example, *trans*‐cinnamate esters undergo carboxylation in low yield (**1 m**, **1 n**) and under Jui's conditions are reduced to the alkane, thus perhaps indicating that two mechanisms could be in operation (reduction of CO_2_ and/or reduction of the olefin).

Detailed investigation of the reaction mixtures by ^13^C NMR did not enable identification of oxalic acid (the CO_2_ dimerization product is known to rapidly form when ^.^CO_2_^−^ is present).^[19]^ However, examination of the reaction mixture utilizing GC–MS headspace analysis did reveal the formation of formic and oxalic acids, indicating the presence of ^.^CO_2_^−^ during the reaction (See Supporting Information for further details). We attempted to isolate adducts of TEMPO and AMBO radical traps but were unsuccessful perhaps due to the rapid reduction of these species under the reaction conditions (TEMPO−OH and AMBO−OH being readily visible by GC–MS analysis). Deuteration studies provide [D]**2 q** with high levels of deuterium incorporation when running the reaction with D_2_O instead of TEOA leading us to propose two possible mechanisms highlighted in Scheme 5. In route A the acrylate **1 q** is adsorbed to the surface of the cathode and electron transfer proceeds to form the radical anion of **1 q**, subsequent carboxylation and further electron transfer result in disassociation from the electrode and deuteration from D_2_O to afford the final mono‐carboxylated product [D]**2 q**. We know that this process could be in operation since in the absence of carbon dioxide we observe the reduced product. However, we cannot rule out route b in which direct reduction of carbon dioxide to the radical anion (for which GC–MS headspace analysis has indicated that it is present) and then subsequent addition to the olefin affords the common intermediate **2 q′**, which on addition of an electron and deuteration affords [D]**2 q**. Selectivity for carboxylation at the β‐position has been explained through DFT analysis. Lan and Yu have shown that an acrylate radical anion complex of **1 k** was calculated with higher spin density in the β‐position, which explains the high regioselectivity observed.^[3]^

## Conclusion

A highly regioselective hydrocarboxylation process that enables the direct formation of carboxylic acids from α,β‐unsaturated esters, requiring no chromatography, has been developed. The electrosynthetic system is capable of β‐carboxylation to afford all‐carbon α‐quaternary centered carboxylic acids in good yield. The process has been simply converted into flow to enable gram‐scale synthesis of a precursor to the anti‐epilepsy and absence seizure drug ethosuximide and crucially we have scrutinized our system utilizing Glorius’ reaction robustness screen to enable future benchmarking and improvement of modified electrochemical, photochemical, or transition metal systems for alkene hydrocarboxylation. In summary, the current process goes beyond the electrochemical state‐of‐the‐art enabling selective mono‐carboxylation of α,β‐unsaturated esters to afford all‐carbon quaternary centers.

## Conflict of interest

The authors declare no conflict of interest.

## Supporting information

As a service to our authors and readers, this journal provides supporting information supplied by the authors. Such materials are peer reviewed and may be re‐organized for online delivery, but are not copy‐edited or typeset. Technical support issues arising from supporting information (other than missing files) should be addressed to the authors.

Supporting Information
