# Supplementary material for: Selective Electrosynthetic Hydrocarboxylation of α,β‐Unsaturated Esters with Carbon Dioxide
Source: Angew Chem Int Ed Engl. 2021 Sep 6;60(40):21832–7. doi: 10.1002/anie.202105490 (PMC8518608; doi:10.1002/anie.202105490)
Supplement: Supplementary file 1 — Supporting Information [file ANIE-60-21832-s001.pdf]

Angewandte Chemie
Eine Zeitschrift der Gesellschaft Deutscher Chemiker

Supporting Information

Selective Electrosynthetic Hydrocarboxylation of α,β-Unsaturated Esters with Carbon Dioxide**

*Ahmed M. Sheta, Anas Alkayal, Mohammad A. Mashaly, Samy B. Said, Saad S. Elmorsy, Andrei V. Malkov, and Benjamin R. Buckley**

anie_202105490_sm_miscellaneous_information.pdf

1.0 GENERAL EXPERIMENTAL....3

2.0 PROCEDURE FOR PREPARING ACRYLATES VIA WITTIG AND HORNER–WADSWORTH–EMMONS REACTION 4

GENERAL PROCEDURE I: 4
GENERAL PROCEDURE II: 4
METHYL 3-PHENYL-2-BUTENOATE (1N).... 4
METHYL CYCLOBUTYLIDENEACETATE (1R).... 4
METHYL CYCLOPENTYLIDENEACETATE (1S) 4
METHYL CYCLOHEXYLIDENEACETATE (1T) 5
METHYL CYCLOHEPTYLIDENEACETATE (1U) 5
METHYL CYCLOOCTYLIDENEACETATE (1V) 5
METHYL CYCLODODECYLIDENEACETATE (1W).[5] 5
ESTRONE O-METHYL ETHER (1X'). 5
(Z)-METHYL 2-((8S,9S,13S,14S)-3-METHOXY-13-METHYL-7,8,9,11,12,13,15,16-OCTAHYDRO-6H-CYCLOPENTA[A]PHENANTHREN-17(14H)-YLIDENE)ACETATE (1X).... 6
TERT-BUTYL 4-(2-METHOXY-2-OXOETHYLIDENE)PIPERIDINE-1-CARBOXYLATE (1Y) 6
TERT-BUTYL 3-(2-METHOXY-2-OXOETHYLIDENE)AZETIDINE-1-CARBOXYLATE (1Z). 6
(E,Z) TERT-BUTYL 3-(2-METHOXY-2-OXOETHYLIDENE)PYRROLIDINE-1-CARBOXYLATE (1AA).... 6
ETHYL 2-(OXETAN-3-YLIDENE)PROPANOATE (1AB). 6
METHYL 2-(3-OXETANYLIDENE)ACETATE (1AC). 7
METHYL 2-(DIHYDRO-3(2H)-FURANYLIDENEACETATE (1AD). 7
METHYL 2-(TETRAHYDRO-4H-PYRAN-4-YLIDENE)ACETATE (1AE). 7
METHYL 3-METHYL-2-PENTENOATE (1AG). 7

3.0 ELECTROCARBOXYLATION OF ACRYLATES USING A NON-SACRIFICIAL ELECTROCHEMICAL CELL 8

GENERAL PROCEDURE III: 8
BUTANEDIOIC ACID 1-METHYL ESTER (2A). 8
2-METHYL BUTANEDIOIC ACID 4-METHYL ESTER (2E) 8
2-METHYL BUTANEDIOIC ACID 1-METHYL ESTER (2F). 8
2-METHYL BUTANEDIOIC ACID (2G). 8
BUTANEDIOIC ACID (2G'). 8
2-METHYL BUTANEDIOIC ACID 1-ETHYL ESTER (2H).... 9
2-METHYL BUTANEDIOIC ACID 1-BENZYL ESTER (2I). 9
2-METHYL BUTANEDIOIC ACID 1-ALLYL ESTER (2J). 9
2-METHYL BUTANEDIOIC ACID 1-(TERT-BUTYL) ESTER (2K).... 9
2-(METHOXYCARBONYL)CYCLOHEXANE-1-CARBOXYLIC ACID (2L). 9
2-PHENYL BUTANEDIOIC ACID 4-METHYL ESTER (2M). 9
2-METHYL-2-PHENYL BUTANEDIOIC ACID 4-METHYL ESTER (2N). 10
4-(2-(ISOBUTYRYLOXY)ETHOXY)-3-METHYL-4-OXOBUTANOIC ACID (2P). 10
2,2-DIMETHYL BUTANEDIOIC ACID 4-METHYL ESTER (2Q). 10
METHYL 1-CARBOXYCYCLOBUTANEACETATE (2R). 10
METHYL 1-CARBOXYCYCLOPENTANEACETATE (2S). 10
METHYL 1-CARBOXYCYCLOHEXANEACETATE (2T). 10
METHYL 1-CARBOXYCYCLOHEPTANEACETATE (2U). 11
METHYL 1-CARBOXYCYCLOOCTANEACETATE (2V). 11
METHYL 1-CARBOXYCYCLODODECANEACETATE (2W). 11
(8S,9S,13S,14S)-3-METHOXY-17-(2-METHOXY-2-OXOETHYL)-13-METHYL-7,8,9,11,12,13,14,15,16,17-DECAHYDRO-6H-CYCLOPENTA[A]PHENANTHRENE-17-CARBOXYLIC ACID (2X). 11
1-(TERT-BUTOXYCARBONYL)-4-(2-METHOXY-2-OXOETHYL)PIPERIDINE-4-CARBOXYLIC ACID (2Y).... 12
1-(TERT-BUTOXYCARBONYL)-3-(2-METHOXY-2-OXOETHYL)AZETIDINE-3-CARBOXYLIC ACID (2Z).... 12

1-(tert-butoxycarbonyl)-3-(2-methoxy-2-oxoethyl)pyrrolidine-3-carboxylic acid (2aa). 12
3-(2-ethoxy-2-methyl-2-oxoethyl)oxetane-3-carboxylic acid (2ab). 12
3-(2-methoxy-2-oxoethyl)oxetane-3-carboxylic acid (2ac) and 5-(2-methoxy-2-oxoethyl)1,3-dioxane-2-one-5-carboxylic acid (2ac’). 12
3-(2-methoxy-2-oxoethyl)tetrahydrofuran-3-carboxylic acid (2ad). 13
4-(2-methoxy-2-oxoethyl)tetrahydro-2H-pyran-4-carboxylic acid (2ae). 13
2-Ethyl-2-methyl butanedioic acid 4-methyl ester (2ag). 13
Procedure for electrochemical flow reaction 13
Deuterium Labelling Studies 13

4.0 ROBUSTNESS SCREEN FOR THE REACTION: 15

Procedure for screening 15
Procedure for gas chromatography batch calibration 15
Calibration for reaction yield 15
Protocol for batch screening and analysis 16
2-Methyl butanedioic acid 1-(1,2-dihydroxyprop-3-yl) ester (2ah). 18

5.0 CURRENT SCREENING FOR THE REACTION: 19

Procedure for screening 19

6.0 ANALYSIS OF CO_2 REDUCTION BY GC-MS HEADSPACE ANALYSIS 19

Reaction Using Dimethylacetamide (DMA): 19
Blank DMA GC-MS Spectra: 21
Reaction Using Dimethylformamide (DMF): 23
Blank DMF GC-MS Spectra 25

7.0 REFERENCES: 28

8.0 ^{1}H AND ^{13}C NMR SPECTRA OF THE ALKENES 30

9.0 ^{1}H AND ^{13}C NMR OF THE CARBOXYLIC ACIDS 39

1.0 General Experimental

Reagents: Commercially available materials (electrolytes, reducing agents, acrylates) were used without further purification. Substrates were purchased from commercial sources and used as received. Anhydrous *N,N*-dimethylformamide and toluene were purchased from Sigma-Aldrich and dried over 3Å molecular sieves prior to use. Tetraethylammonium iodide was also purchased from Sigma-Aldrich. Deuterated solvents were purchased from Fluorochem UK Ltd.

Analytical Methods: All infrared spectra were obtained using a Perkin-Elmer Spectrum 65 FT-IR spectrophotometer; thin film spectra were acquired using sodium chloride plates. All ^{1}H and ^{13}C NMR spectra were measured at 400 and 100 MHz using a Bruker Avance 400 MHz spectrometer, a Jeol ECS 400 MHz spectrometer or at 500 and 125 MHz on a Jeol ECZ 500 MHz spectrometer. The solvent used for NMR spectroscopy was $CDCl_3$ (unless stated otherwise) using TMS (tetramethylsilane) as the internal reference. Chemical shifts are given in parts per million (ppm) and J values are given in Hertz (Hz).

Analysis by GCMS utilised a Shimadzu QP2020, GC-2010 Plus, using a 15 m x 0.25 mm DB-5 column and an electron impact low resolution mass spectrometer, acids were converted to their corresponding methyl esters using TMSdiazomethane prior to sampling. Melting points were recorded using a Stuart Scientific melting point apparatus SMP3 and are uncorrected. All chromatographic manipulations used silica gel as the adsorbent. Reactions were monitored by GCMS or using thin layer chromatography (TLC) on aluminium backed plates with Merck Kiesel 60 F254 silica gel. TLC visualised by UV radiation at a wavelength of 254 nm. Purification by column chromatography used Apollo Scientific 60 40-63µm silica gel.

Headspace GC-MS analysis of CO_2 reduction was carried out on an Agilent 5977 GC-MS with a HP5-MS column 30 m long, 0.25 mm diameter, with 5% phenyl-methyl siloxane stationary phase, 0.25 µm thick. The programme was set with a 2-minute hold time at 40 ºC followed by a ramp to 200 ºC for 10 minutes. The headspace sample was introduced via a gas tight syringe.

Electrode electrochemical reactions were carried out using a 10mL reaction vial using a carbon anode and a carbon cathode supplied by IKA and the current was supplied from an IKA ElectraSyn 2.0

2.0 Procedure for preparing acrylates via Wittig and Horner–Wadsworth–Emmons reaction

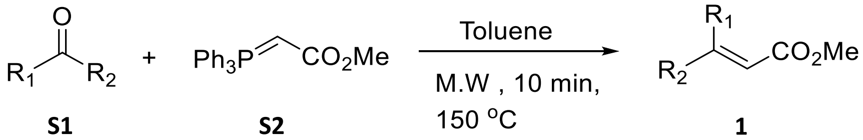

General Procedure I: In a microwave vial (2-5 mL), Methyl (triphenylphosphoranylidene)acetate **S2** (0.835 g, 2.5 mmol) and the aldehyde or ketone **S1** were dissolved in toluene (3 mL). The reaction was ran for 10 min at 150 °C. The reaction mixture was placed in a RBF (100 mL) and 5 g silica gel added. The solvent was removed under reduced pressure and the crude product was purified by column chromatography using hexane/AcOEt on silica gel affording acrylates **1**.

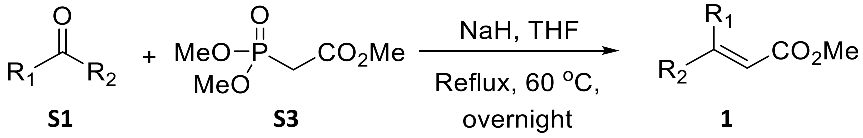

General Procedure II: In 250 mL RBF, sodium hydride (0.240 g, 10 mmol) was suspended in THF (50 mL) and trimethyl phosphonoacetate **S3** (1.62 mL, 10 mmol) was added dropwise. The reaction was then stirred under reflux for 1 hr at 60 °C then the corresponding ketone **1** (10 mmol) was dissolved in THF (10 mL) and added to the reaction carefully dropwise then the reaction was left under reflux overnight. After that, the reaction was cooled to rt, and 99% methanol (10 mL) of was added and around 10 g of silica gel. The solvent was removed under reduced pressure and the crude product was purified by column chromatography using hexane/AcOEt on silica gel affording acrylates **1**.

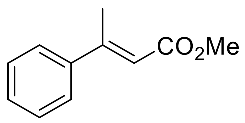

1n

Methyl 3-phenyl-2-butenoate (1n).[1] Following the general procedure II using Acetophenone (1.16 mL, 10.0 mmol), affording **1n** (1.50 g, 85.2% yield). Colorless liquid.
^{1}H NMR (400 MHz, $CDCl_3$) δ 7.46 (m, 2H), 7.36 (m, 3H), 6.13 (s, 1H), 3.74 (s, 3H), 2.57 (s, 3H) ppm. **^{13}C NMR** (101 MHz, $CDCl_3$) δ 167.3, 155.9, 142.2, 129.1, 128.6, 126.3, 116.7, 51.1, 18.0 ppm.

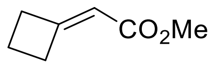

1r

Methyl cyclobutylideneacetate (1r).[2] Following the general procedure I using Cyclobutanone (0.18 mL, 2.5 mmol), affording **1r** (0.25 g, 79.3% yield). Colorless liquid
^{1}H NMR (400 MHz, $CDCl_3$) δ 5.54 (s, 1H), 3.62 (s, 3H), 3.07 (t, 2H), 2.78 (t, 2H), 2.04 (p, 2H) ppm. **^{13}C NMR** (101 MHz, $CDCl_3$) δ 167.9, 166.9, 111.9, 50.8, 33.7, 32.3, 17.6 ppm.

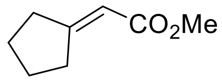

1s

Methyl cyclopentylideneacetate (1s).[3] Following the general procedure I using Cyclopentanone (0.22 mL, 2.5 mmol), affording **1s** (0.3 g, 85.7% yield). Colorless liquid
^{1}H NMR (400 MHz, $CDCl_3$) δ 5.78 (s, 1H), 3.66 (s, 3H), 2.75 (t, 2H), 2.41 (t, 2H), 1.73 (p, 2H), 1.64 (p, 2H) ppm. **^{13}C NMR** (101 MHz, $CDCl_3$) δ 169.6, 167.4, 111.2, 50.8, 36.0, 32.7, 26.4, 25.5 ppm.

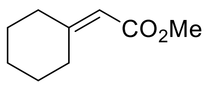

1t

Methyl cyclohexylideneacetate (1t).[3] Following the general procedure I using Cyclohexanone (0.26 mL, 2.5 mmol), affording **1t** (0.36 g, 93.5% yield). Colorless liquid
^{1}H NMR (400 MHz, $CDCl_3$) δ 5.60 (s, 1H), 3.67 (s, 3H), 2.82 (t, 2H), 2.19 (t, 2H), 1.68-1.57 (m, 6H) ppm. **^{13}C NMR** (101 MHz, $CDCl_3$) δ 167.3, 163.9, 112.6, 50.8, 38.0, 29.9, 28.5, 27.8, 26.3 ppm.

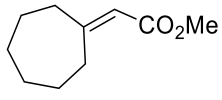

1u

Methyl cycloheptylideneacetate (1u).[2] Following the general procedure II using Cycloheptanone (1.17 mL, 10.0 mmol), affording **1u** (1.35 g, 80.3% yield). Colorless liquid
^{1}H NMR (400 MHz, $CDCl_3$) δ 5.61 (s, 1H), 3.61 (s, 3H), 2.82 (t, 2H), 2.32 (t, 2H), 1.63-1.47 (m, 8H) ppm. **^{13}C NMR** (101 MHz, $CDCl_3$) δ 167.0, 115.1, 50.6, 39.0, 32.1, 29.8, 29.0, 28.0, 26.6 ppm.

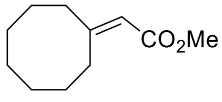

1v

Methyl cyclooctylideneacetate (1v).[4] Following the general procedure II using Cyclooctanone (1.32 mL, 10.0 mmol), affording **1v** (1.40 g, 76.9% yield). Colorless liquid
^{1}H NMR (400 MHz, $CDCl_3$) δ 5.68 (s, 1H), 3.63 (s, 3H), 2.72 (t, 2H), 2.27 (t, 2H), 1.76-1.70 (m, 4H), 1.48-1.39 (m, 8H) ppm. **^{13}C NMR** (101 MHz, $CDCl_3$) δ 168.8, 166.7, 115.1, 50.6, 38.7, 30.7, 27.8, 27.6, 26.5, 25.6, 25.4 ppm.

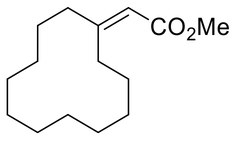

1w

Methyl cyclododecylideneacetate (1w).[5] Following the general procedure II using Cyclododecanone (1.82 g, 10.0 mmol), affording **1w** (1.60 g, 67.2% yield). White solid m.p. 65 °C (No Lit. in Ref.[5]).**^{1}H NMR** (400 MHz, $CDCl_3$) δ 5.73 (s, 1H), 3.66 (s, 3H), 2.70 (t, 2H), 2.20 (t, 2H), 1.61-1.50 (m, 4H), 1.32-1.27 (m, 14H) ppm. **^{13}C NMR** (101 MHz, $CDCl_3$) δ 167.3, 163.6, 115.8, 50.8, 32.8, 29.9, 25.1, 24.9, 24.1, 24.0, 23.8, 23.5, 23.0, 22.1 ppm. **IR** (neat, cm^{-1}) 2928, 2850, 1715, 1641, 1468, 1313, 1151.

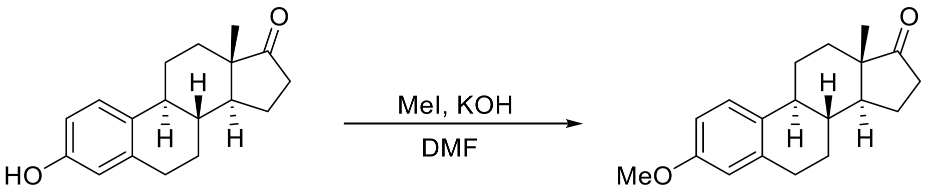

1x'

Estrone O-methyl ether (1x').[6] A solution of Estrone (2.70 g, 10 mmol) in dimethylformamide (50 mL) was treated with ground potassium hydroxide (0.67 g, 12 mmol) and iodomethane (0.75 mL, 12 mmol) in 20 mL DMF (dropwise over 5 min at 0 °C). the reaction was stirred at room temperature for 30 min and, after this time period, additional ground potassium hydroxide (0.67 g, 12 mmol) and iodomethane (0.75 mL, 12 mmol) in 20 mL DMF were added at 0 °C. The reaction was then left to stir for 3 h. The solution was poured onto ice and extracted with ethyl acetate (3x50 mL). The organic layers were washed with water, brine and dried by $MgSO_4$. The solvent was removed under reduced pressure to yield **1x'** (2.80 g, 98.6%). White solid m.p 170 °C. **^{1}H NMR** (400 MHz, $CDCl_3$) δ 7.19 (d, 1H), 6.70 (m, 2H), 3.77 (s, 3H), 2.89 (m, 2H), 2.52-1.92 (m, 7H), 1.65-1.37 (m, 6H), 0.89 (s, 3H) ppm. **^{13}C NMR** (101 MHz, $CDCl_3$) δ 221.0, 157.6, 137.8, 132.1, 126.4, 113.9, 111.6, 55.3, 50.4, 48.1, 44.0, 38.4, 35.9, 31.6, 29.7, 26.6, 26.0, 21.6, 13.9 ppm.

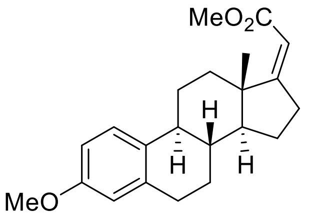

1x

(Z)-methyl 2-((8S,9S,13S,14S)-3-methoxy-13-methyl-7,8,9,11,12,13,15,16-octahydro-6H-cyclopenta[a]phenanthren-17(14H)-ylidene)acetate (1x). Following the general procedure II using Estrone O-methyl ether **(1x')** (2.84 g, 10.0 mmol), affording **1x** (2.0 g, 58.8% yield). White solid m.p. 150 °C.

^{1}H NMR (400 MHz, $CDCl_3$) δ 7.19 (d, 1H), 6.67 (m, 2H), 5.67 (s, 1H), 3.76 (s, 3H), 3.68 (s, 3H), 2.85 (m, 3H), 2.60 (m, 1H), 2.46 (m, 1H), 2.30 (m, 1H), 2.25 (m, 1H), 1.19 (m, 1H), 1.18 (m, 1H), 1.60-1.20 (m, 6H), 1.02 (s, 3H) ppm. **^{13}C NMR** (101 MHz, $CDCl_3$) δ 171.9, 166.5, 157.5, 137.8, 132.7, 126.3, 113.8, 111.5, 111.1, 55.4, 55.2, 51.0, 46.2, 43.7, 38.3, 34.1, 33.9, 29.8, 27.7, 26.8, 23.5, 15.7 ppm. **IR** (neat, cm^{-1}) 2980, 2925, 2877, 1713, 1646, 1437, 1171. **HRMS** ESI, ($C_{22}H_{28}NaO_3$) $[M+Na]^+$ *calculated* 363.1931, *found* 363.1930.

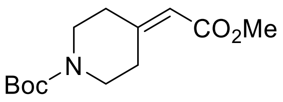

1y

tert-Butyl 4-(2-methoxy-2-oxoethylidene)piperidine-1-carboxylate (1y).[7] Following the general procedure I using 1-Boc-4-piperidone (0.49 g, 2.5 mmol), affording **1y** (0.51 g, 79.6% yield). White solid m.p. 61 °C.

^{1}H NMR (400 MHz, $CDCl_3$) δ 5.68 (s, 1H), 3.66 (s, 3H), 3.45 (p, 4H), 2.90 (t, 2H), 2.25 (t, 2H), 1.43 (s, 9H) ppm. **^{13}C NMR** (101 MHz, $CDCl_3$) δ 166.8, 158.3, 154.6, 114.8,79.9, 51.0, 36.5, 29.6, 28.4 ppm.

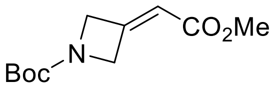

1z

tert-Butyl 3-(2-methoxy-2-oxoethylidene)azetidine-1-carboxylate (1z).[8] Following the general procedure I using 1-Boc-3-azetidinone (0.43 g, 2.5 mmol), affording **1z** (0.45 g, 80.3% yield). White solid m.p. 45 °C.

^{1}H NMR (400 MHz, $CDCl_3$) δ 5.76 (s, 1H), 4.79 (dd, J = 6.4, 2.8 Hz, 2H), 4.57 (dd, J = 5.4, 3.3 Hz, 2H), 3.70 (s, 3H), 1.43 (s, 9H) ppm. **^{13}C NMR** (101 MHz, $CDCl_3$) δ 165.7, 156.2, 153.1, 113.3, 80.1, 60.3, 57.8, 51.5, 28.4 ppm.

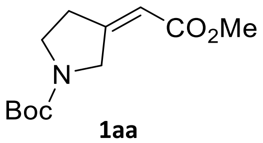

1aa

(E,Z) tert-Butyl 3-(2-methoxy-2-oxoethylidene)pyrrolidine-1-carboxylate (1aa).[9] Following the general procedure I using 1-Boc-3-pyrrolidinone (0.46 g, 2.5 mmol), affording **1aa** (0.5 g, 83.3% yield) *E/Z* ratio 1:1. Yellow liquid.

^{1}H NMR (400 MHz, $CDCl_3$) δ 5.80 (s, 1H), 4.40 (s,1H), 4.10 (s,1H), 3.69 (s, 3H), 3.54 (d, 2H), 3.10 (t, 1H), 2.72 (t, 1H), 1.45 (d, 9H) ppm. **^{13}C NMR** (101 MHz, $CDCl_3$) δ 166.6, 160.9, 154.3, 112.2, 79.8, 51.7, 51.3, 51.2, 50.5, 28.5 ppm.

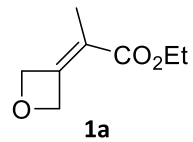

1a

Ethyl 2-(oxetan-3-ylidene)propanoate (1ab). Following the general procedure I using 3-Oxetanone (0.16 mL, 2.5 mmol) and Ethyl 2-(triphenylphosphoranylidene)propionate (0.9 g, 2.5 mmole), affording **1ab** (0.25 g, 64.1% yield). Colorless liquid.

^{1}H NMR (400 MHz, $CDCl_3$) δ 5.33 (m, 2H), 5.19 (m, 2H), 4.09 (q, 2H), 1.61 (s, 3H), 1.20 (t, 3H) ppm. **^{13}C NMR** (101 MHz, $CDCl_3$) δ 166.3, 151.1, 118.5, 80.8, 77.9, 60.5, 14.3, 12.7 ppm. **IR** (neat, cm^{-1}) 2981, 2927, 2857, 1728, 1710, 1447, 1295, 1195. **HRMS** ESI, ($C_8H_{12}NaO_3$) $[M+Na]^+$ *calculated* 179.0679, *found* 179.0679.

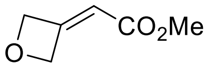

1ac

Methyl 2-(3-oxetanylidene)acetate (1ac).[10] Following the general procedure I using 3-Oxetanone (0.16 mL, 2.5 mmol), affording **1ac** (0.22 g, 68.7% yield). White solid m.p. 51 ^oC.

^{1}**H NMR** (400 MHz, $CDCl_3$) δ 5.64 (s, 1H), 5.50 (dd, *J* = 6.4, 2.8 Hz, 2H), 5.30 (dd, *J* = 5.6, 2 Hz, 2H), 3.70 (s, 3H) ppm. ^{13}**C NMR** (101 MHz, $CDCl_3$) δ 165.7, 159.6, 110.8, 81.1, 78.5, 51.5 ppm.

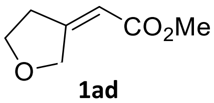

1ad

Methyl 2-(dihydro-3(2H)-furanylideneacetate (1ad).[11] Following the general procedure I using Tetrahydro-3-furanone (0.19 mL, 2.5 mmol), affording **1ad** (0.28 g, 80.0% yield) *E/Z* ratio 2.5:1. Colorless liquid

^{1}**H NMR** (400 MHz, $CDCl_3$) δ 5.79 (s, 1H, *E* isomer), 5.74 (s, 1H, *Z* isomer), 4.64 (d, *J* = 2 Hz, 2H, *E* isomer), 4.31 (d, *J* = 2 Hz, 2H, *Z* isomer), 3.82 (m, 2H, *Z* isomer), 3.79 (m, 2H, *E* isomer), 3.64 (s, 3H, *Z* isomer), 3.63 (s, 3H, *E* isomer), 2.96 (m, 2H, *Z* isomer), 2.65 (m, 2H, *E* isomer) ppm. ^{13}**C NMR** (101 MHz, $CDCl_3$) δ 166.7, 166.5, 163.2, 162.2, 111.2, 110.9, 110.5, 109.8, 72.0, 71.5, 68.9, 67.0, 51.2, 51.1, 34.4, 34.2, 32.6 ppm.

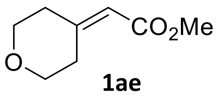

1ae

Methyl 2-(tetrahydro-4H-pyran-4-ylidene)acetate (1ae).[12] Following the general procedure I using Tetrahydro-4H-pyran-4-one (0.23 mL, 2.5 mmol), affording **1ae** (0.3 g, 76.9% yield). Colorless liquid

^{1}**H NMR** (400 MHz, $CDCl_3$) δ 5.63 (s, 1H), 3.70 (m, 4H), 3.63 (s, 3H), 2.95 (t, 2H), 2.28 (t, 2H) ppm. ^{13}**C NMR** (101 MHz, $CDCl_3$) δ 166.8, 157.6, 114.1, 69.0, 68.5, 51.0, 37.6, 31.1 ppm.

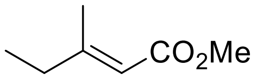

1ag

Methyl 3-methyl-2-pentenoate (1ag).[13] Following the general procedure II using Butanone (0.89 mL, 10.0 mmol), affording **1ag** (1.0 g, 78.1% yield) with *E:Z* ratio 1.7:1. Colorless liquid.

^{1}**H NMR** (400 MHz, $CDCl_3$) δ 5.56 (s, 1H, *E* isomer), 5.53 (s, 1H, *Z* isomer), 3.58 (s, 3H, *E* isomer), 3.57 (s, 3H, *Z* isomer), 2.53 (q, 2H, *Z* isomer), 2.07 (m, 5H, CH_2&CH_3 for *E&Z* isomers), 1.78 (s, 3H, *Z* isomer), 0.97 (t, 3H) ppm. ^{13}**C NMR** (101 MHz, $CDCl_3$) δ 167.2, 166.6, 162.3, 161.7, 114.9, 114.0, 50.6, 33.6, 26.4, 24.5, 18.6, 12.4, 11.8 ppm.

3.0 Electrocarboxylation of acrylates using a non-sacrificial electrochemical cell

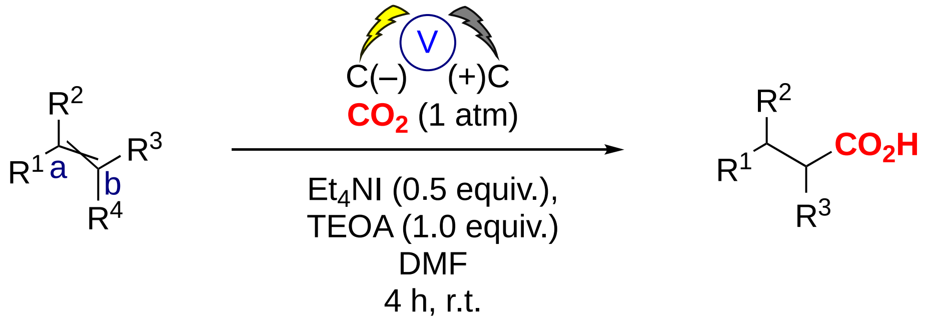

General Procedure III: Substrate (1.0 mmol) was added to a solution of Et_4NI (128 mg, 0.5 mmol) and TEOA (149 mg, 1.0 mmol) in DMF (5 mL). The resulting mixture was flushed with CO_2 and kept under a positive pressure of CO_2. The mixture was electrolysed at a constant voltage of 10V in a single compartment cell containing carbon cathode and carbon anode with stirring for 4h. The crude reaction mixture was acidified by addition of HCl/H_2O (1:1, 2 mL) and extracted with diethylether (3 x 5mL). The combined organic phases were washed with brine, dried over $MgSO_4$ and filtered. The solvent was then removed under reduced pressure and the crude material was recrystalised by hexane.

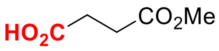

2a

Butanedioic acid 1-methyl ester (2a).[14] Following the general procedure III using Methyl acrylate (86 mg, 1.0 mmol), affording **2a** (100 mg, 75.7%). White solid m.p. 55 °C.

^{1}H NMR (400 MHz, $CDCl_3$) δ 3.69 (s, 3H), 2.69-2.60 (m, 4H) ppm. **^{13}C NMR** (101 MHz, $CDCl_3$) δ 177.3, 172.7, 52.0, 28.8, 28.7 ppm.

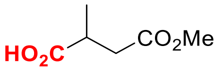

2e

2-Methyl butanedioic acid 4-methyl ester (2e).[15] Following the general procedure III using Methyl crotonate (100 mg, 1.0 mmol), affording **2e** (115 mg, 78.7% yield). Colorless oil.

^{1}H NMR (400 MHz, $CDCl_3$) δ 3.65 (s, 3H), 2.74 (m, 1H), 2.70 (dd, J = 8, 16.8 *Hz*, 1H), 2.40 (dd, J = 6.4, 16.8 *Hz*, 1H), 1.22 (d, 3H) ppm. **^{13}C NMR** (101 MHz, $CDCl_3$) δ 181.2, 172.3, 51.9, 37.1, 35.7, 16.8 ppm.

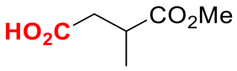

2f

2-Methyl butanedioic acid 1-methyl ester (2f).[16] Following the general procedure III using Methyl methacrylate (100 mg, 1.0 mmol), affording **2f** (110 mg, 75.3% yield). Colorless oil.

^{1}H NMR (400 MHz, $CDCl_3$) δ 3.68 (s, 3H), 2.81 (m, 1H), 2.77 (dd, J = 6.4, 13.6 *Hz*, 1H), 2.43 (dd, J = 4.8, 13.6 *Hz*, 1H), 1.22 (d, 3H) ppm. **^{13}C NMR** (101 MHz, $CDCl_3$) δ 177.7, 175.7, 52.1, 37.3, 35.5, 17.0 ppm.

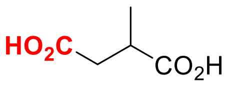

2g

2-Methyl butanedioic acid (2g).[17] Following the general procedure III using Methacrylic acid (86 mg, 1.0 mmol), affording **2g** (65 mg, 49.2% yield). White solid m.p 110 °C.

^{1}H NMR (400 MHz, D_2O) δ 2.75 (m, 1H), 2.55 (m, 1H), 2.42 (m, 1H), 1.06 (d, 3H) ppm. **^{13}C NMR** (101 MHz, D_2O) δ 180.5, 176.6, 37.3, 35.8, 16.2 ppm.

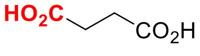

2g'

Butanedioic acid (2g').[18] Following the general procedure III using Acrylic acid (72 mg, 1.0 mmol), affording **2g'** (55 mg, 46.6% yield). White solid m.p 180 °C.

^{1}**H NMR** (400 MHz, D_2O) δ 2.57 (s, 4H) ppm. ^{13}**C NMR** (101 MHz, D_2O) δ 177.1, 28.8 ppm.

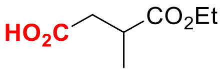
2h

2-Methyl butanedioic acid 1-ethyl ester (2h).[19] Following the general procedure III using Ethyl methacrylate (114 mg, 1.0 mmol), affording **2h** (115 mg, 71.8% yield). Colorless oil.

^{1}**H NMR** (400 MHz, $CDCl_3$) δ 4.12 (q, 2H), 2.86 (m, 1H), 2.74 (dd, *J* = 8.4, 17.2 *Hz*, 1H), 2.42 (dd, *J* = 6.0, 16.8 *Hz*, 1H), 1.23 (m, 6H) ppm. ^{13}**C NMR** (101 MHz, $CDCl_3$) δ 177.6, 175.2, 60.9, 37.3, 35.6, 17.0, 14.1 ppm.

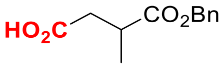
2i

2-Methyl butanedioic acid 1-benzyl ester (2i).[20] Following the general procedure III using Benzyl methacrylate (176 mg, 1.0 mmol), affording **2i** (150 mg, 67.5% yield). Colorless oil.

^{1}**H NMR** (400 MHz, $CDCl_3$) δ 7.36-7.29 (m, 5H), 5.13 (s, 2H), 2.94 (m, 1H), 2.80 (dd, *J* = 8.4, 16.8 *Hz*, 1H), 2.46 (dd, *J* = 6.0, 16.8 *Hz*, 1H), 1.25 (d, 3H) ppm. ^{13}**C NMR** (101 MHz, $CDCl_3$) δ 176.5, 175.0, 135.9, 128.6, 128.2, 128.1, 66.6, 37.1, 35.7, 17.0 ppm.

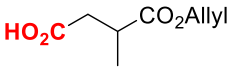
2j

2-Methyl butanedioic acid 1-allyl ester (2j). Following the general procedure III using Allyl methacrylate (126 mg, 1.0 mmol), affording **2j** (115 mg, 66.8% yield). Colorless oil.

^{1}**H NMR** (400 MHz, $CDCl_3$) δ 5.92-5.84 (m, 1H), 5.32-5.20 (m, 2H), 4.57 (d, 2H), 2.90 (m, 1H), 2.78 (dd, *J* = 6.8, 13.6 *Hz*, 1H), 2.45 (dd, *J* = 4.4, 12.9 *Hz*, 1H), 1.23 (d, 3H) ppm. ^{13}**C NMR** (101 MHz, $CDCl_3$) δ 177.6, 174.8, 132.0, 118.3, 65.5, 37.3, 35.6, 17.0 ppm. **IR** (neat, cm^{-1}) 3453, 2982, 2943, 1714, 1649, 1338, 1177. **HRMS** ESI, ($C_8H_{12}NaO_4$) $[M+Na]^+$ *calculated* 195.0628, *found* 195.0629.

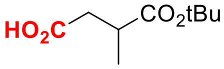
2k

2-Methyl butanedioic acid 1-(tert-butyl) ester (2k).[21] Following the general procedure III using *tert*-Butyl methacrylate (142 mg, 1.0 mmol), affording **2k** (135 mg, 71.8% yield). Colorless oil.

^{1}**H NMR** (400 MHz, $CDCl_3$) δ 2.73 (m, 1H), 2.65 (dd, *J* = 8.4, 16.2 *Hz*, 1H), 2.35 (dd, *J* = 5.6, 16.8 *Hz*, 1H), 1.39 (s, 9H), 1.15 (d, 3H) ppm. ^{13}**C NMR** (101 MHz, $CDCl_3$) δ 178.1, 174.5, 80.8, 37.5, 36.5, 27.9, 17.0 ppm.

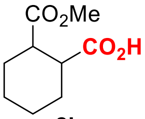
2l

2-(methoxycarbonyl)cyclohexane-1-carboxylic acid (2l).[22] Following the general procedure III using Methyl 1-cyclohexene-1-carboxylate (140 mg, 1.0 mmol), affording **2l** (140 mg, 75.2% yield). Colorless oil.

^{1}**H NMR** (400 MHz, $CDCl_3$) δ 3.63 (s, 3H), 2.80 (m, 1H), 2.56 (m, 1H), 1.97 (m, 2H), 1.73 (m, 2H), 1.50-1.25 (m, 4H) ppm. ^{13}**C NMR** (101 MHz, $CDCl_3$) δ 181.0, 179.9, 175.6, 174.2, 51.9, 51.7, 44.6, 44.5, 42.5, 42.3, 28.8, 26.2, 26.0, 25.2, 25.1, 23.8, 23.6 ppm.

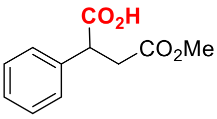
2m

2-Phenyl butanedioic acid 4-methyl ester (2m).[23] Following the general procedure III using Methyl cinnamate (162 mg, 1.0 mmol), affording **2m** (80 mg, 38.5% yield) purified by preparative TLC (40% EtOAc/Hexane). Colorless oil.

^{1}**H NMR** (500 MHz, $CDCl_3$) δ 7.34-7.26 (m, 5H), 4.11 (dd, *J* = 5.5, 10.0 *Hz*, 1H), 3.66 (s, 3H), 3.15 (dd, *J* = 10.5, 17.0 *Hz*, 1H), 2.67 (dd, *J* = 5.5, 12.0 *Hz*, 1H) ppm. ^{13}**C NMR** (125 MHz, $CDCl_3$) δ 177.7, 171.9, 137.1, 129.0, 128.0, 127.9, 52.0, 47.0, 37.2 ppm.

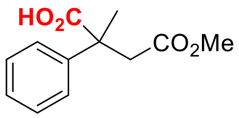

2n

2-Methyl-2-phenyl butanedioic acid 4-methyl ester (2n). Following the general procedure III using Methyl 3-phenyl-2-butenoate **1n** (176 mg, 1.0 mmol), affording **2n** (50 mg, 22.5% yield) purified by preparative TLC (40% EtOAc/Hexane). Colorless oil.

^{1}H NMR (400 MHz, $CDCl_3$) δ 7.39-7.17 (m, 5H), 3.63 (s, 3H), 3.26 (d, *J* = 16.4 *Hz*, 1H), 2.83 (d, *J* = 16.4 *Hz*, 1H), 17.17 (s, 3H) ppm. **^{13}C NMR** (101 MHz, $CDCl_3$) δ 180.5, 171.5, 141.8, 128.7, 127.5, 125.8, 51.8, 48.1, 43.2, 23.1 ppm. **IR** (neat, cm^{-1}) 3583, 3061, 3027, 2991, 2953, 1739, 1706, 1438, 1296, 1171. **HRMS** ESI, ($C_{12}H_{14}NaO_4$) $[M+Na]^+$ *calculated* 245.0784, *found* 245.0785.

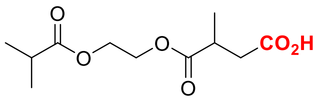

2p

4-(2-(isobutyryloxy)ethoxy)-3-methyl-4-oxobutanoic acid (2p). Following the general procedure III using Ethylene glycol dimethacrylate **1p** (198 mg, 1.0 mmol), affording **2p** (150 mg, 60.9% yield). Yellow liquid.

^{1}H NMR (400 MHz, $CDCl_3$) δ 4.28 (m, 4H), 2.89 (m, 1H), 2.77 (m, 1H), 2.52 (m, 2H), 1.22 (d, 3H), 1.14 (d, 6H) ppm. **^{13}C NMR** (101 MHz, $CDCl_3$) δ 176.9, 174.8, 62.6, 61.9, 37.1, 35.5, 33.9, 18.9, 16.9 ppm. **IR** (neat, cm^{-1}) 2976, 2937, 2882, 1738, 1713, 1278, 1159, 1057. **HRMS** ESI, ($C_{11}H_{18}NaO_6$) $[M+Na]^+$ *calculated* 269.0996, *found* 269.0995.

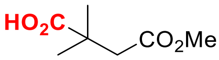

2q

2,2-Dimethyl butanedioic acid 4-methyl ester (2q).[24] Following the general procedure III using Methyl 3,3-dimethylacrylate (114 mg, 1.0 mmol), affording **2q** (130 mg, 81.2% yield). White solid m.p. 40 °C.

^{1}H NMR (400 MHz, $CDCl_3$) δ 3.65 (s, 3H), 2.60 (s, 2H), 1.28 (s, 6H) ppm. **^{13}C NMR** (101 MHz, $CDCl_3$) δ 183.0, 171.7, 51.6, 43.8, 40.5, 25.2 ppm.

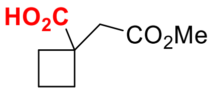

2r

Methyl 1-carboxycyclobutaneacetate (2r). Following the general procedure III using Methyl cyclobutylideneacetate **1r** (126 mg, 1.0 mmol), affording **2r** (110 mg, 63.9% yield). Colorless liquid.

^{1}H NMR (400 MHz, $CDCl_3$) δ 10.81 (s, 1H), 3.62 (s, 3H), 2.83 (s, 2H), 2.55 (m, 2H), 2.02-1.93 (m, 4H) ppm. **^{13}C NMR** (101 MHz, $CDCl_3$) δ 182.2, 171.9, 51.7, 44.5, 41.1, 29.9, 15.8 ppm. **IR** (neat, cm^{-1}) 3434, 1728, 1699, 1432, 1187. **HRMS** ESI, ($C_8H_{12}NaO_4$) $[M+Na]^+$ *calculated* 195.0628, *found* 195.0630.

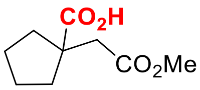

2s

Methyl 1-carboxycyclopentaneacetate (2s). Following the general procedure III using Methyl cyclopentylideneacetate **1s** (140 mg, 1.0 mmol), affording **2s** (135 mg, 72.5% yield). White solid m.p. 60 °C.

^{1}H NMR (400 MHz, $CDCl_3$) δ 3.65 (s, 3H), 2.69 (s, 2H), 2.21 (m, 2H), 1.75-1.57 (m, 6H) ppm. **^{13}C NMR** (101 MHz, $CDCl_3$) δ 182.5, 172.1, 51.7, 50.6, 42.4, 36.8, 25.4 ppm. **IR** (neat, cm^{-1}) 3434, 2958, 1732, 1691, 1643, 1438, 1198. **HRMS** ESI, ($C_9H_{14}NaO_4$) $[M+Na]^+$ *calculated* 209.0784, *found* 209.0785.

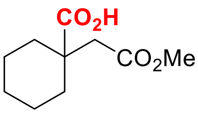

2t

Methyl 1-carboxycyclohexaneacetate (2t). Following the general procedure III using Methyl cyclohexylideneacetate **1t** (154 mg, 1.0 mmol), affording **2t** (162 mg, 81.0% yield). White solid m.p. 58 °C.

^{1}H NMR (400 MHz, $CDCl_3$) δ 11.1 (s, 1H), 3.62 (s, 3H), 2.62 (s, 2H), 1.96 (m, 2H), 1.54-1.35 (m, 8H) ppm. **^{13}C NMR** (101 MHz, $CDCl_3$) δ 182.6, 171.5, 51.6,

44.7, 42.4, 33.4, 25.5, 22.3 ppm. **IR** (neat, cm^{-1}) 3413, 2980, 1720, 1704, 1640, 1420, 1150. **HRMS** ESI, ($C_{10}H_{16}NaO_4$) $[M+Na]^+$ *calculated* 223.0941, *found* 223.0941.

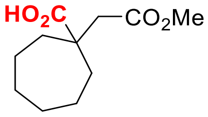

2u

Methyl 1-carboxycycloheptaneacetate (2u). Following the general procedure III using Methyl cycloheptylideneacetate **1u** (168 mg, 1.0 mmol), affording **2u** (145 mg, 67.7% yield). White solid m.p. 61 °C.

^{1}H NMR (400 MHz, $CDCl_3$) δ 3.63 (s, 3H), 2.61 (s, 2H), 2.10 (m, 2H), 1.62-1.42 (m, 10H) ppm. **^{13}C NMR** (101 MHz, $CDCl_3$) δ 183.3, 171.7, 51.6, 47.5, 43.5, 35.4, 30.4, 23.4 ppm. **IR** (neat, cm^{-1}) 3428, 2928, 2856, 1711, 1698, 1438, 1197. **HRMS** ESI, ($C_{11}H_{18}NaO_4$) $[M+Na]^+$ *calculated* 237.1097, *found* 237.1098.

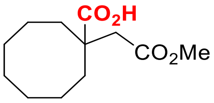

2v

Methyl 1-carboxycyclooctaneacetate (2v). Following the general procedure III using Methyl cyclooctylideneacetate **1v** (182 mg, 1.0 mmol), affording **2v** (190 mg, 83.3% yield). White solid m.p. 93 °C.

^{1}H NMR (400 MHz, $CDCl_3$) δ 3.63 (s, 3H), 2.61 (s, 2H), 2.04 (m, 2H), 1.56-1.48 (m, 12H) ppm. **^{13}C NMR** (101 MHz, $CDCl_3$) δ 182.6, 171.6, 51.6, 47.5, 42.0, 30.7, 28.3, 25.3, 22.9 ppm. **IR** (neat, cm^{-1}) 3440, 2999, 2987, 1745, 1713, 1476, 1170. **HRMS** ESI, ($C_{12}H_{20}NaO_4$) $[M+Na]^+$ *calculated* 251.1254, *found* 251.1254.

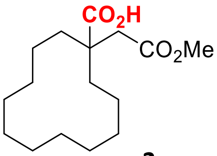

2w

Methyl 1-carboxycyclododecaneacetate (2w). Following the general procedure III using Methyl cyclododecylideneacetate **1w** (238 mg, 1.0 mmol), affording **2w** (230 mg, 80.9% yield). White solid m.p. 120 °C.

^{1}H NMR (400 MHz, $CDCl_3$) δ 3.64 (s, 3H), 2.60 (s, 2H), 1.66 (m, 4H), 1.32-1.22 (m, 18H) ppm. **^{13}C NMR** (101 MHz, $CDCl_3$) δ 181.8, 171.5, 51.6, 46.5, 41.1, 29.4, 26.2, 26.1, 22.3, 21.8, 18.9 ppm. **IR** (neat, cm^{-1}) 3435, 2932, 2862, 1739, 1701, 1471, 1198. **HRMS** ESI, ($C_{16}H_{28}NaO_4$) $[M+Na]^+$ *calculated* 307.1880, *found* 307.1880.

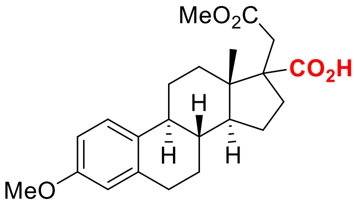

2x

(8S,9S,13S,14S)-3-methoxy-17-(2-methoxy-2-oxoethyl)-13-methyl-7,8,9,11,12,13,14,15,16,17-decahydro-6H-cyclopenta[a]phenanthrene-17-carboxylic acid (2x).

Following the general procedure III using (Z)-methyl 2-((8S,9S,13S,14S)-3-methoxy-13-methyl-7,8,9,11,12,13,15,16-octahydro-6H-cyclopenta[a]phenanthren-17(14H)-ylidene)acetate **(1x)**. (340 mg, 1.0 mmol), affording **2x** (250 mg, 64.7% yield) purified by preparative TLC (40% EtOAc/Hexane). White solid m.p. 220 °C.

^{1}H NMR (400 MHz, $CDCl_3$) δ 10.65 (br, 1H), 7.11 (d, 1H), 6.64 (m, 2H), 3.76 (s, 3H), 3.65 (s, 3H), 3.01 (d, 1H), 2.83 (m, 2H), 2.74 (m, 1H), 2.42 (m, 1H), 2.21 (m, 2H), 1.90-1.30 (m, 10H), 0.82 (s, 3H) ppm. **^{13}C NMR** (101 MHz, $CDCl_3$) δ 180.8, 173.0, 157.5, 138.0, 132.4, 126.2, 113.8, 111.5, 57.7, 55.2, 51.7, 49.6, 47.1, 43.4, 39.6, 39.2, 34.5, 32.8, 29.8, 28.0, 26.4, 25.0, 15.7 ppm. **IR** (neat, cm^{-1}) 2928, 2854, 1737, 1697, 1499, 1201, 1179, 1035. **HRMS** ESI, ($C_{23}H_{30}NaO_5$) $[M+Na]^+$ *calculated* 409.1985, *found* 409. 1985.

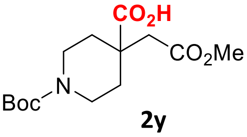

2y

1-(tert-butoxycarbonyl)-4-(2-methoxy-2-oxoethyl)piperidine-4-carboxylic acid (2y). Following the general procedure III using tert-Butyl 4-(2-methoxy-2-oxoethylidene)piperidine-1-carboxylate **1y** (255 mg, 1.0 mmol), affording **2y** (210 mg, 69.7% yield). White solid m.p. 137 °C.

^{1}H NMR (400 MHz, $CDCl_3$) δ 3.70 (m, 2H), 3.65 (s, 3H), 3.22 (m, 2H), 2.63 (s, 2H), 2.07 (m, 2H), 1.51 (m, 2H), 1.43 (s, 9H) ppm. **^{13}C NMR** (101 MHz, $CDCl_3$) δ 180.1, 170.8, 154.8, 79.8, 51.8, 43.2, 42.8, 32.8, 28.5 ppm. **IR** (neat, cm^{-1}) 3435, 1741, 1722, 1650, 1437, 1280, 1169. **HRMS** ESI, ($C_{14}H_{23}NNaO_6$) $[M+Na]^+$ *calculated* 324.1418, *found* 324.1418.

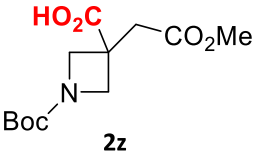

2z

1-(tert-butoxycarbonyl)-3-(2-methoxy-2-oxoethyl)azetidine-3-carboxylic acid (2z). Following the general procedure III using tert-Butyl 3-(2-methoxy-2-oxoethylidene)azetidine-1-carboxylate **1z** (227 mg, 1.0 mmol), affording **2z** (200 mg, 73.2% yield). White solid m.p. 122 °C.
^{1}H NMR (400 MHz, $CDCl_3$) δ 4.26 (br, 2H), 3.76 (d, 2H), 3.63 (s, 3H), 2.94 (s, 2H), 1.39 (s, 9H) ppm. **^{13}C NMR** (101 MHz, $CDCl_3$) δ 176.8, 171.2, 156.4, 80.5, 56.3, 52.1, 39.5, 39.4, 28.3 ppm. **IR** (neat, cm^{-1}) 3447, 2979, 2894, 1735, 1698, 1695, 1436, 1156. **HRMS** ESI, ($C_{12}H_{19}NNaO_6$) $[M+Na]^+$ *calculated* 296.1105, *found* 296.1105.

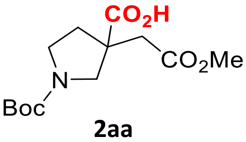

2aa

1-(tert-butoxycarbonyl)-3-(2-methoxy-2-oxoethyl)pyrrolidine-3-carboxylic acid (2aa). Following the general procedure III using tert-Butyl 3-(2-methoxy-2-oxoethylidene)pyrrolidine-1-carboxylate **1aa** (241 mg, 1.0 mmol), affording **2aa** (208 mg, 72.4% yield). White solid m.p. 130 °C.
^{1}H NMR (400 MHz, $CDCl_3$) δ 3.88 (d, 1H), 3.67 (s, 3H), 3.46 (m, 2H), 3.29 (m, 1H), 2.75 (m, 2H), 2.38 (m, 1H), 1.90 (m, 1H), 1.43 (s, 9H) ppm. **^{13}C NMR** (101 MHz, $CDCl_3$) δ 178.9, 171.4, 154.5, 79.9, 53.7, 52.0, 50.1, 44.3, 40.1, 34.2, 28.5 ppm. **IR** (neat, cm^{-1}) 3413, 2990, 2942, 1741, 1713, 1640, 1420, 1189. **HRMS** ESI, ($C_{13}H_{21}NNaO_6$) $[M+Na]^+$ *calculated* 310.1261, *found* 310.1260.

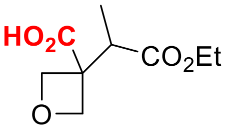

2ab

3-(2-ethoxy-2-methyl-2-oxoethyl)oxetane-3-carboxylic acid (2ab). Following the general procedure III using Ethyl 2-(oxetan-3-ylidene)propanoate **1ab** (156 mg, 1.0 mmol), affording **2ab** (130 mg, 64.3% yield). Yellow liquid.
^{1}H NMR (400 MHz, $CDCl_3$) δ 8.08 (br, 1H), 5.01 (d, 1H), 4.89 (d, 1H), 4.74 (d, 1H), 4.62 (d, 1H), 4.11 (q, 2H), 3.13 (q, 1H), 1.33 (d, 3H), 1.21 (t, 3H) ppm. **^{13}C NMR** (101 MHz, $CDCl_3$) δ 177.6, 173.6, 77.0, 75.6, 61.1, 50.2, 42.7, 14.0, 11.9 ppm. **IR** (neat, cm^{-1}) 2983, 2902, 1729, 1464, 1206, 1018. **HRMS** ESI, ($C_9H_{14}NaO_5$) $[M+Na]^+$ *calculated* 225.0733, *found* 225.0734.

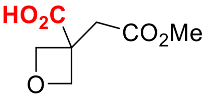

2ac

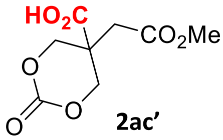

2ac'

3-(2-methoxy-2-oxoethyl)oxetane-3-carboxylic acid (2ac) and 5-(2-methoxy-2-oxoethyl)1,3-dioxane-2-one-5-carboxylic acid (2ac'). Following the general procedure III using Methyl 2-oxetanylideneacetate **1ac** (128 mg, 1.0 mmol), affording **2ac&2ac'**

(110 mg, 63.2% yield) with ratio 1:3 From ^1H NMR and repeating the reaction but using $LiBF_4$ instead of Et_4NI the ratio was 1:1.3. Colourless liquid

^{1}H NMR (400 MHz, $CDCl_3$) For 2ac δ 4.83 (d, 2H), 4.50 (d, 2H), 3.63 (s, 3H), 2.91 (s, 2H) ppm. For 2ac' δ 5.03 (d, 2H), 4.52 (d, 2H), 3.68 (s, 3H), 3.11 (s, 2H) ppm. **^{13}C NMR** (101 MHz, $CDCl_3$) δ 177.4, 171.5, 171.1, 77.9, 77.6, 52.1, 51.9, 45.5, 44.2, 39.4, 39.1 ppm. **IR** (neat, cm^{-1}) 3395, 2970, 1738, 1710, 1367, 1211. **HRMS** ESI, ($C_7H_{10}NaO_5$) $[M+Na]^+$ *calculated* 197.0420, *found* 197.0421.

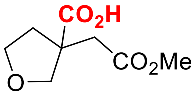

2ad

3-(2-methoxy-2-oxoethyl)tetrahydrofuran-3-carboxylic acid (2ad). Following the general procedure III using Methyl 2-(dihydro-3(2H)-furanylidene)acetate **1ad** (142 mg, 1.0 mmol), affording **2ad**(120 mg, 63.8% yield). Colourless liquid

^{1}H NMR (400 MHz, $CDCl_3$) δ 4.16 (d, 1H), 3.91 (m, 2H), 3.71 (d, 1H), 3.65 (s, 3H), 2.77 (s, 2H), 2.48 (m, 1H), 1.87 (m, 1H) ppm. **^{13}C NMR** (101 MHz, $CDCl_3$) δ 179.8, 171.7, 75.8, 68.0, 51.9, 51.1, 40.4, 36.5 ppm. **IR** (neat, cm^{-1}) 3449, 2982, 1738, 1689, 1441, 1218. **HRMS** ESI, ($C_8H_{12}NaO_5$) $[M+Na]^+$ *calculated* 211.0577, *found* 211.0578.

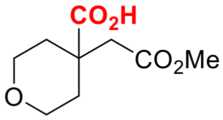

2ae

4-(2-methoxy-2-oxoethyl)tetrahydro-2H-pyran-4-carboxylic acid (2ae). Following the general procedure III using Methyl 2-(tetrahydro-4H-pyran-4-ylidene)acetate **1ae** (156 mg, 1.0 mmol), affording **2ae** (150 mg, 74.2% yield). White solid m.p. 59 °C.

^{1}H NMR (400 MHz, $CDCl_3$) δ 3.76 (m, 2H), 3.67 (m, 5H), 2.67 (s, 2H), 2.10 (m, 2H), 1.63 (m, 2H) ppm. **^{13}C NMR** (101 MHz, $CDCl_3$) δ 180.4, 170.9, 64.5, 51.8, 43.4, 42.4, 33.6 ppm. **IR** (neat, cm^{-1}) 3434, 2995, 2982, 1731, 1691, 1443, 1098. **HRMS** ESI, ($C_9H_{14}NaO_5$) $[M+Na]^+$ *calculated* 225.0733, *found* 225.0734.

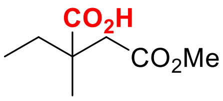

2ag

2-Ethyl-2-methyl butanedioic acid 4-methyl ester (2ag).[22] Following the general procedure III using Methyl 3-methyl-2-pentenoate (**1ag**) (128 mg, 1.0 mmol), affording **2ag** (140 mg, 80.5% yield). White solid m.p. 55 °C.

^{1}H NMR (400 MHz, $CDCl_3$) δ 10.37 (br, 1H), 3.62 (s, 3H), 2.74 (d, 1H), 2.39 (d, 1H), 1.62 (m, 2H), 1.21 (s, 3H), 0.85 (t, 3H) ppm. **^{13}C NMR** (101 MHz, $CDCl_3$) δ 182.7, 171.8, 51.6, 44.4, 42.0, 32.0, 20.9, 8.6 ppm.

Procedure for electrochemical flow reaction: In 50 mL Erlenmeyer flask, acrylate **1ag** (1 g, 7.8 mmol) was added to a solution of Et_4NI (1 g, 3.9 mmol) and TEOA (1.16 g, 7.8 mmol) in DMF (25 mL). The resulting mixture was flushed with CO_2 and kept under a positive pressure of CO_2. The mixture was electrolysed at a constant voltage of 10V through a vapourtech ion electrochem reactor containing carbon cathode and carbon anode in closed cycle with speed of 0.5 mL/min for 4 h. The crude reaction mixture was acidified by addition of HCl/H_2O (1:1, 10 mL) and extracted with diethylether (3 x 15mL). The combined organic phases were washed with brine, dried over $MgSO_4$ and filtered. The solvent was then removed under reduced pressure and the crude material was recrystalised by hexane afforing **2ag** (1.1g, 80.8%).

Deuterium Labelling Studies

Following the general procedure III but using 10 eq of D_2O instead of TEOA and starting with Methyl 3,3-dimethylacrylate affording the deuterated product **[D]2q.**

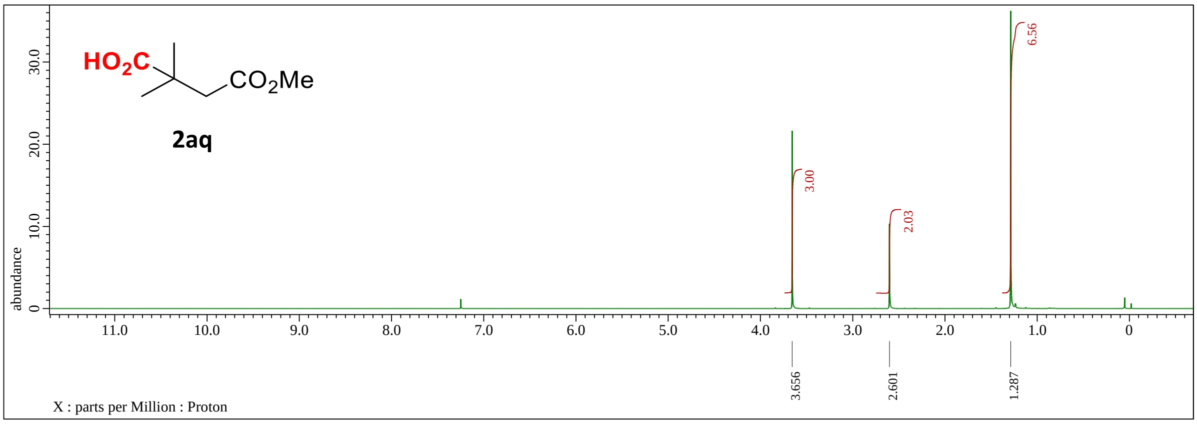
HO2C
CO2Me
2aq
abundance
3.00
2.03
6.56
3.656
2.601
1.287
X : parts per Million : Proton

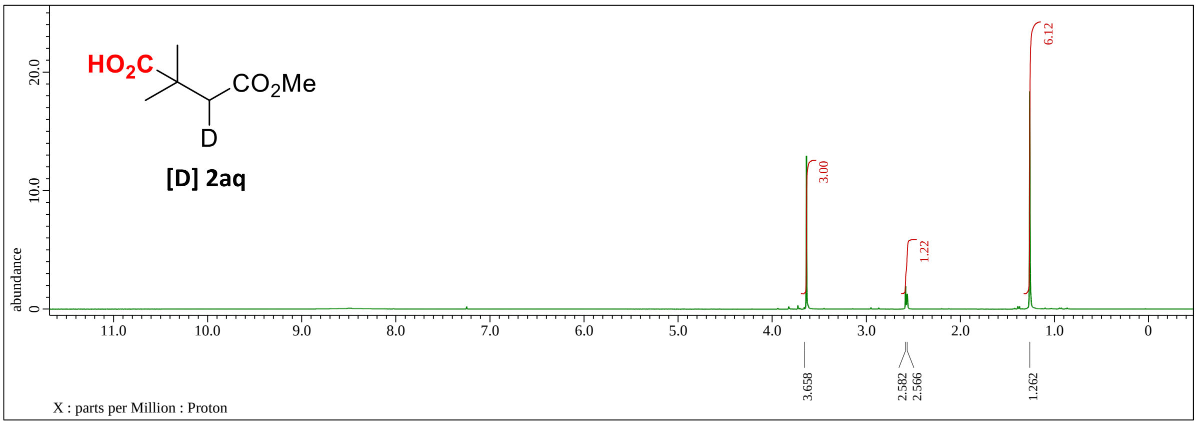
HO2C
CO2Me
D
[D] 2aq
abundance
3.00
1.22
6.12
3.658
2.582
2.566
1.262
X : parts per Million : Proton

4.0 Robustness Screen for the reaction:

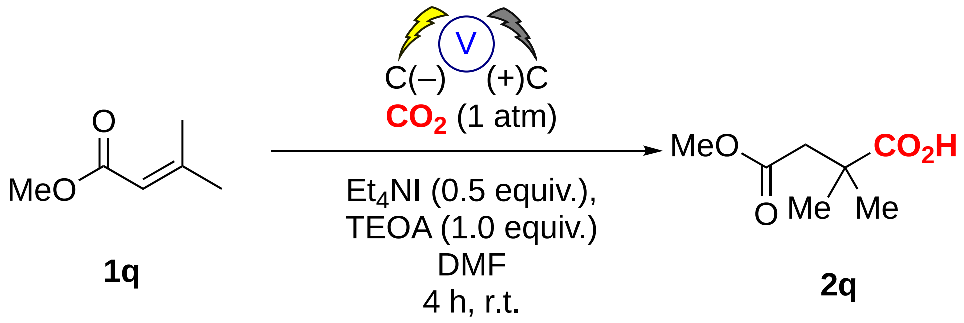

Procedure for screening

Methyl 3,3-dimethylacrylate **1q** (1.0 mmol) and additive (1.0 mmol) were added to a solution of Et_4NI (128 mg, 0.5 mmol) and TEOA (149 mg, 1.0 mmol) in DMF (5 mL). The resulting mixture was flushed with CO_2 and kept under a positive pressure of CO_2. The mixture was electrolysed at a constant voltage of 10V in a single compartment cell containing carbon cathode and carbon anode with stirring for 4h. The reaction mixture was diluted to 20 mL using chloroform and 178 mg (1.0 mmol) of Anthracene were added then analysed by GCMS.

Procedure for gas chromatography batch calibration

Additives were individually analysed by gas chromatography to determine retention times. Calibration solutions with 16 compounds (1.0 mmol of each compound in 20 mL of chloroform) were subsequently prepared, ensuring compounds with similar retention times were separated. Standard gas chromatography calibration using anthracene as a standard was undertaken, and calibration lines produced for each additive.

Calibration for reaction yield

The calibration line for determination of the yield using gas chromatography analysis is given. Calibration was undertaken using anthracene as the standard.

Column: 15 m x 0.25 mm DB-5

Method: Initial temperature 60 °C, hold 1 min, final temperature 260 °C, hold 1.35 min.

Detection: MS detection.

Retention time: 2,2-Dimethyl butanedioic acid 4-methyl ester = 5.51 min.

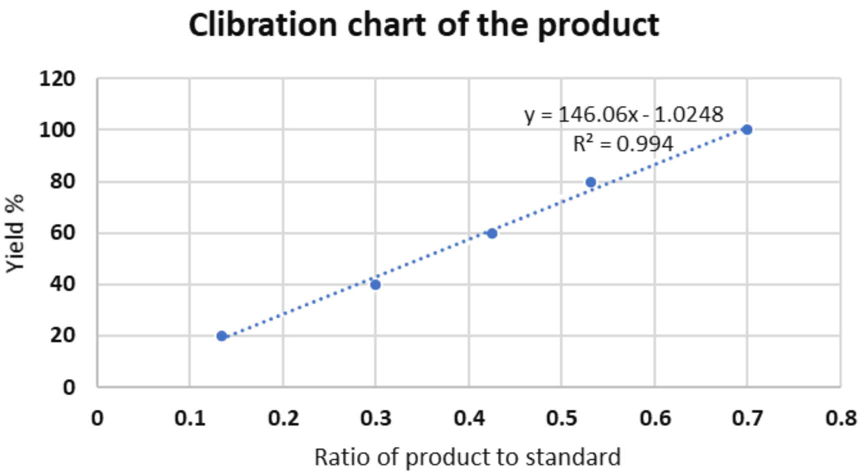

Protocol for batch screening and analysis

Two calibration solutions containing 8 additives (1.0 mmol of each additive) and standard (1.0 mmol, anthracene) in chloroform (20 mL) are prepared, and analyzed.

Example GC method, compounds, retention times and chromatograms are provided:

Column: 15 m x 0.25 mm DB-5

Method: Initial temperature 60 °C, hold 1 min, final temperature 260 °C, hold 1.35 min.

Detection: MS detection.

Retention time of standard: Anthracene = 12.79 min.

Group A		Group B	
Compound	Retention time (min)	Compound	Retention time (min)
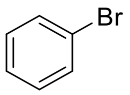	3.27	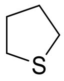	2.30
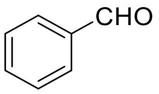	3.52	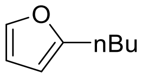	2.88
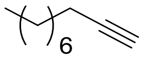	3.95	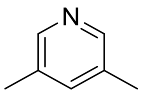	3.61
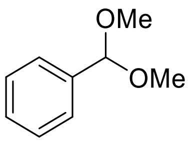	4.71	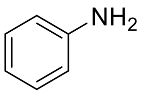	3.64
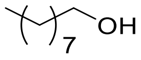	5.18	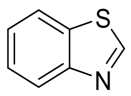	5.81
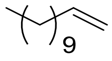	5.34	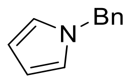	7.07

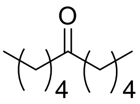	6.09	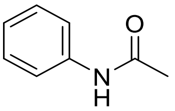	7.42
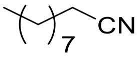	6.26	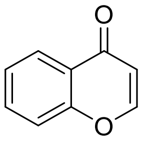	7.63

Group A Chromatogram:

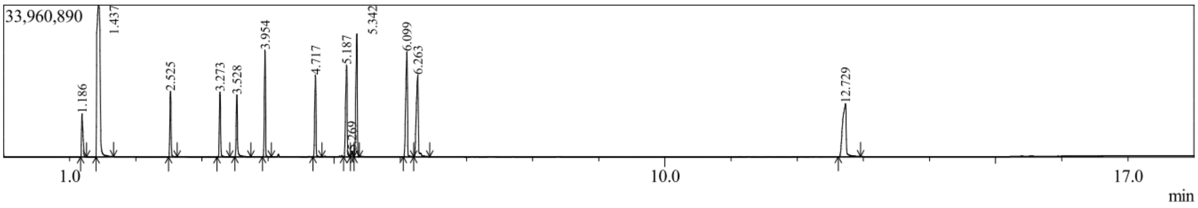

Group B Chromatogram:

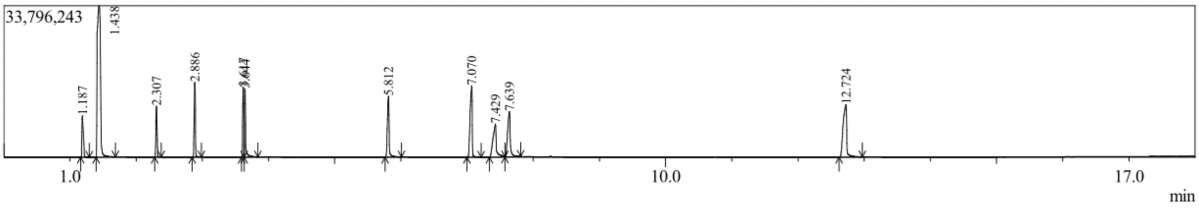

The relative integrals of the additive to the standard can be used for a semi-quantitative determination of the amount of additive present in a reaction mixture.

The standard reaction is performed and the product isolated. Calibration of the product (1.0 mmol) against the standard (1.0 mmol) is undertaken.

The standard reaction is prepared 16 times containing one additive (1.0 mmol). After the given reaction time, the standard is introduced to each reaction (1.0 mmol), and analysis by the selected method undertaken.

The yield of the reaction and the amount of additive remaining as determined by comparison with the calibration is reported in the following table:

Entry	Additive	Product Yield (%)	Additive remaining (%)	SM remaining (%)
A0	-	81.2	-	0
A1	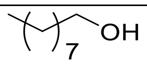	64.8	91.8	0
A2	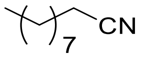	63.9	81.4	0
A3	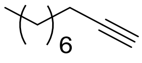	77.9	93.6	0
A4	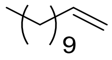	78.7	79.4	0

A5	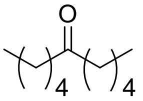	70.1	55.5	0
A6	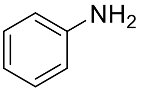	61.7	0	0
A7	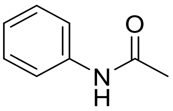	69.6	95.5	0
A8	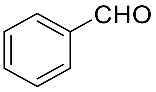	74.8	0	7.1
A9	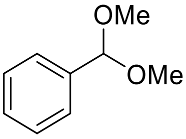	74.7	59.8	0
A10	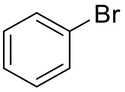	78.1	9.5	0
A11	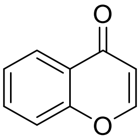	16.1	0	25.3
A12	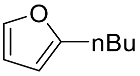	68.4	80.9	0
A13	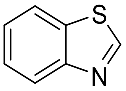	46.2	30.5	14.9
A14	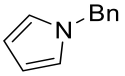	45.4	35.4	5.9
A15	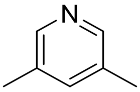	61.6	79.7	6.9
A16	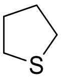	63.8	11.6	0

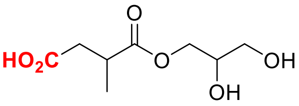

2ah

2-Methyl butanedioic acid 1-(1,2-dihydroxyprop-3-yl) ester (2ah). Following the general procedure III using Oxiranylmethyl methacrylate **1ah** (142 mg, 1.0 mmol), affording **2ah** (110 mg, 57.8% yield). Colorless oil.

^{1}H NMR (500 MHz, $CDCl_3$) δ 5.20 (br, exchanges with D_2O, 2H), 4.27-4.07 (m, 3H), 3.59 (m, 2H), 2.91 (m, 1H), 2.73 (dd, J = 6.4, 16.8 *Hz*, 1H), 2.52 (dd, J = 6.0, 16.2 *Hz*, 1H), 1.24 (d, 3H) ppm. **^{13}C NMR** (125 MHz, $CDCl_3$) δ 176.7, 175.4, 69.6, 65.5, 45.5, 37.4, 35.7, 17.0 ppm. **IR** (neat, cm^{-1}) 3542, 3450, 2990, 2895, 1735, 1704, 1421, 1260, 1163. **HRMS** ESI, ($C_8H_{14}NaO_6$) $[M+Na]^+$ *calculated* 229.0683, *found* 229.0684.

5.0 Current Screening for the reaction:

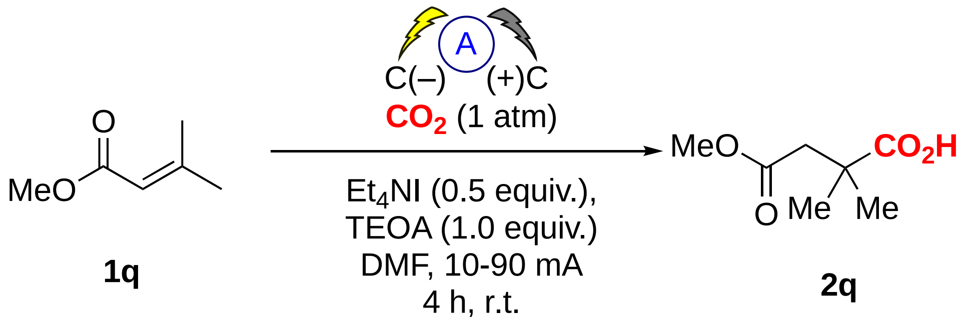

1q **2q**

Procedure for screening

Methyl 3,3-dimethylacrylate **1q** (1.0 mmol) was added to a solution of Et_4NI (128 mg, 0.5 mmol) and TEOA (149 mg, 1.0 mmol) in DMF (5 mL). The resulting mixture was flushed with CO_2 and kept under a positive pressure of CO_2. The mixture was electrolysed at different interval current in a single compartment cell containing carbon cathode and carbon anode with stirring for 4h. The reaction mixture was diluted to 20 mL using chloroform and 178 mg (1.0 mmol) of anthracene internal standard was added then analysed by GCMS.

Current (mA)	Product GCMS Yield (%)	S.M Remaining (%)	Current Efficiency (%)
10	0	100	N/A
30	31.8	51.1	14
60	68.9	0	15
90	74	0	11

6.0 Analysis of CO_2 reduction by GC-MS Headspace analysis

Reaction Using Dimethylacetamide (DMA):

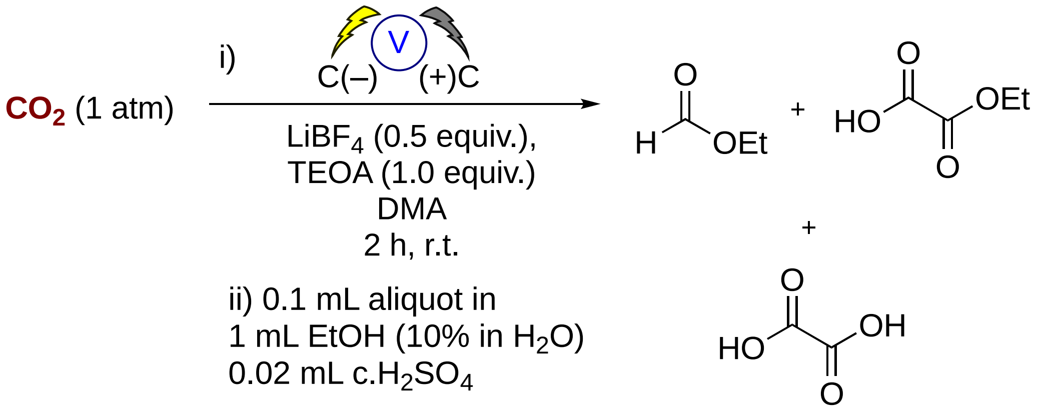

A solution of $LiBF_4$ (47 mg, 0.5 mmol) and TEOA (149 mg, 1.0 mmol) in DMA (5 mL). was flushed with CO_2 and kept under a positive pressure of CO_2. The mixture was electrolysed at a constant voltage of 10V in a single compartment cell containing carbon cathode and carbon anode with stirring for 2h. Utilising the method of Sokoro *et. al.*[26] an aliquot (0.1 mL) was placed into a GC-MS vial and diluted to 1.0 mL with aq. EtOH (10% v/v), c.H_2SO_4 (0.02 mL) was added and the vial top screwed into place. The vial was

placed in an oven for 10 minutes at 60 ºC along with a gas tight syringe. The vial was then allowed to cool for 5 minutes and 1.0 mL of the head space in the vial drawn into the syringe and immediately injected into the GC-MS.

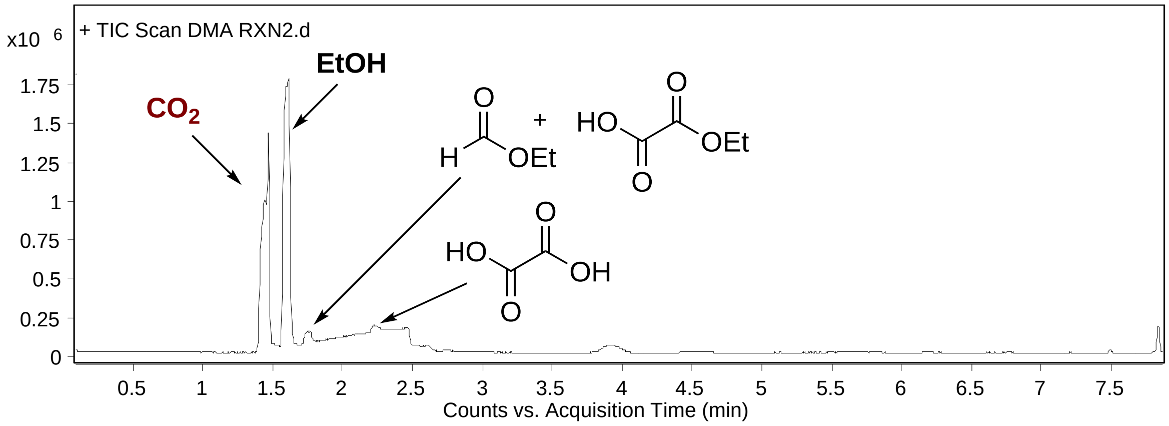

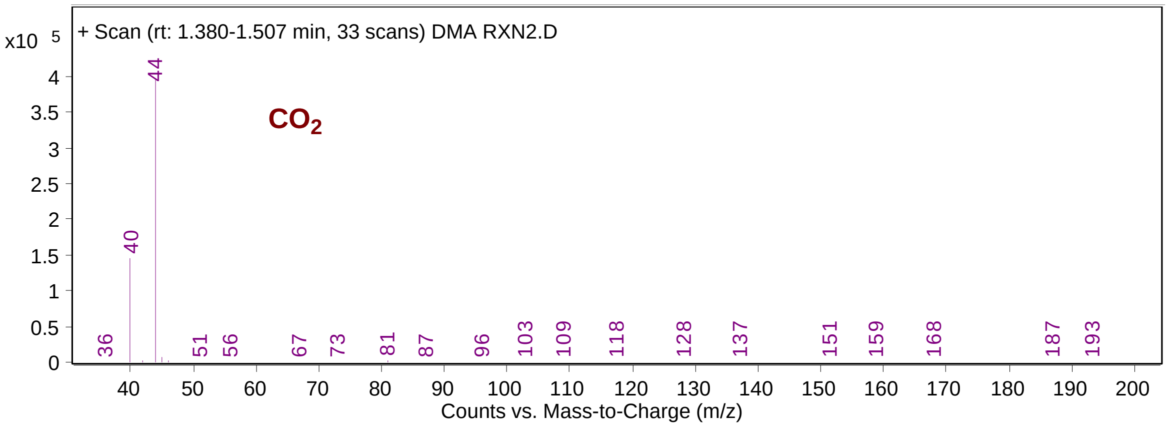

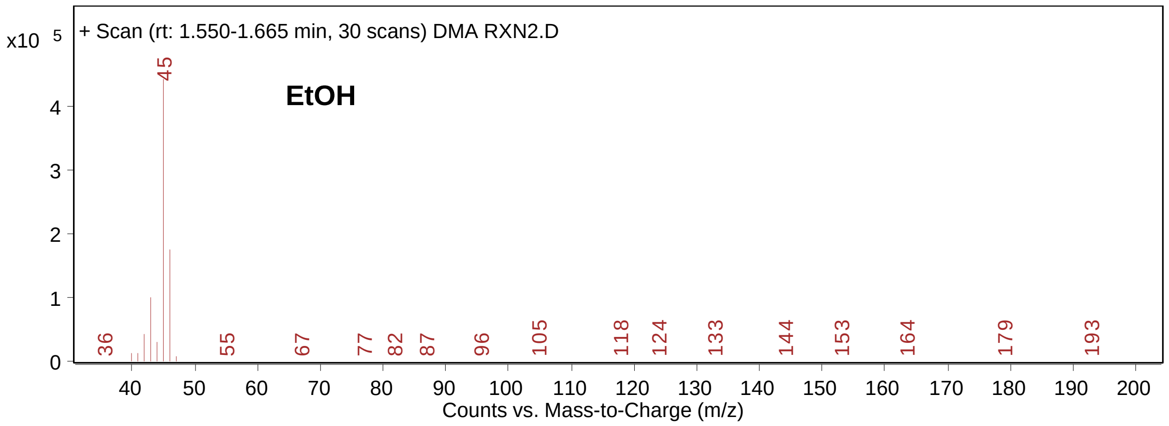

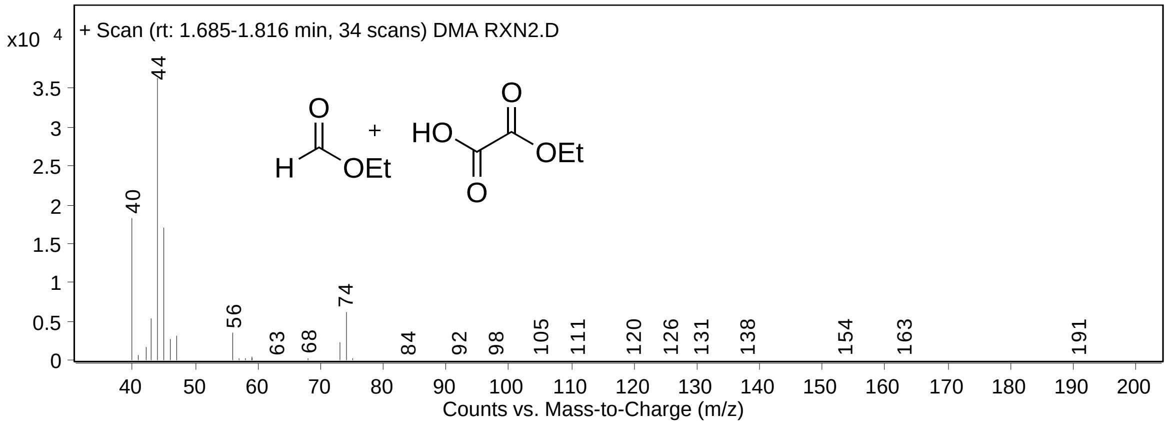

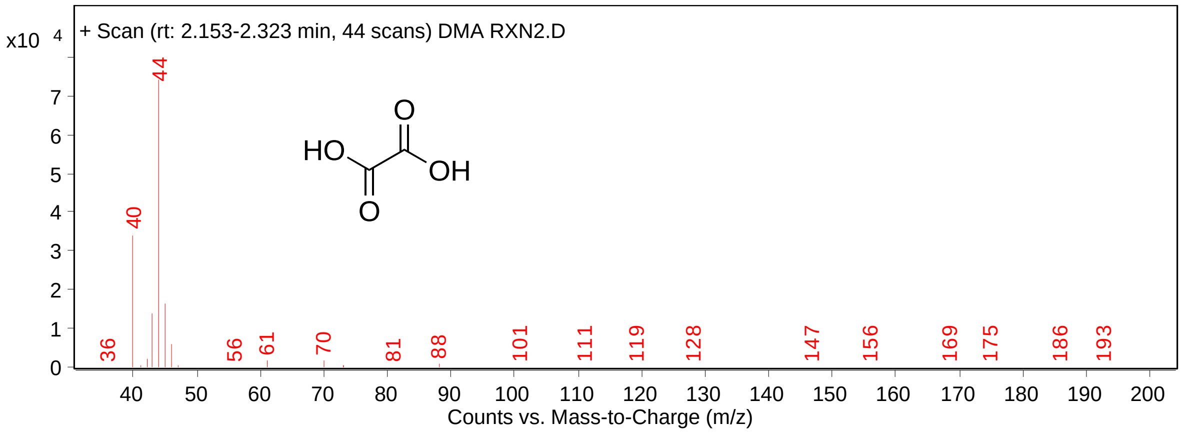

Blank DMA GC-MS Spectra:

DMA (0.1 mL) was placed into a GC-MS vial and diluted to 1.0 mL with EtOH (10% v/v), c.H_2SO_4 (0.02 mL) was added and the vial top screwed into place. The vial was placed in an oven for 10 minutes at 60 ºC along with a gas tight syringe. The vial was then allowed to cool for 5 minutes and 1.0 mL of the head space in the vial drawn into the syringe and immediately injected into the GC-MS.

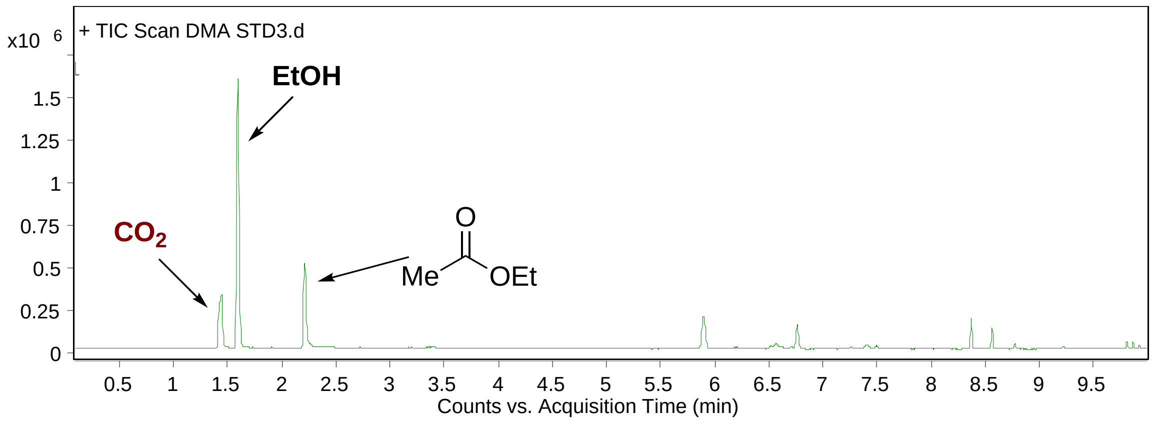

x10 6
+ TIC Scan DMA STD3.d
EtOH
CO2
O
Me
OEt
Counts vs. Acquisition Time (min)

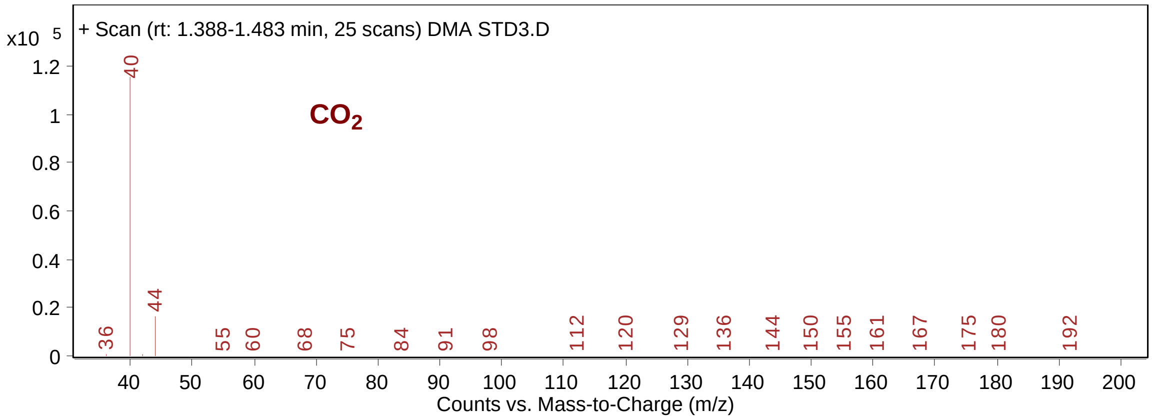

x10 5
+ Scan (rt: 1.388-1.483 min, 25 scans) DMA STD3.D
CO2
Counts vs. Mass-to-Charge (m/z)

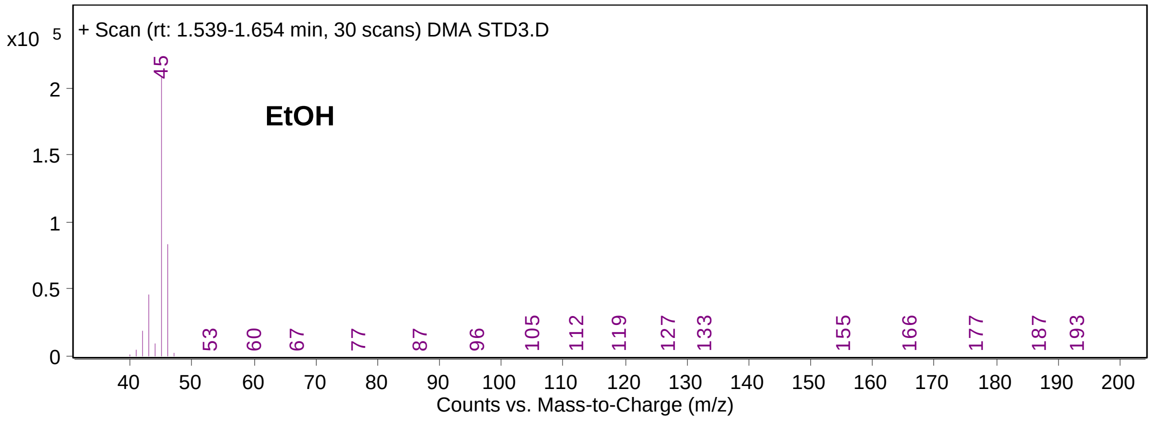

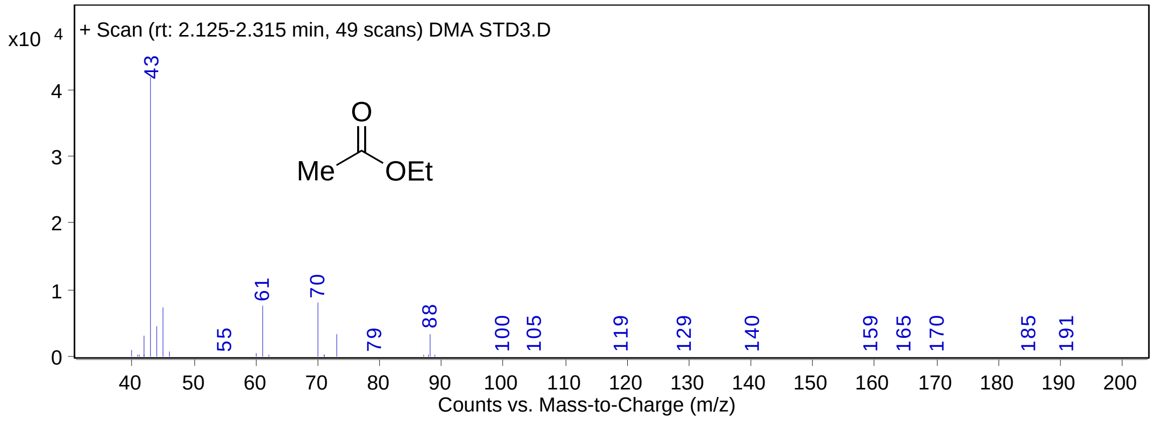

Reaction Using Dimethylformamide (DMF):

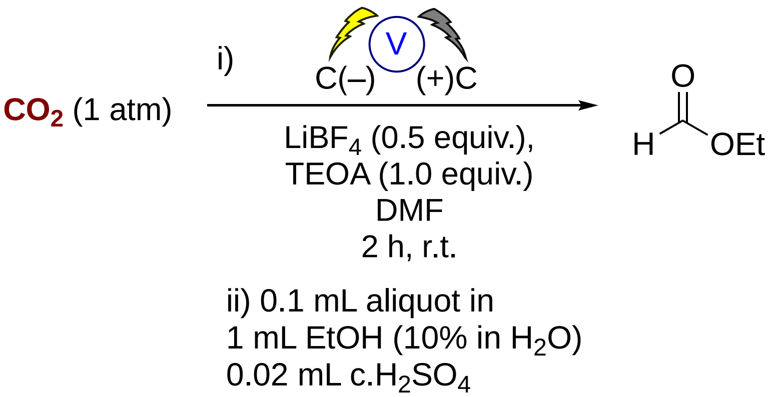

A solution of $LiBF_4$ (47 mg, 0.5 mmol) and TEOA (149 mg, 1.0 mmol) in DMF (5 mL). was flushed with CO_2 and kept under a positive pressure of CO_2. The mixture was electrolysed at a constant voltage of 10V in a single compartment cell containing carbon cathode and carbon anode with stirring for 2h. An aliquot (0.1 mL) was placed into a GC-MS vial and diluted to 1.0 mL with aq. EtOH (10% v/v), c.H_2SO_4 (0.02 mL) was added and the vial top screwed into place. The vial was placed in an oven for 10 minutes at 60 ºC along with a gas tight syringe. The vial was then allowed to cool for 5 minutes and 1.0 mL of the head space in the vial drawn into the syringe and immediately injected into the GC-MS.

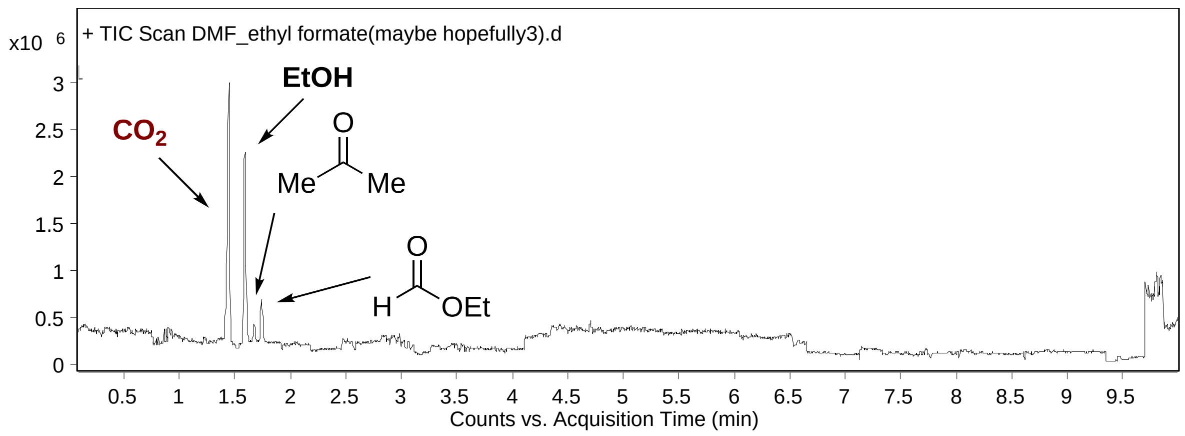

+ TIC Scan DMF_ethyl formate(maybe hopefully3).d
x10 6
EtOH
CO2
Me
O
Me
H
O
OEt
Counts vs. Acquisition Time (min)

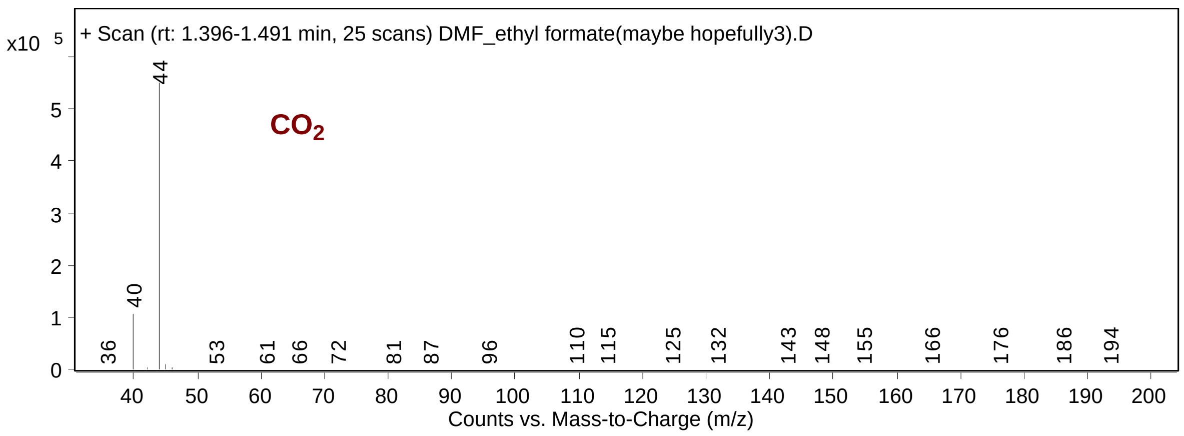

+ Scan (rt: 1.396-1.491 min, 25 scans) DMF_ethyl formate(maybe hopefully3).D
x10 5
CO2
Counts vs. Mass-to-Charge (m/z)

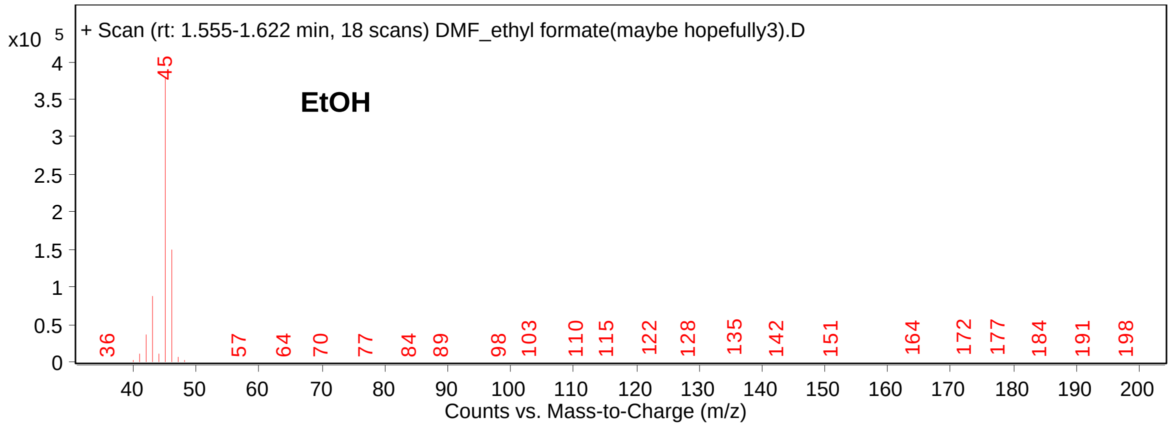

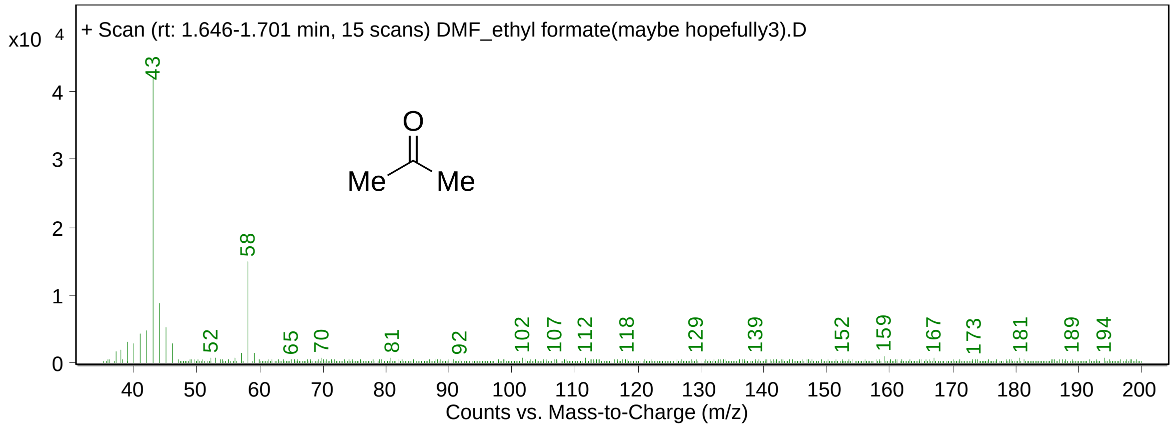

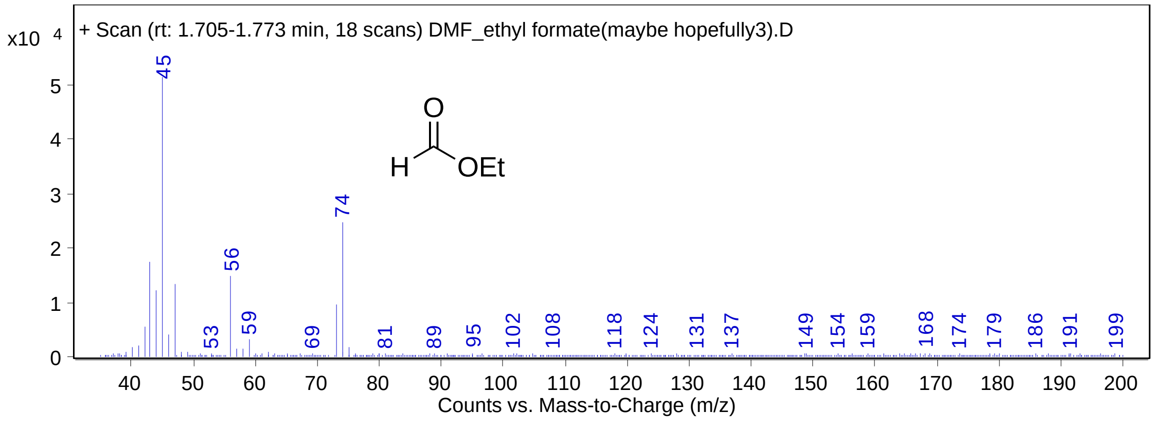

Blank DMF GC-MS Spectra

DMF (0.1 mL) was placed into a GC-MS vial and diluted to 1.0 mL with EtOH (10% v/v), c.H_2SO_4 (0.02 mL) was added and the vial top screwed into place. The vial was placed in an oven for 10 minutes at 60 ^{o}C along with a gas tight syringe. The vial was then allowed to cool for 5 minutes and 1.0 mL of the head space in the vial drawn into the syringe and immediately injected into the GC-MS.

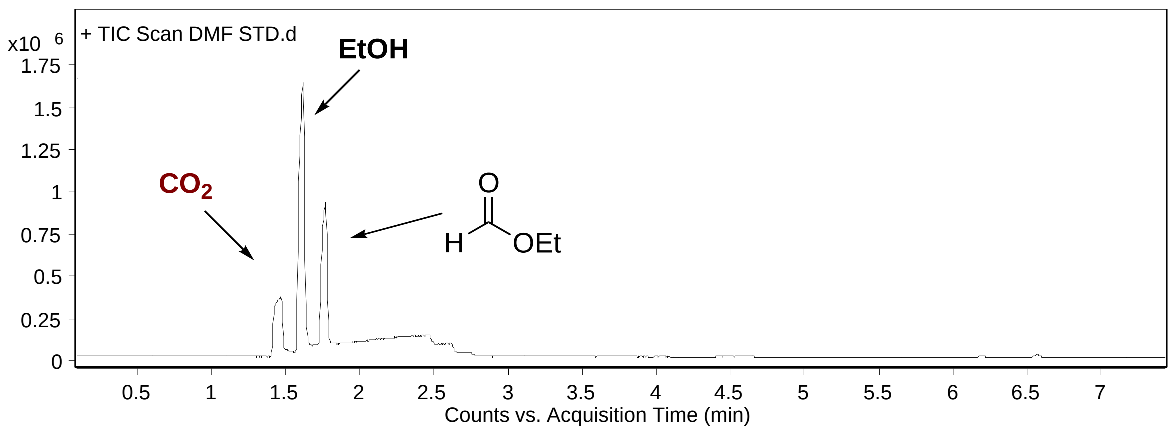
+ TIC Scan DMF STD.d
x10 6
EtOH
CO_2
O
H
OEt
Counts vs. Acquisition Time (min)

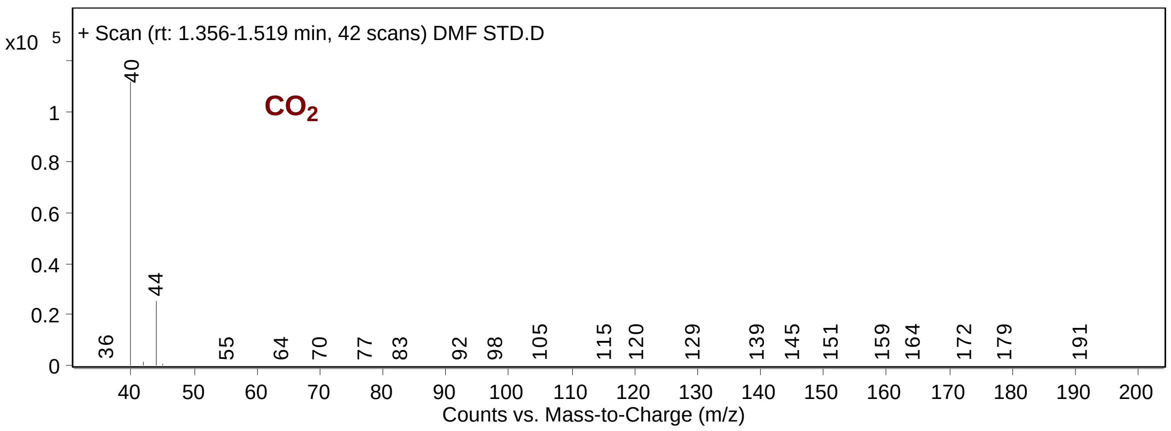
+ Scan (rt: 1.356-1.519 min, 42 scans) DMF STD.D
x10 5
CO_2
36 40 44 55 64 70 77 83 92 98 105 115 120 129 139 145 151 159 164 172 179 191
Counts vs. Mass-to-Charge (m/z)

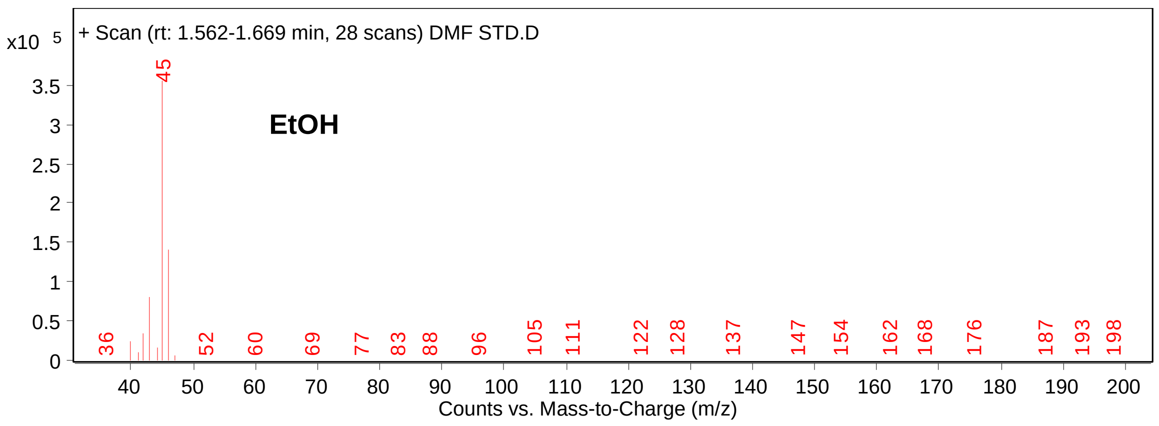
+ Scan (rt: 1.562-1.669 min, 28 scans) DMF STD.D
x10 5
EtOH
36 45 52 60 69 77 83 88 96 105 111 122 128 137 147 154 162 168 176 187 193 198
Counts vs. Mass-to-Charge (m/z)

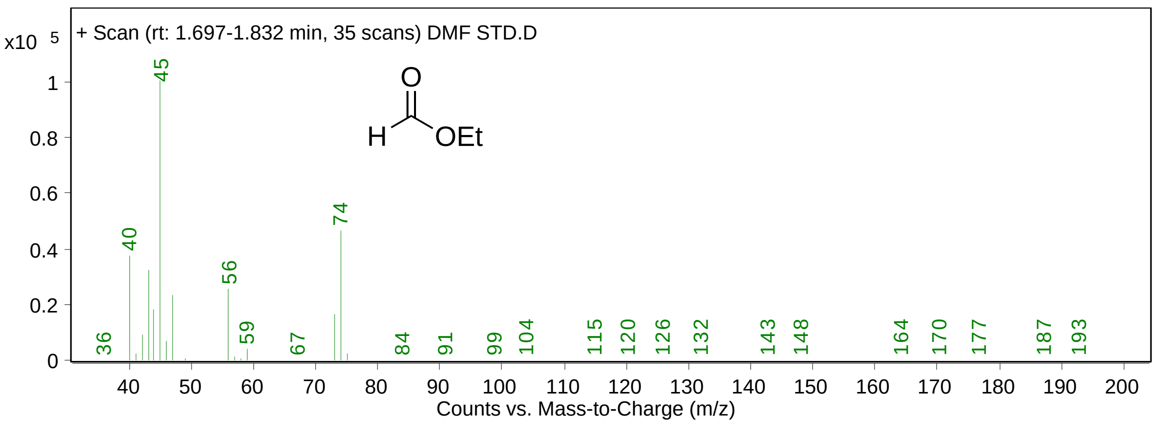
+ Scan (rt: 1.697-1.832 min, 35 scans) DMF STD.D
x10 5
O
H
OEt
36 40 45 56 59 67 74 84 91 99 104 115 120 126 132 143 148 164 170 177 187 193
Counts vs. Mass-to-Charge (m/z)

7.0 References:

1- G. Singh, M. L. Purkayastha, H. Ila, H. Junjappa, *J. Chem. Soc., Perkin Trans.* 1 **1985,** *7*, 1289–1294.

2- K. F. Chin, X. Ye, Y. Li, R. Lee, A. M. Kabylda, D. Leow, X. Zhang, E. C. Xia Ang, C. Tan, *ACS Catalysis* **2020,** 10, 2684–2691.

3- T. Kapferer, R. Brückner, *Eur. J. Org. Chem.* **2006,** *9*, 2119–2133.

4- A. Meden, D. Knez, M. Jukic, X. Brazzolotto, M. Grsic, A. Pislar, A. Zahirovic, J. Kos, F. Nachon, J. Svete, S. Gobec, U. Groselj, *Chem. Commun.* **2019,** *55*, 3765–3768.

5- G. Kang, D. Romo *ACS Catal.* **2021**, *11*, 1309-1315

6- Y. Wang, N. Wang, J. Zhao, M. Sun, H. You, F. Fang, Z. Liu, *ACS Cat.* **2020,** *10*, 6603–6612.

7- P. W. Smith, A. W. J. Cooper, R. Bell, I. J. M. Beresford, P. M. Gore, A. B. McElroy, J. M. Pritchard, V. Saez, N. R. Taylor, *J. Med. Chem.* **1995,** *38*, 3772–3779.

8- X. Yang, W. Kong, J. Gao, L. Cheng, N. Li, M. Li, H. Li, J. Fan, J. Gao, Q. Ouyang, J. Xie, *Chem. Commun.* **2019,** 55, 12707–12710.

9- C. Xue, X. Chen, X. He, J. Roderick, R. L. Corbett, B. Ghavimi, R. Liu, M. B. Covington, M. Qian, M. D. Ribadeneira, K. Vaddi, J. Trzaskos, R. C. Newton, J.J.-W. Duan, C. P. Decicco, *Bioorg. Med. Chem. Lett.* **2004,** 14, 4453–4459.

10-A. R. White, R. A. Kozlowski, S. Tsai, C. D. Vanderwal, *Angew. Chem.* **2017,** *129*, 10661–10665.

11-J. Duan, B. W. King, C. Decicco, T.P. Maduskuie Jr., M. E. Voss, *PCT Int. Appl.* **2001,** WO 2001070734.

12-M. E. Kuehne, W. G. Bornmann, I. Marko, Y. Qin, K. L. LeBoulluec, D. A. Frasier, F. Xu, T. Mulamba, C. L. Ensinger, L. S. Borman, A. E. Huot, C. Exon, F. T. Bizzarro, J. B. Cheung, S. L. Bane, *Org. Biomol. Chem.* **2003,** *1*, 2120–2136.

13-S. G. Alcock, J. E. Baldwin, R. Bohlmann, L. M. Harwood, J. I. Seeman, *J. Org. Chem.* **1985,** *50*, 3526–3535.

14-J. Sedelmeier, S. V. Ley, I. R. Baxendale, M. Baumann, *Org. Lett.* **2010,** *12*, 3618−3621.

15-M. Ostermeier, B. Brunner, C. Korff, G. Helmchen, *Eur. J. Org. Chem.* **2003,** 3453–3459.

16-K. Achiwa, P. A. Chaloner, D. Parker, *J. Organomet. Chem.* **1981,** *218*, 249–260.

17-L. Zetta, G. Gatti, *Tetrahedron* **1972,** *28*, 3773–3779.

18-S. Singh, M. Verma, K. N. Singh, *Synth. Commun.* **2004,** *34*, 4471–4475.

19-Wang, Y.; Ren, W.; Li, J.; Wang, H.; Shi, Y. *Org. Lett.* **2014,** 16, 5960–5963.

20-D. B. Hansen, M. L. Starr, N. Tolstoy, M. A. Joullie, *Tetrahedron-Asymmetry* **2005,** 16, 3623–3627.

21-Casimir, J. R.; Ettouati, L.; Paris, J. *Lett. Pept. Sci.* **1998,** 5, 13–18.

22-Norman, M. H.; Rigdon, G. C.; Navas, F., III; Cooper, B. R. *J. Med. Chem.* **1994,** 37, 2552–2563.

23-Hussain, S.A.M.T.; Ollis, W.D.; Smith, C. Stoddart, J.F. *J. Chem. Soc., Perkin I* **1974,** 1480–1483.

24-Smith, R. G. *PCT Int. Appl.* **1997,** WO 9741878.

25-Hajicek, J.; Holubek, J.; Trojanek, *J. Coll. Czech. Chem. Commun.* **1982,** 47, 2749–2762.

26-A. Sokoro, D. Lehotay, J. Eichhorst, R. Treble, *J Anal Toxicol* **2007***, 31,* 342–346.

8.0 ^1H and ^{13}C NMR Spectra of the alkenes

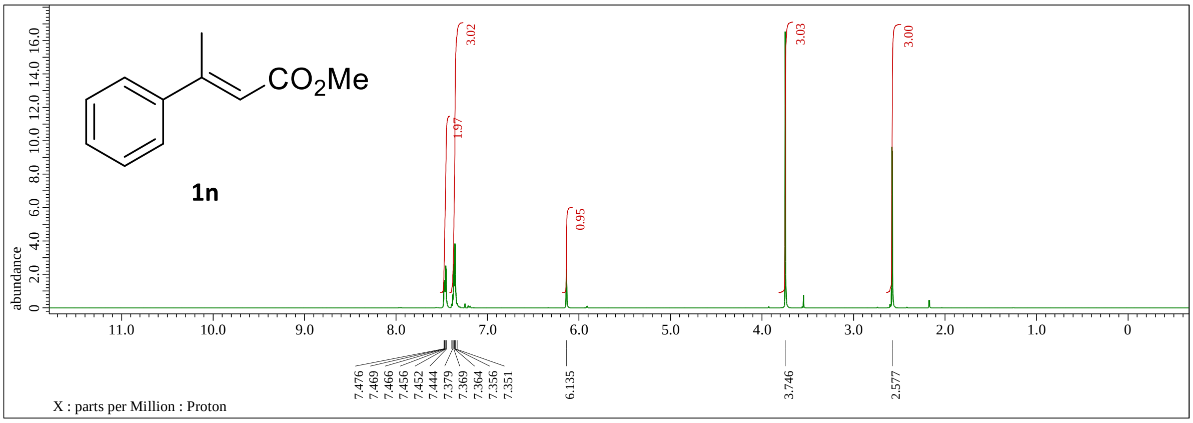

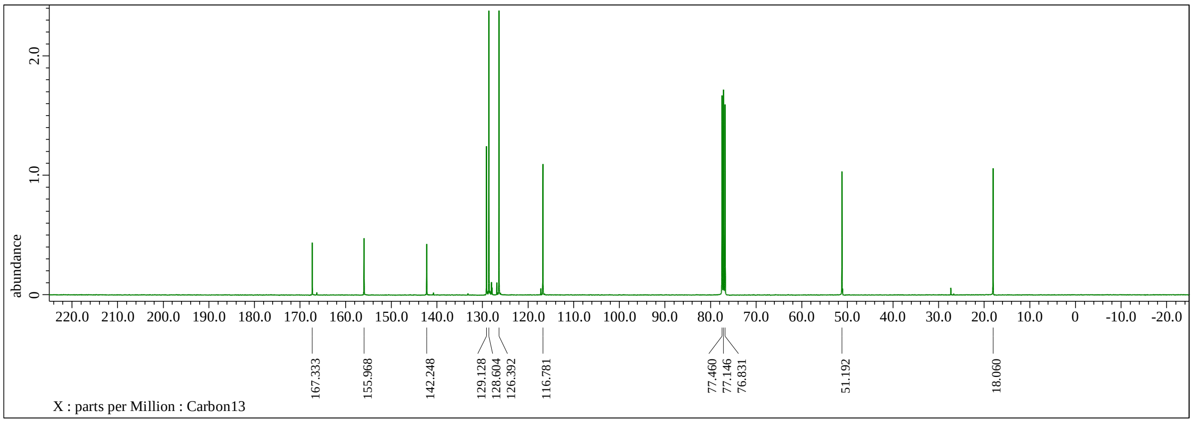

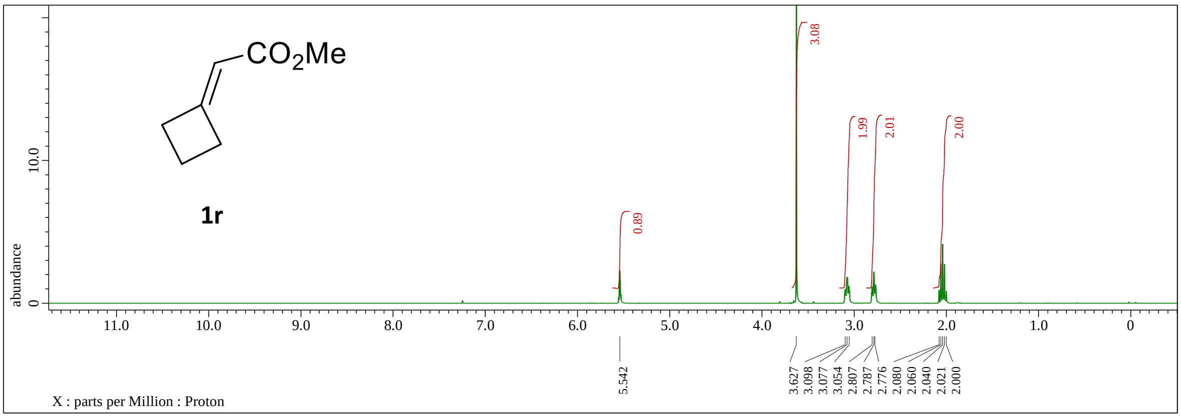

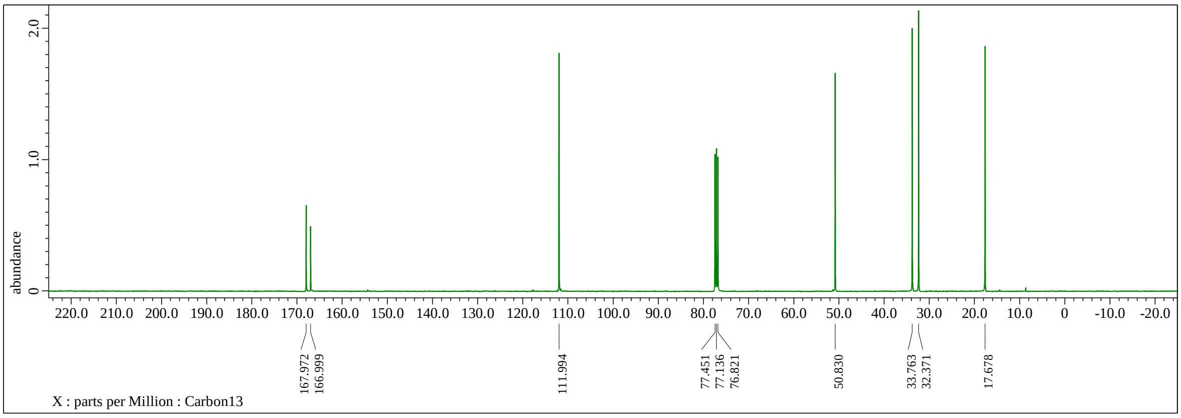

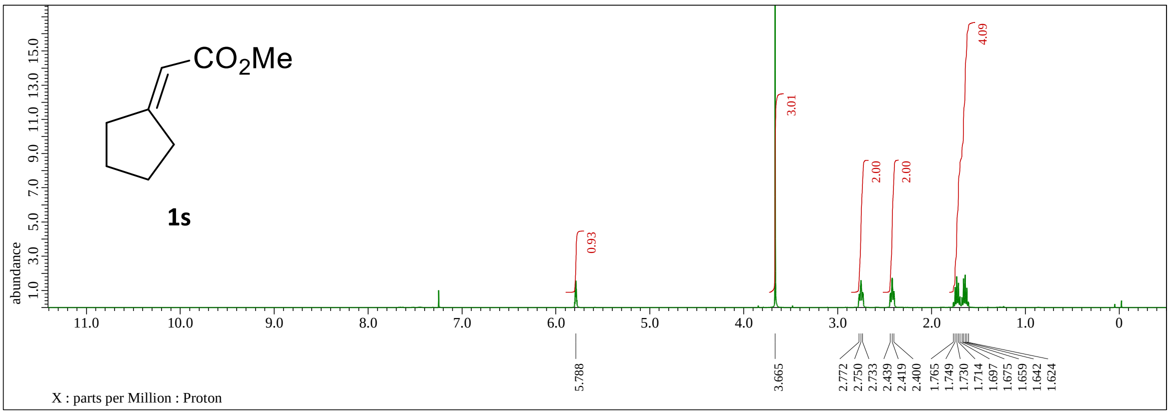
CO2Me
1s
abundance
0.93
3.01
2.00
2.00
4.09
5.788
3.665
2.772 2.750 2.733 2.439 2.419 2.400 1.765 1.749 1.730 1.714 1.697 1.675 1.659 1.642 1.624
X : parts per Million : Proton

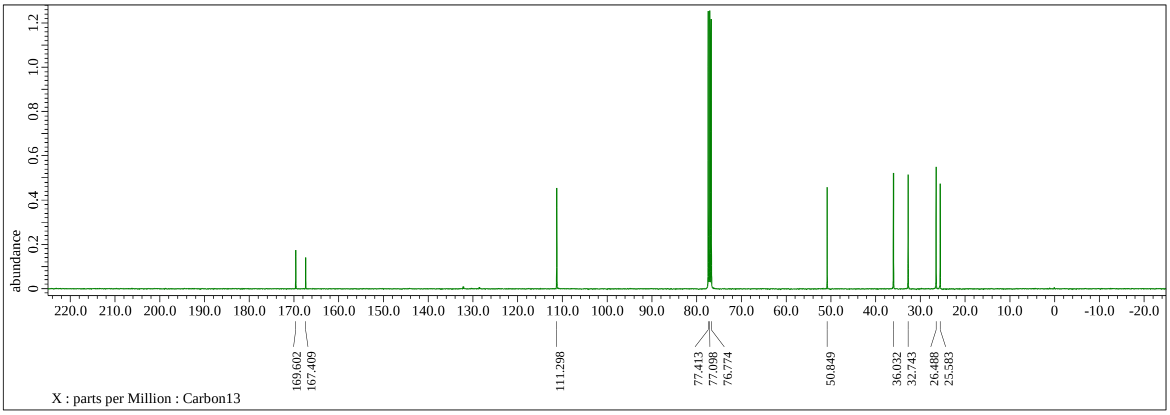
abundance
169.602 167.409
111.298
77.413 77.098 76.774
50.849
36.032 32.743
26.488 25.583
X : parts per Million : Carbon13

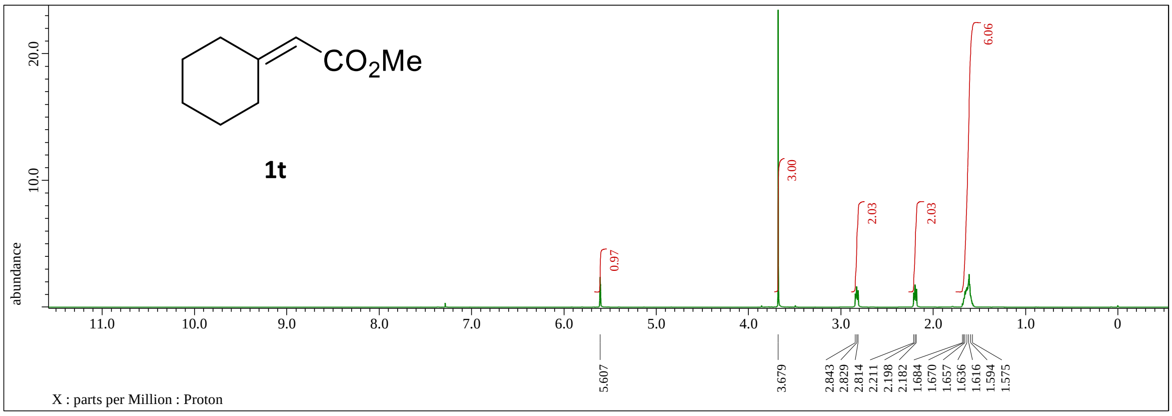
CO2Me
1t
abundance
0.97
3.00
2.03
2.03
6.06
5.607
3.679
2.843 2.829 2.814 2.211 2.198 2.182 1.684 1.670 1.657 1.636 1.616 1.594 1.575
X : parts per Million : Proton

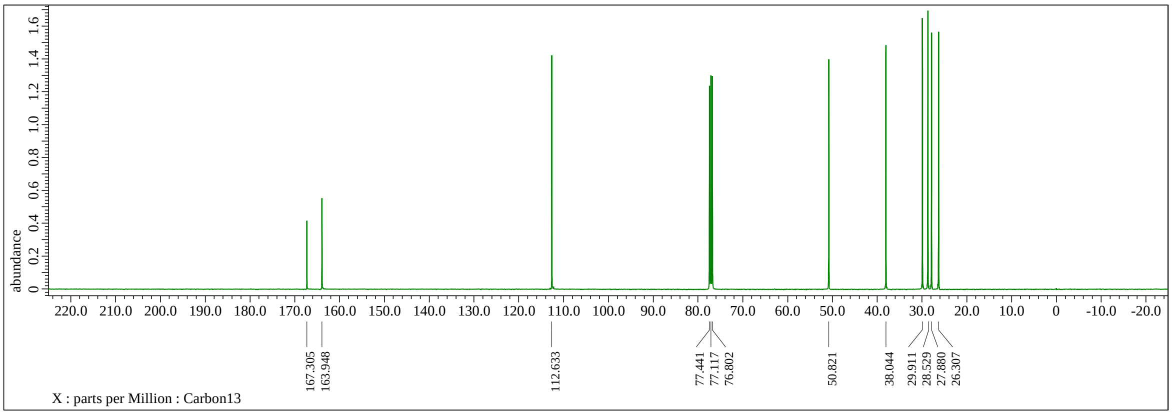
abundance
167.305 163.948
112.633
77.441 77.117 76.802
50.821
38.044
29.911 28.529 27.880 26.307
X : parts per Million : Carbon13

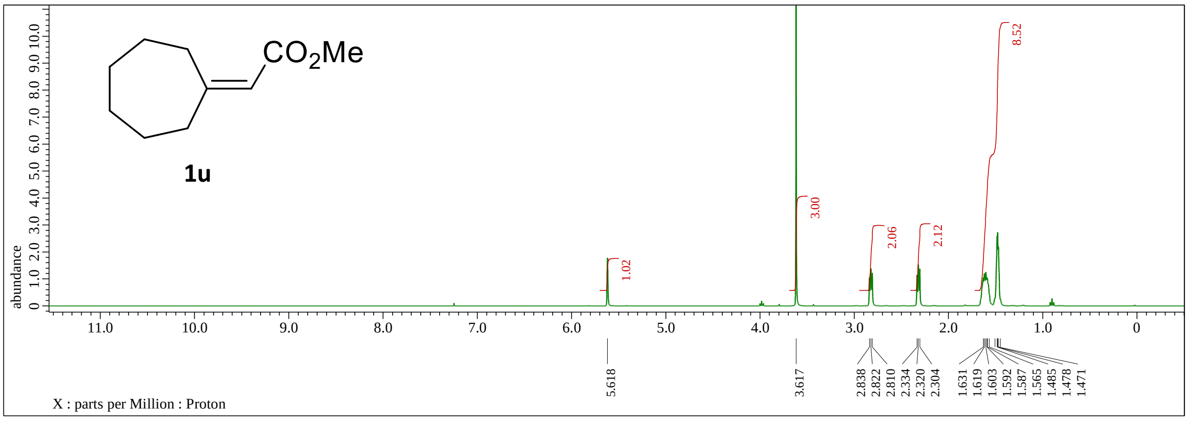

CO_2Me
1u
abundance
X : parts per Million : Proton
1.02
3.00
2.06
2.12
8.52
5.618
3.617
2.838 2.822 2.810 2.334 2.320 2.304
1.631 1.619 1.603 1.592 1.587 1.565 1.485 1.478 1.471

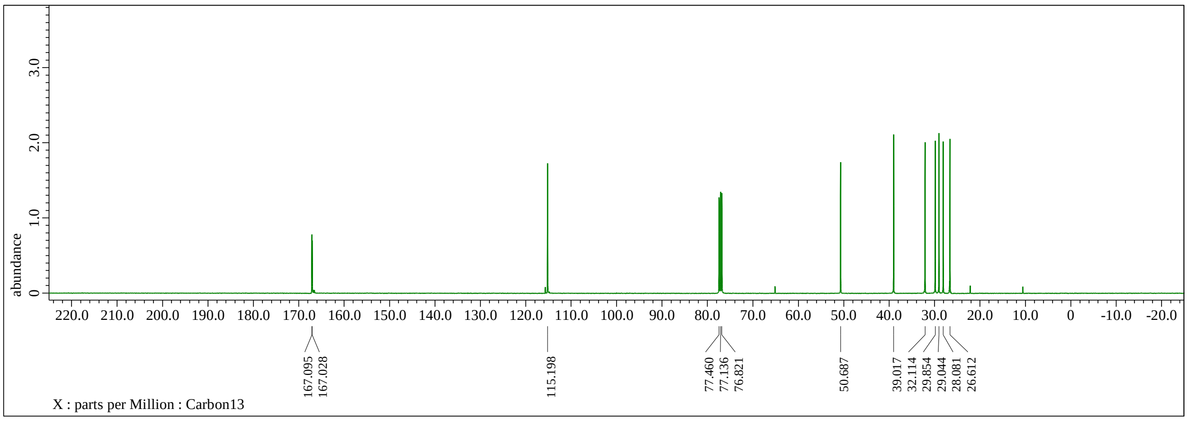

abundance
X : parts per Million : Carbon13
167.095 167.028
115.198
77.460 77.136 76.821
50.687
39.017 32.114 29.854 29.044 28.081 26.612

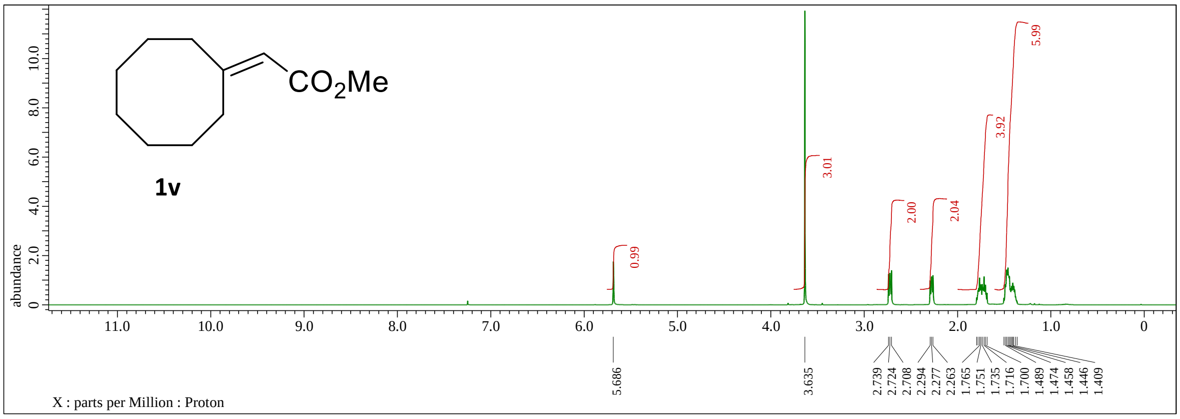

CO_2Me
1v
abundance
X : parts per Million : Proton
0.99
3.01
2.00
2.04
3.92
5.99
5.686
3.635
2.739 2.724 2.708 2.294 2.277 2.263
1.765 1.751 1.735 1.716 1.700 1.489 1.474 1.458 1.446 1.409

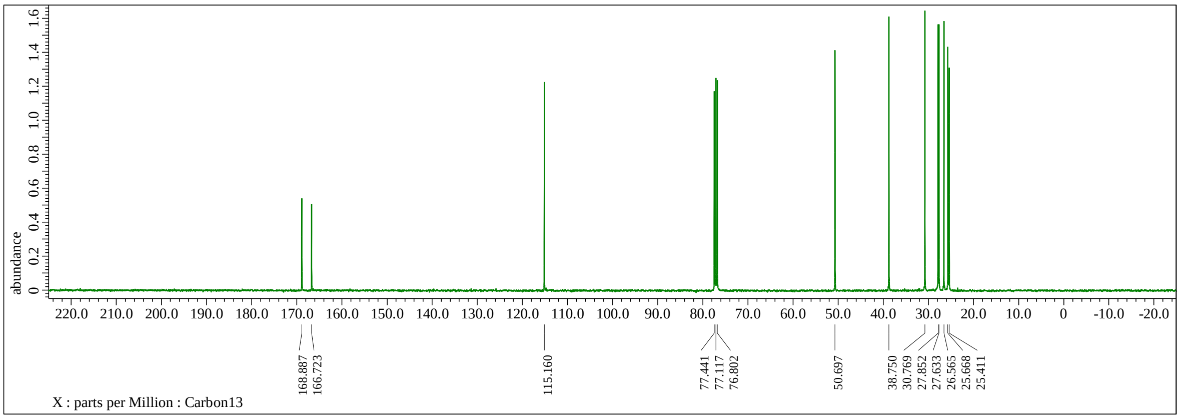

abundance
X : parts per Million : Carbon13
168.887 166.723
115.160
77.441 77.117 76.802
50.697
38.750 30.769 27.852 27.633 26.565 25.668 25.411

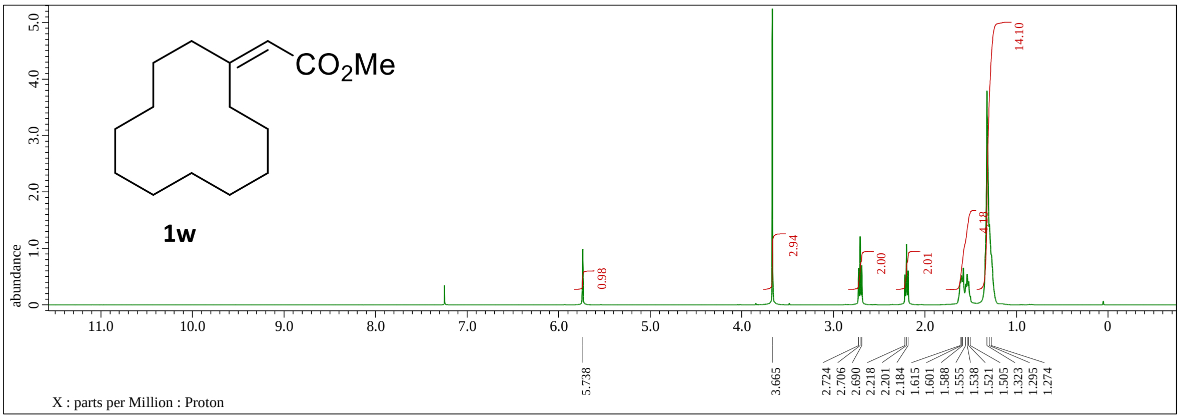
CO2Me
1w
abundance
0.98
2.94
2.00
2.01
4.18
14.10
5.738
3.665
2.724 2.706 2.690 2.218 2.201 2.184 1.615 1.601 1.588 1.555 1.538 1.521 1.505 1.323 1.295 1.274
X : parts per Million : Proton

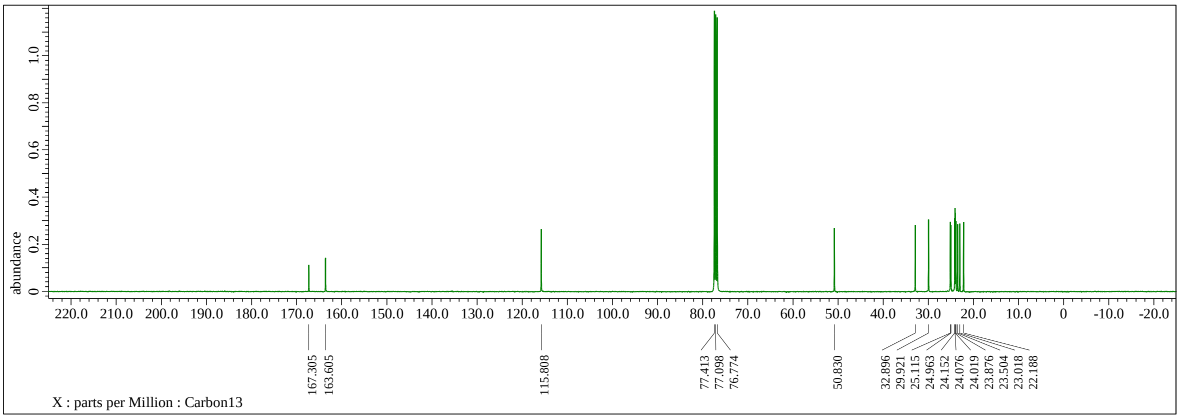
abundance
167.305 163.605
115.808
77.413 77.098 76.774
50.830
32.896 29.921 25.115 24.963 24.152 24.076 24.019 23.876 23.504 23.018 22.188
X : parts per Million : Carbon13

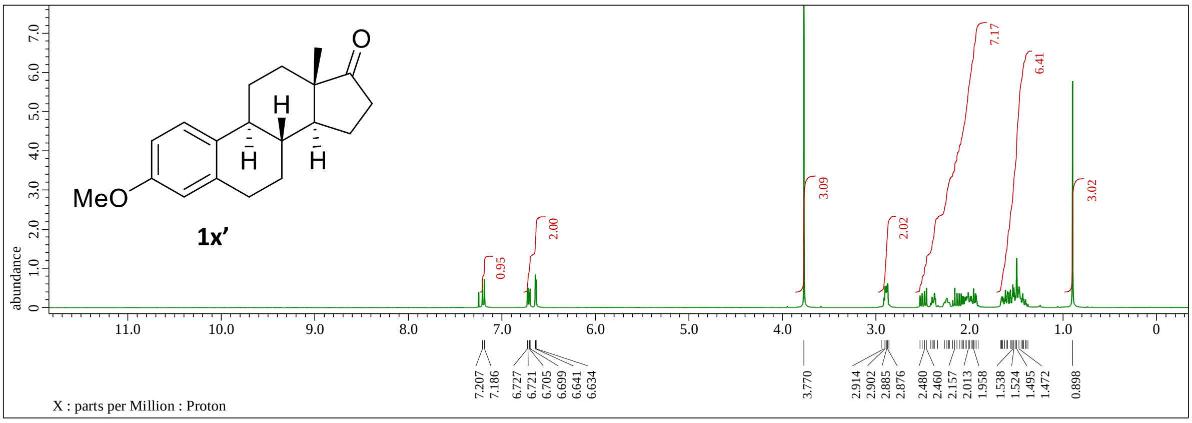
O
H
H
H
MeO
1x'
abundance
0.95
2.00
3.09
2.02
7.17
6.41
3.02
7.207 7.186
6.727 6.721 6.705 6.699 6.641 6.634
3.770
2.914 2.902 2.885 2.876
2.480 2.460 2.157 2.013 1.958
1.538 1.524 1.495 1.472
0.898
X : parts per Million : Proton

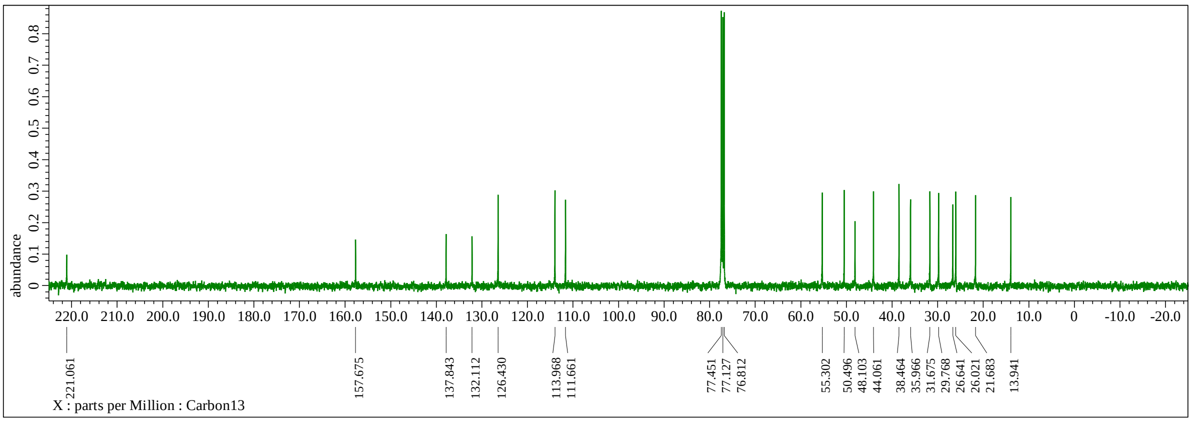
abundance
221.061
157.675
137.843 132.112 126.430
113.968 111.661
77.451 77.127 76.812
55.302 50.496 48.103 44.061 38.464 35.966 31.675 29.768 26.641 26.021 21.683 13.941
X : parts per Million : Carbon13

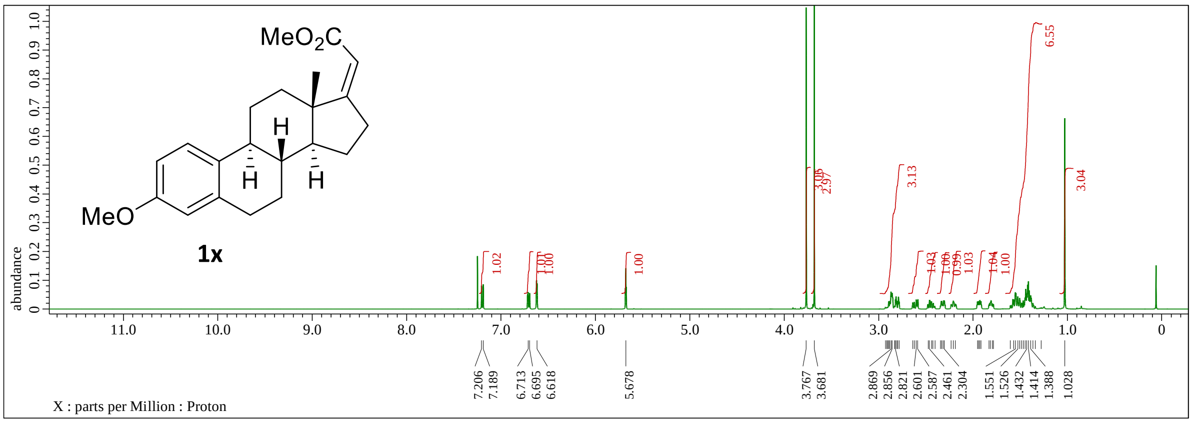
MeO$_2$C
MeO
1x
abundance
X : parts per Million : Proton
7.206 7.189 6.713 6.695 6.618 5.678 3.767 3.681 2.869 2.856 2.821 2.601 2.587 2.461 2.304 1.551 1.526 1.432 1.414 1.388 1.028

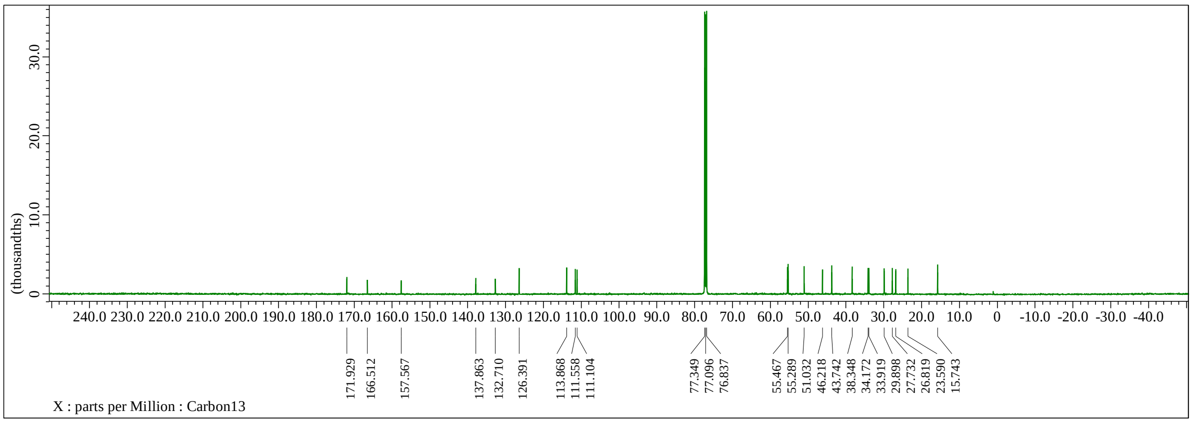
(thousandths)
X : parts per Million : Carbon13
171.929 166.512 157.567 137.863 132.710 126.391 113.868 111.558 111.104 77.349 77.096 76.837 55.467 55.289 51.032 46.218 43.742 38.348 34.172 33.919 29.898 27.732 26.819 23.590 15.743

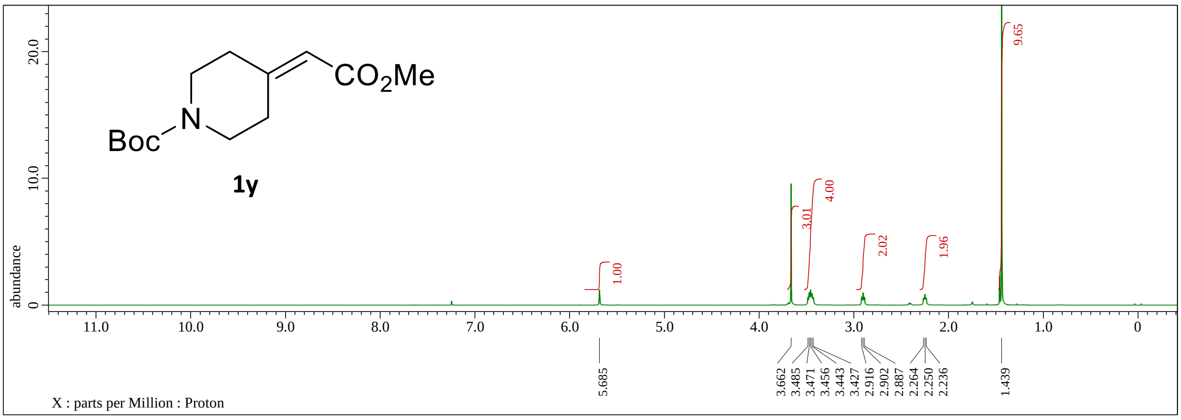
CO$_2$Me
Boc
N
1y
abundance
X : parts per Million : Proton
5.685 3.662 3.485 3.471 3.456 3.443 3.427 2.916 2.902 2.887 2.264 2.250 2.236 1.439

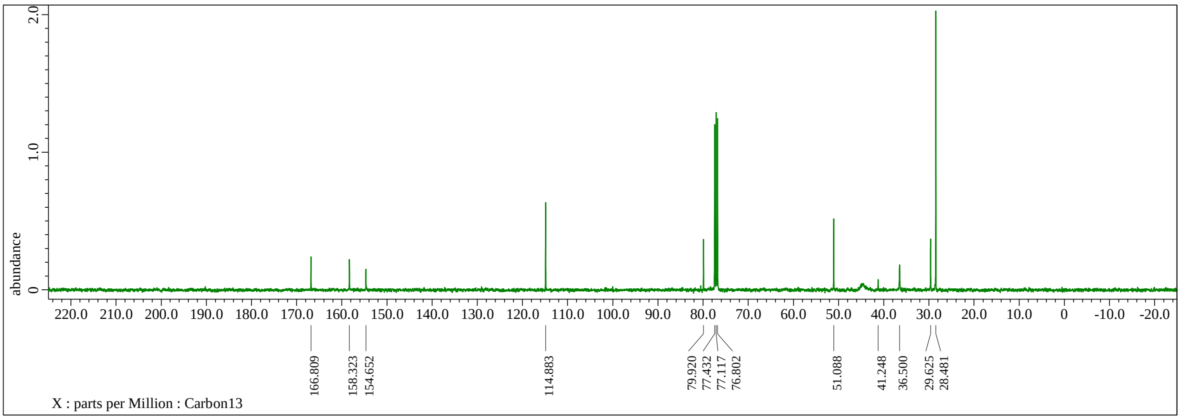
abundance
X : parts per Million : Carbon13
166.809 158.323 154.652 114.883 79.920 77.432 77.117 76.802 51.088 41.248 36.500 29.625 28.481

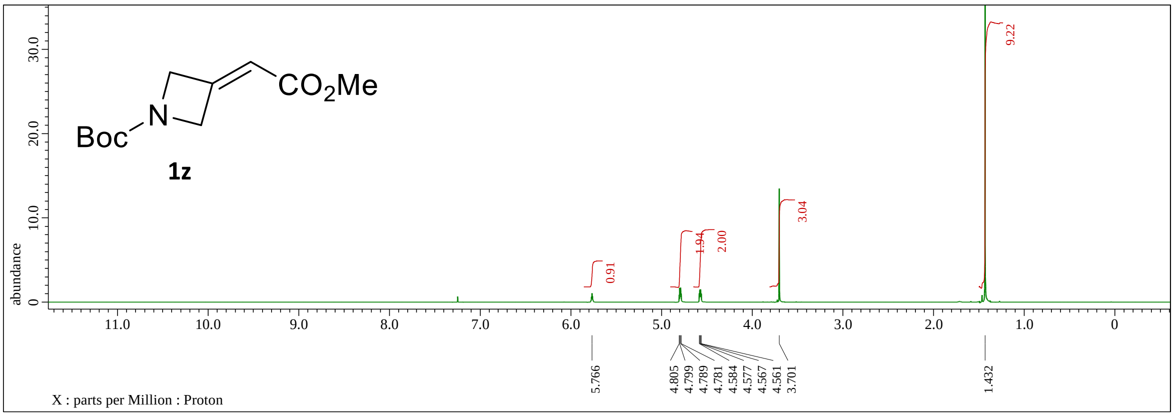
CO2Me
Boc
N
1z
abundance
X : parts per Million : Proton
0.91
1.94
2.00
3.04
9.22
5.766
4.805
4.799
4.789
4.781
4.584
4.577
4.567
4.561
3.701
1.432

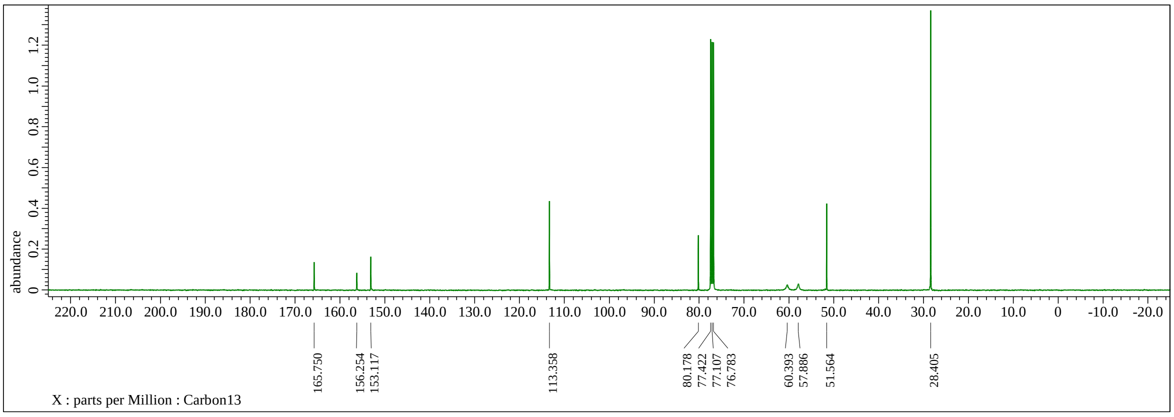
abundance
X : parts per Million : Carbon13
165.750
156.254
153.117
113.358
80.178
77.422
77.107
76.783
60.393
57.886
51.564
28.405

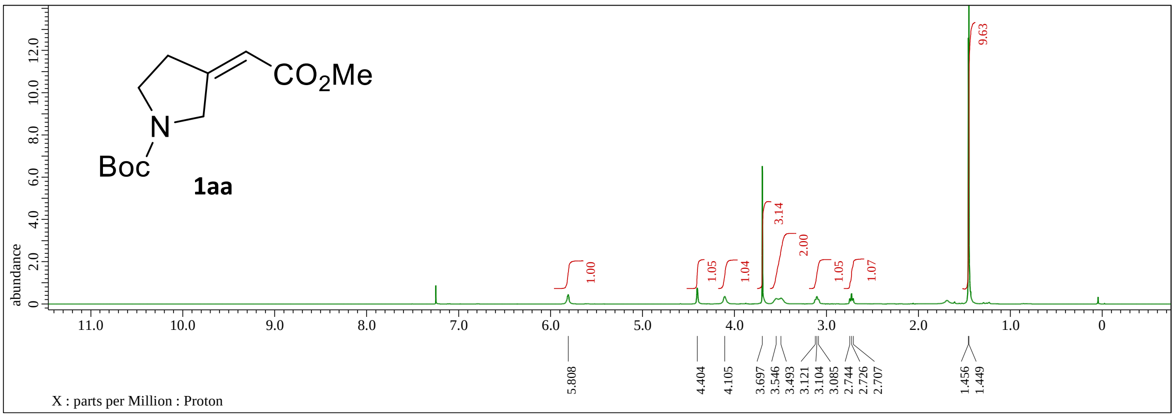
CO2Me
N
Boc
1aa
abundance
X : parts per Million : Proton
1.00
1.05
1.04
3.14
2.00
1.05
1.07
9.63
5.808
4.404
4.105
3.697
3.546
3.493
3.121
3.104
3.085
2.744
2.726
2.707
1.456
1.449

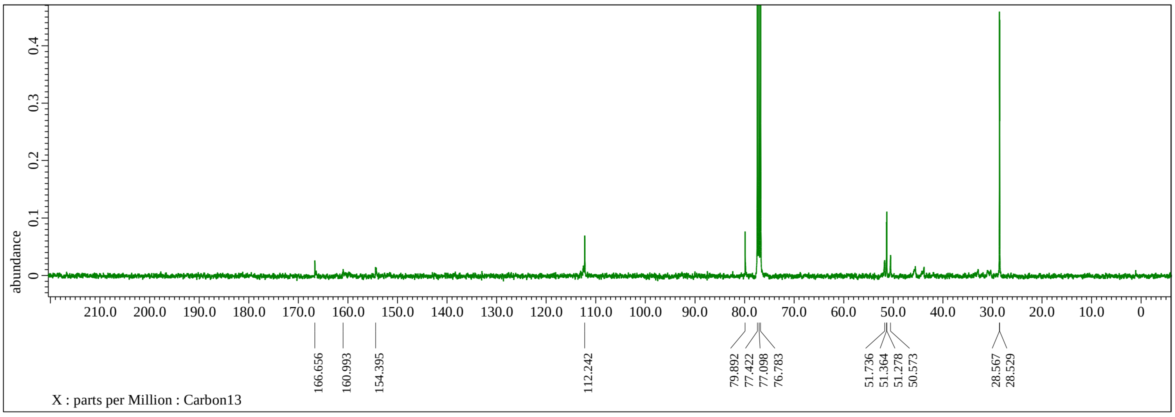
abundance
X : parts per Million : Carbon13
166.656
160.993
154.395
112.242
79.892
77.422
77.098
76.783
51.736
51.364
51.278
50.573
28.567
28.529

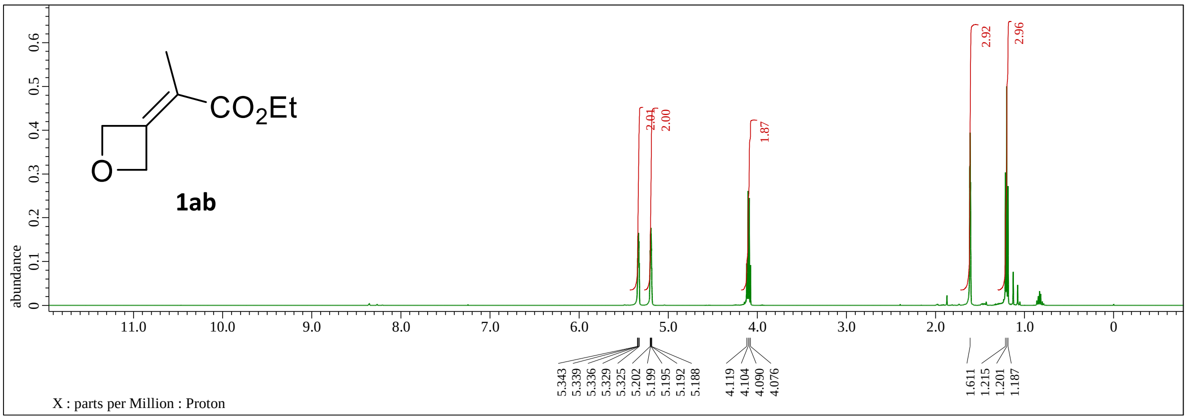
CO2Et
1ab
abundance
2.01
2.00
1.87
2.92
2.96
5.343 5.339 5.336 5.329 5.325 5.202 5.199 5.195 5.192 5.188
4.119 4.104 4.090 4.076
1.611 1.215 1.201 1.187
X : parts per Million : Proton

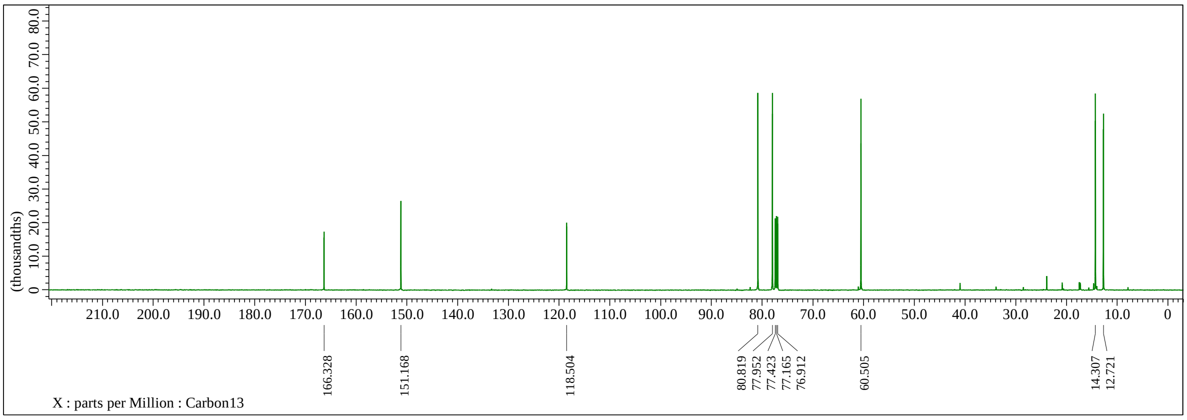
(thousandths)
166.328
151.168
118.504
80.819 77.952 77.423 77.165 76.912
60.505
14.307 12.721
X : parts per Million : Carbon13

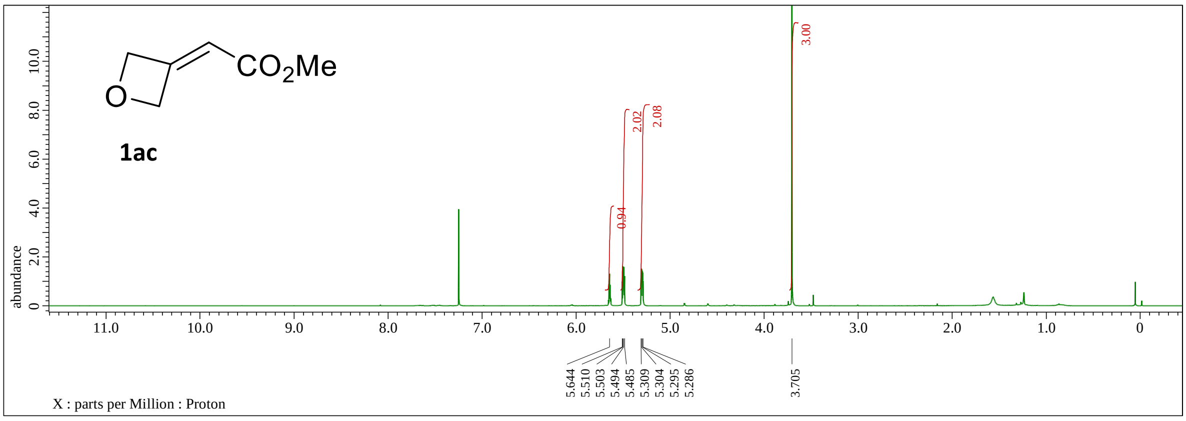
CO2Me
1ac
abundance
0.94
2.02
2.08
3.00
5.644 5.510 5.503 5.494 5.485 5.309 5.304 5.295 5.286
3.705
X : parts per Million : Proton

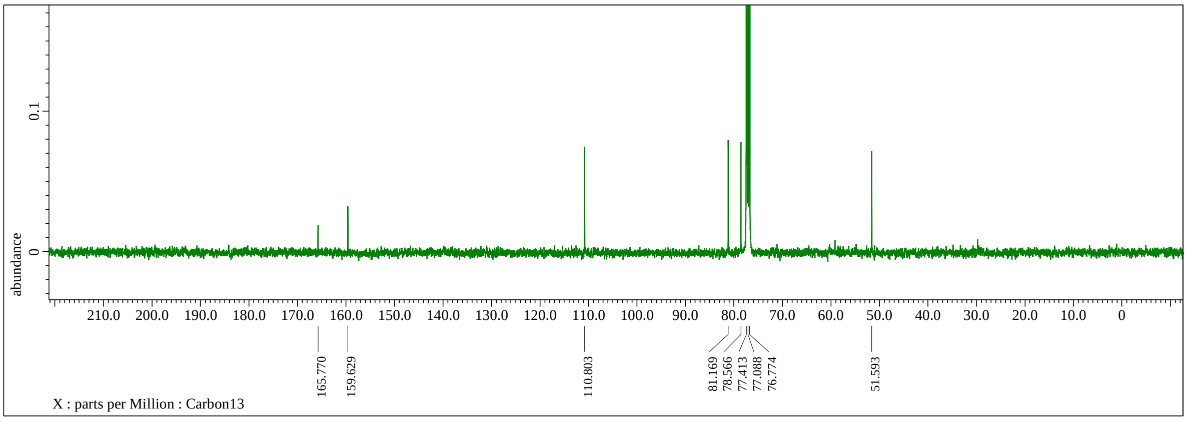
abundance
165.770
159.629
110.803
81.169 78.566 77.413 77.088 76.774
51.593
X : parts per Million : Carbon13

1ad

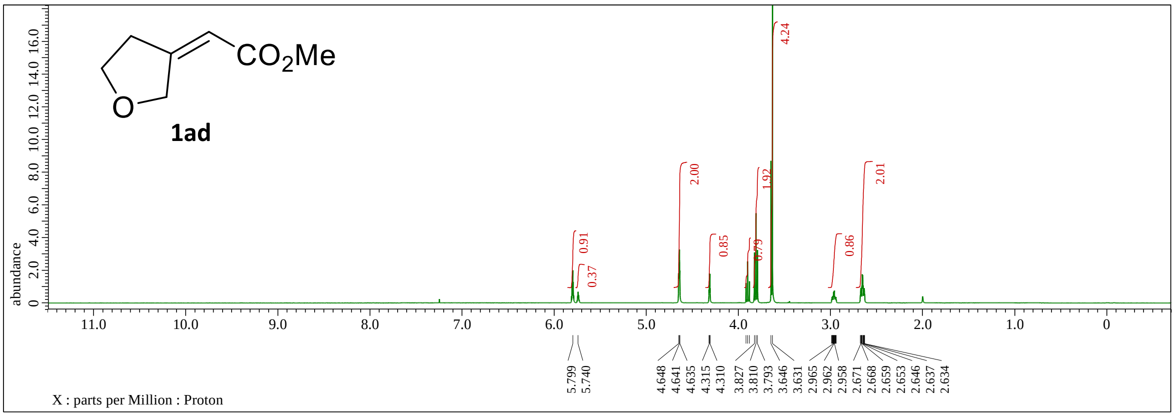

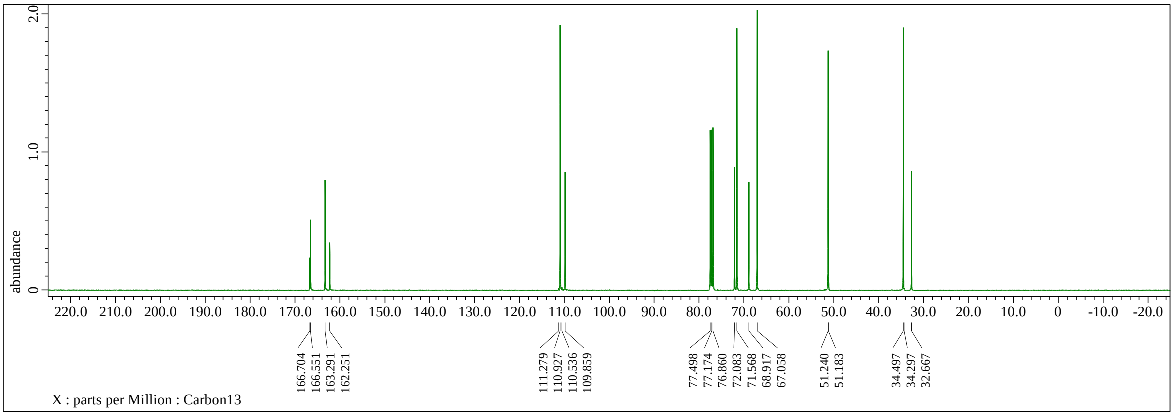

1ae

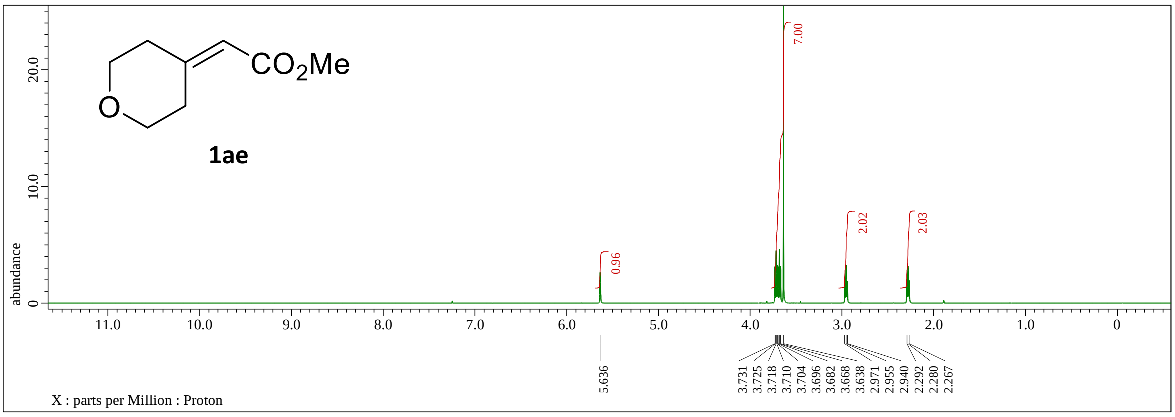

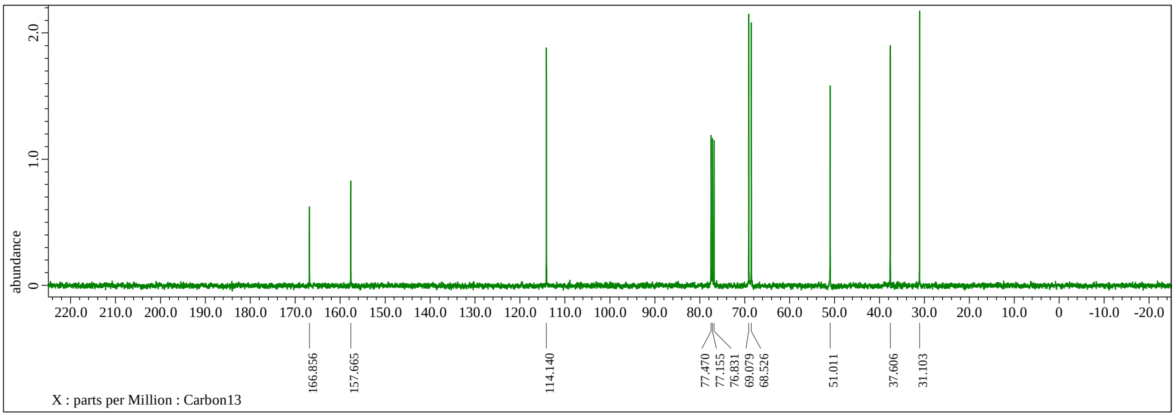

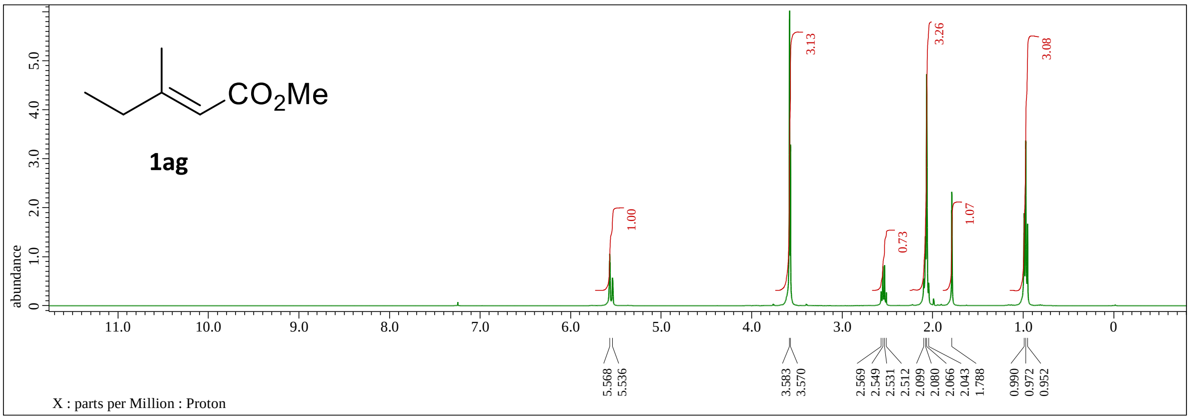

CO_2Me
1ag
abundance
5.568
5.536
3.583
3.570
2.569
2.549
2.531
2.512
2.099
2.080
2.066
2.043
1.788
0.990
0.972
0.952
1.00
3.13
0.73
3.26
1.07
3.08
X : parts per Million : Proton

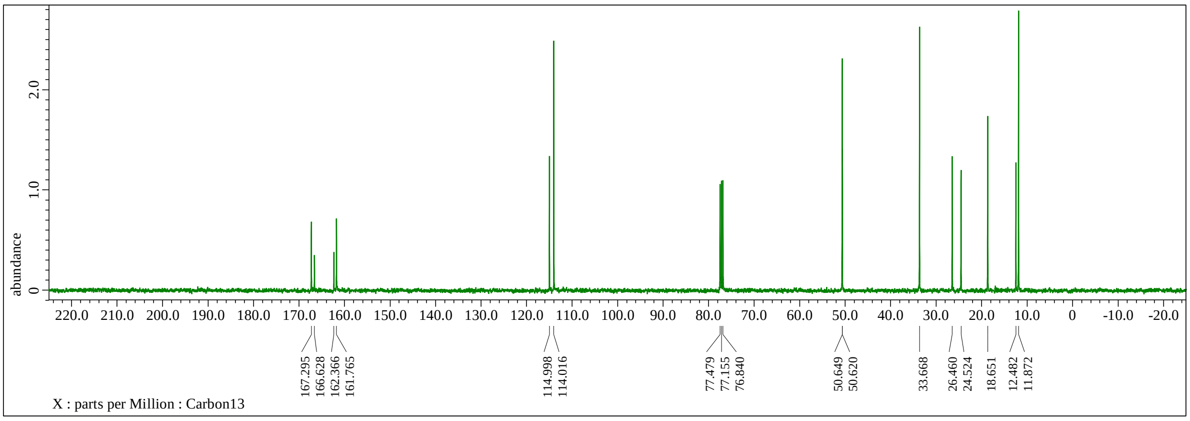

abundance
167.295
166.628
162.366
161.765
114.998
114.016
77.479
77.155
76.840
50.649
50.620
33.668
26.460
24.524
18.651
12.482
11.872
X : parts per Million : Carbon13

9.0 ^{1}H and ^{13}C NMR of the Carboxylic Acids

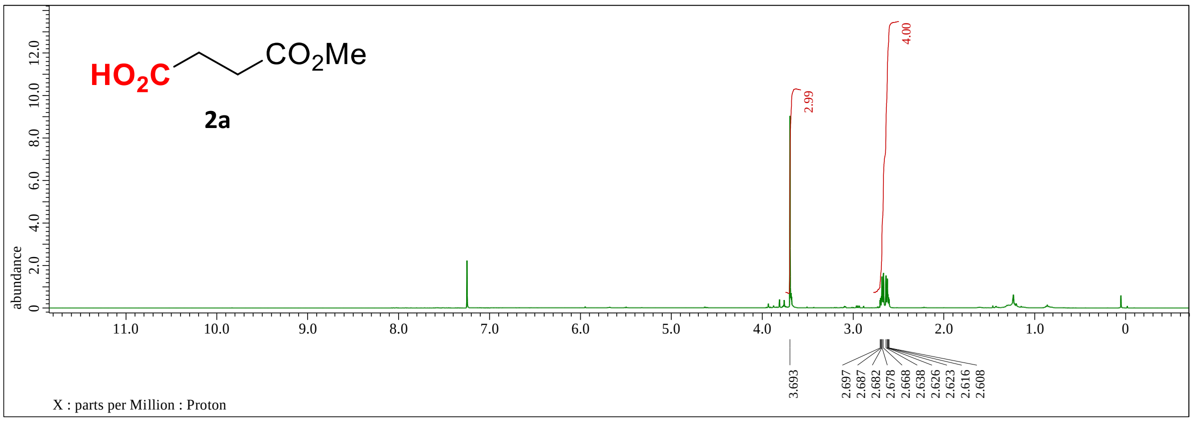

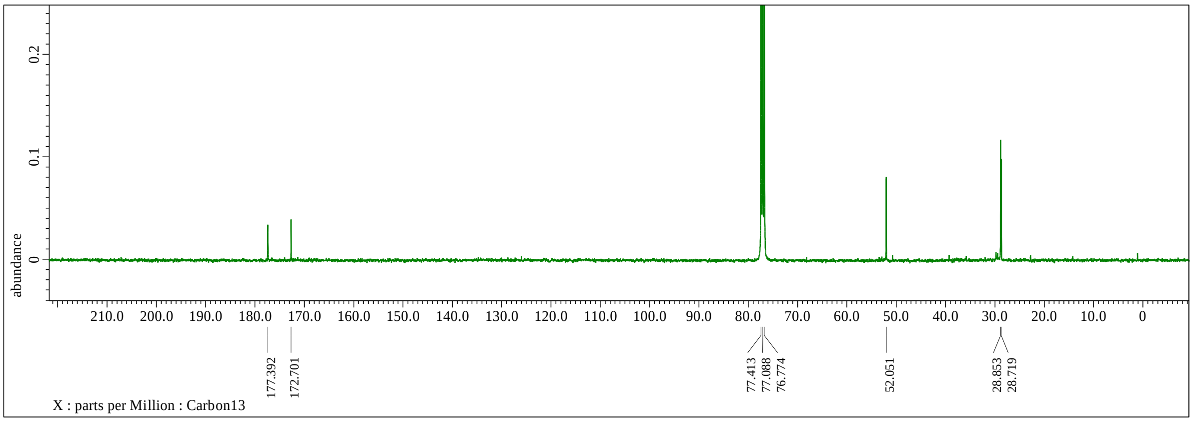

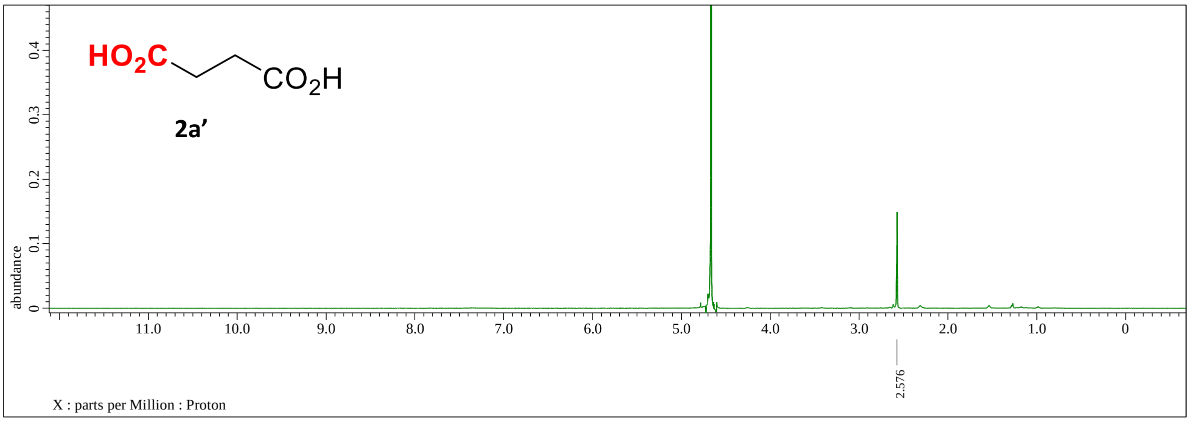

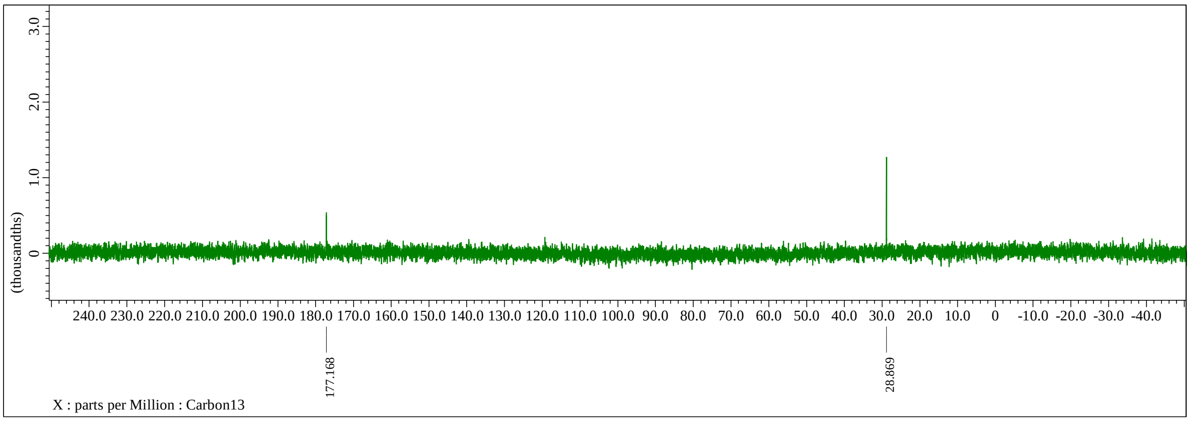

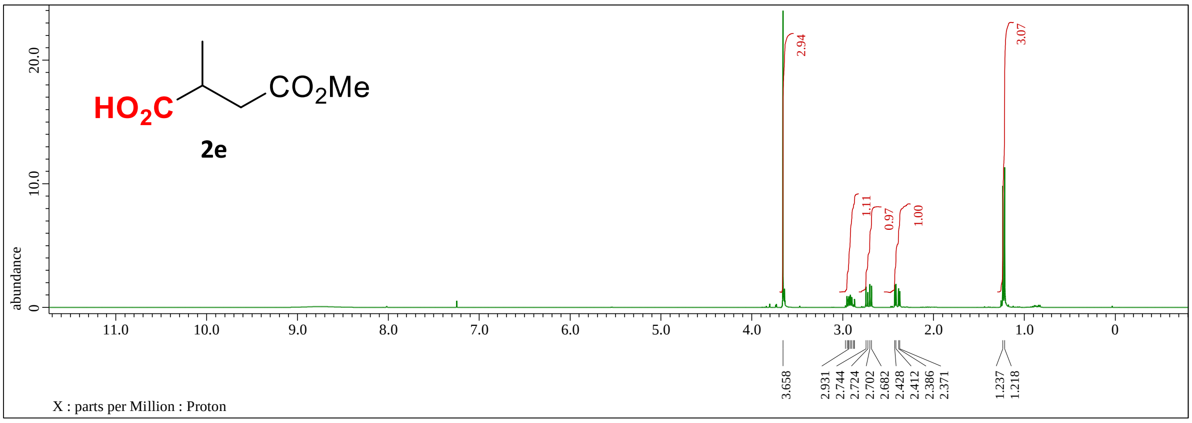
HO2C
CO2Me
2e
abundance
2.94
1.11
0.97
1.00
3.07
3.658
2.931 2.744 2.724 2.702 2.682 2.428 2.412 2.386 2.371
1.237 1.218
X : parts per Million : Proton

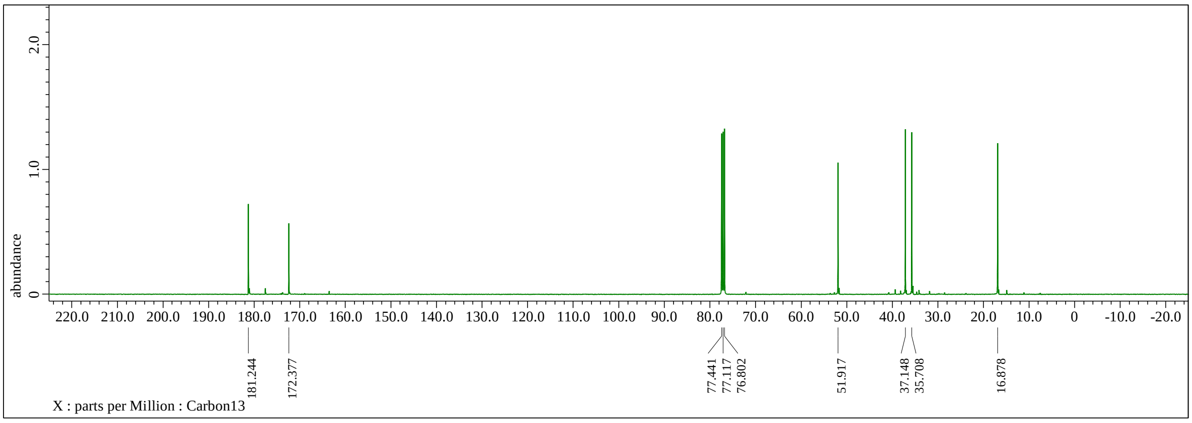
abundance
181.244
172.377
77.441 77.117 76.802
51.917
37.148 35.708
16.878
X : parts per Million : Carbon13

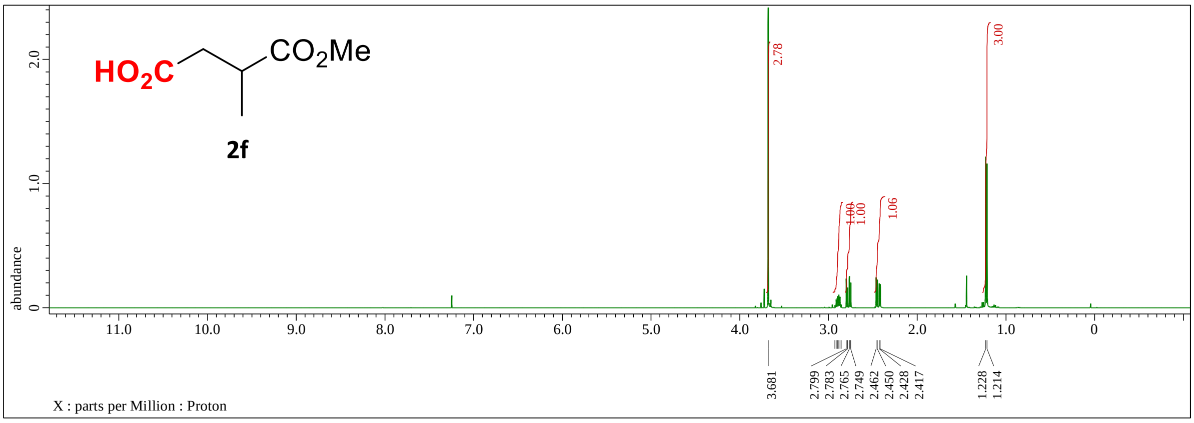
HO2C
CO2Me
2f
abundance
2.78
1.00
1.00
1.06
3.00
3.681
2.799 2.783 2.765 2.749 2.462 2.450 2.428 2.417
1.228 1.214
X : parts per Million : Proton

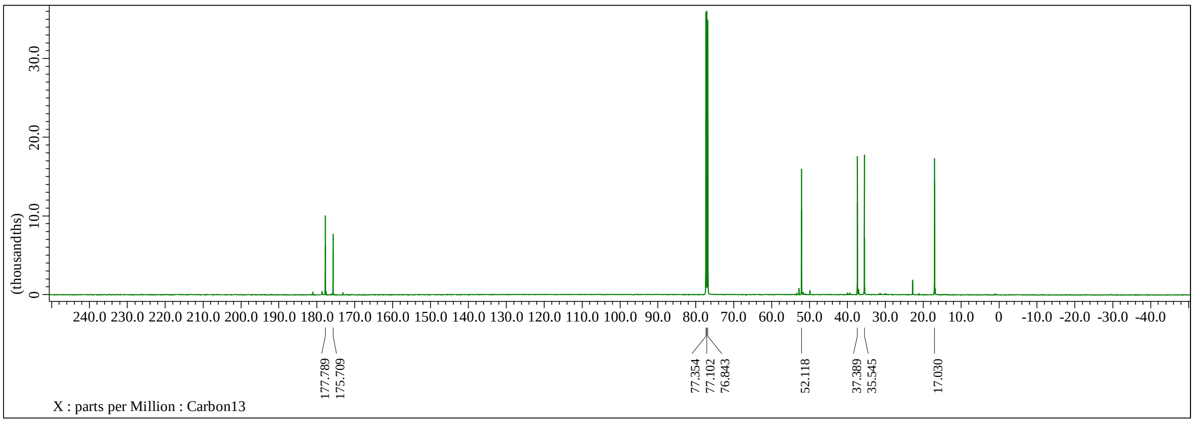
(thousandths)
177.789 175.709
77.354 77.102 76.843
52.118
37.389 35.545
17.030
X : parts per Million : Carbon13

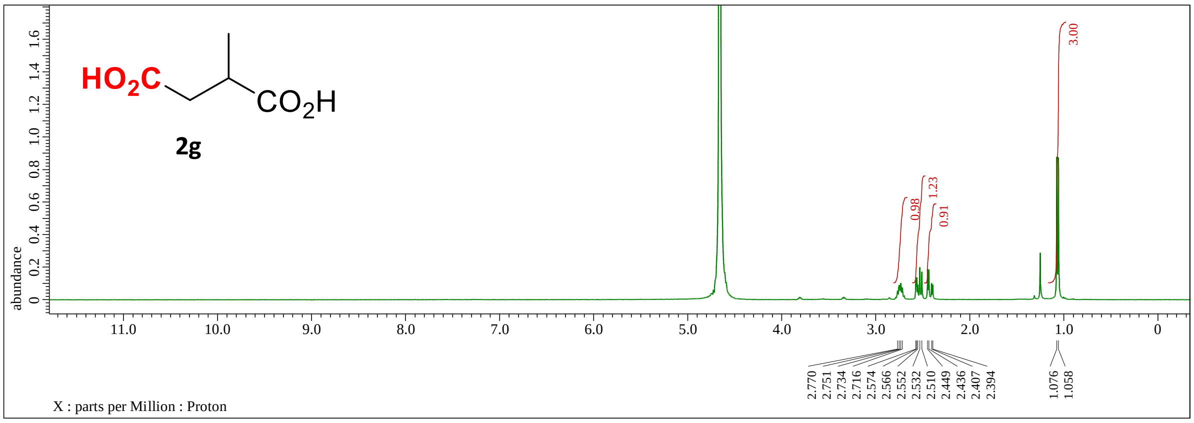
HO2C
CO2H
2g
abundance
X : parts per Million : Proton
3.00
0.98
1.23
0.91
2.770
2.751
2.734
2.716
2.574
2.566
2.552
2.532
2.510
2.449
2.436
2.407
2.394
1.076
1.058

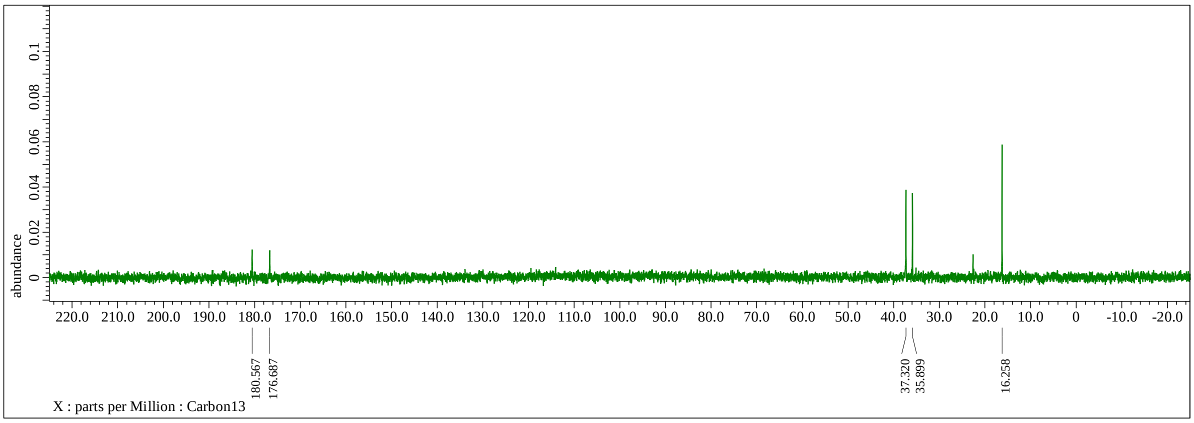
abundance
X : parts per Million : Carbon13
180.567
176.687
37.320
35.899
16.258

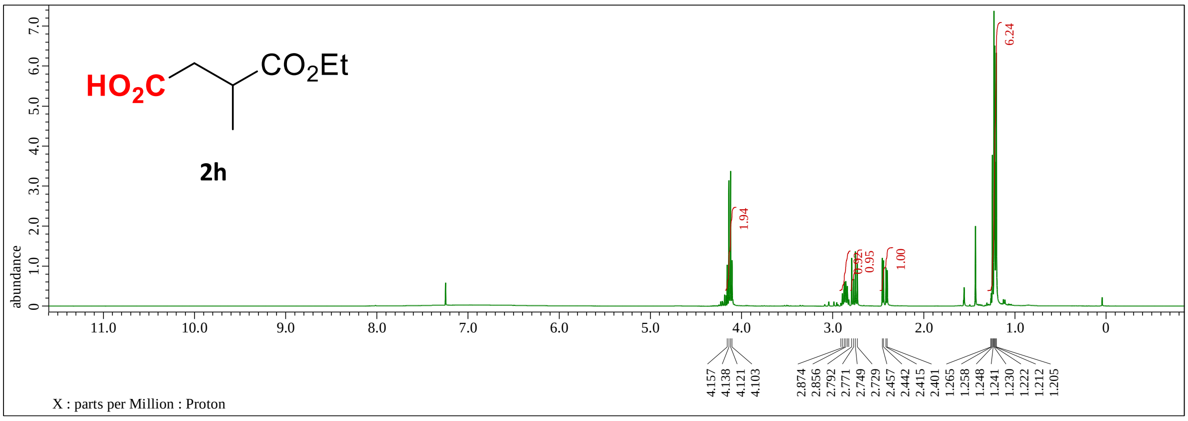
HO2C
CO2Et
2h
abundance
X : parts per Million : Proton
6.24
1.94
0.95
1.00
4.157
4.138
4.121
4.103
2.874
2.856
2.792
2.771
2.749
2.729
2.457
2.442
2.415
2.401
1.265
1.258
1.248
1.241
1.230
1.222
1.212
1.205

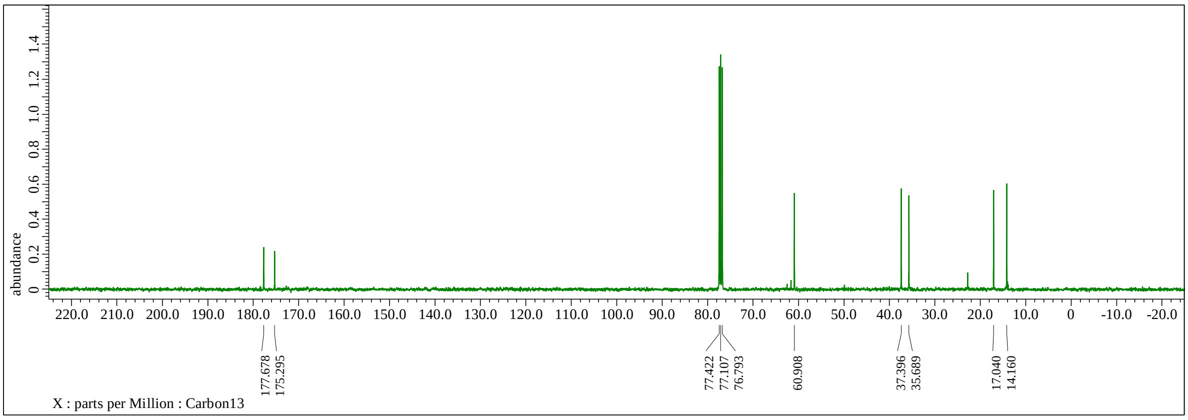
abundance
X : parts per Million : Carbon13
177.678
175.295
77.422
77.107
76.793
60.908
37.396
35.689
17.040
14.160

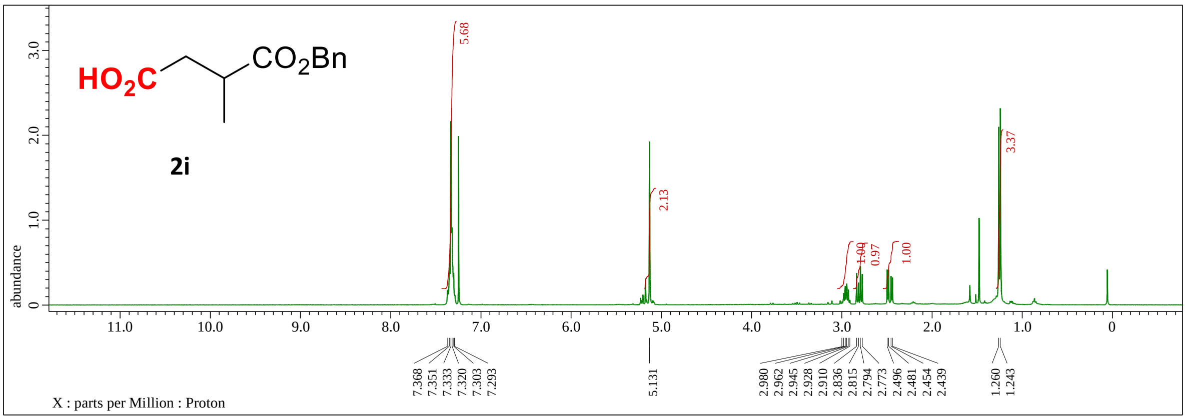
HO2C CO2Bn
2i
5.68
2.13
1.00
0.97
1.00
3.37
abundance
11.0 10.0 9.0 8.0 7.0 6.0 5.0 4.0 3.0 2.0 1.0 0
7.368 7.351 7.333 7.320 7.303 7.293
5.131
2.980 2.962 2.945 2.928 2.910 2.836 2.815 2.794 2.773 2.496 2.481 2.454 2.439
1.260 1.243
X : parts per Million : Proton

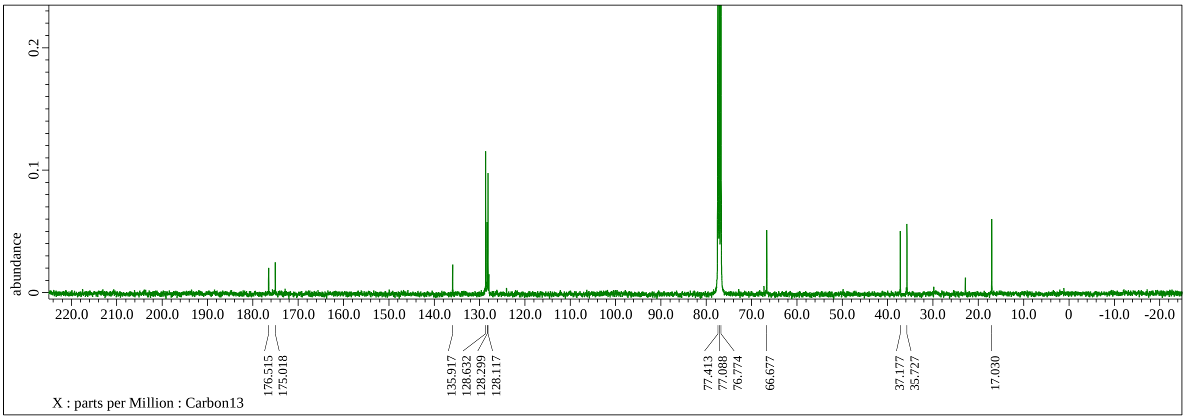
abundance
220.0 210.0 200.0 190.0 180.0 170.0 160.0 150.0 140.0 130.0 120.0 110.0 100.0 90.0 80.0 70.0 60.0 50.0 40.0 30.0 20.0 10.0 0 -10.0 -20.0
176.515 175.018
135.917 128.632 128.299 128.117
77.413 77.088 76.774
66.677
37.177 35.727
17.030
X : parts per Million : Carbon13

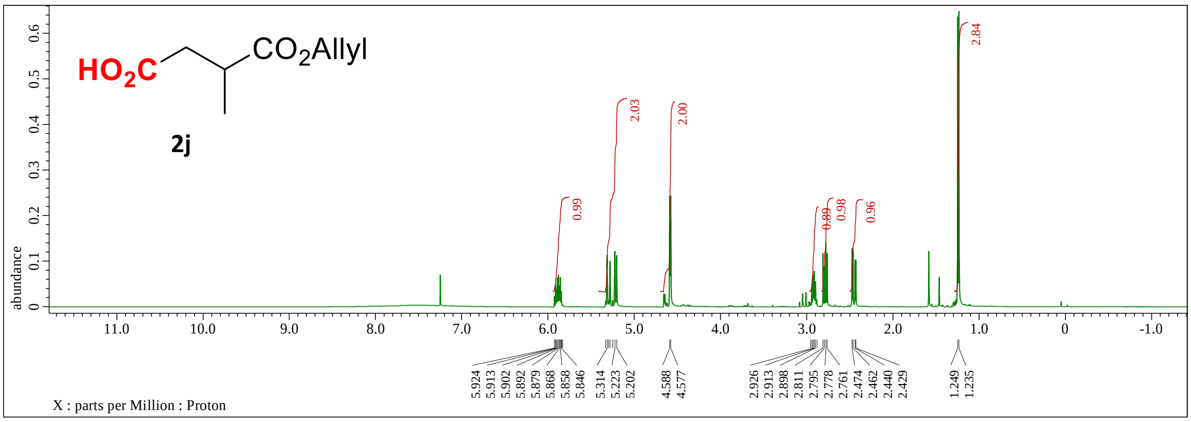
HO2C CO2Allyl
2j
0.99
2.03
2.00
0.89
0.98
0.96
2.84
abundance
11.0 10.0 9.0 8.0 7.0 6.0 5.0 4.0 3.0 2.0 1.0 0 -1.0
5.924 5.913 5.902 5.892 5.879 5.868 5.858 5.846
5.314 5.223 5.202
4.588 4.577
2.926 2.913 2.898 2.811 2.795 2.778 2.761 2.474 2.462 2.440 2.429
1.249 1.235
X : parts per Million : Proton

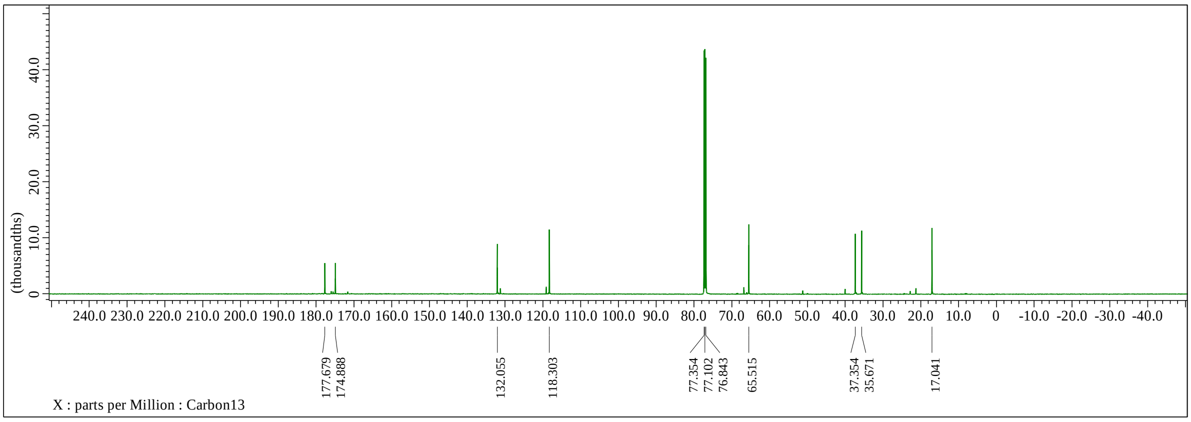
(thousandths)
240.0 230.0 220.0 210.0 200.0 190.0 180.0 170.0 160.0 150.0 140.0 130.0 120.0 110.0 100.0 90.0 80.0 70.0 60.0 50.0 40.0 30.0 20.0 10.0 0 -10.0 -20.0 -30.0 -40.0
177.679 174.888
132.055
118.303
77.354 77.102 76.843
65.515
37.354 35.671
17.041
X : parts per Million : Carbon13

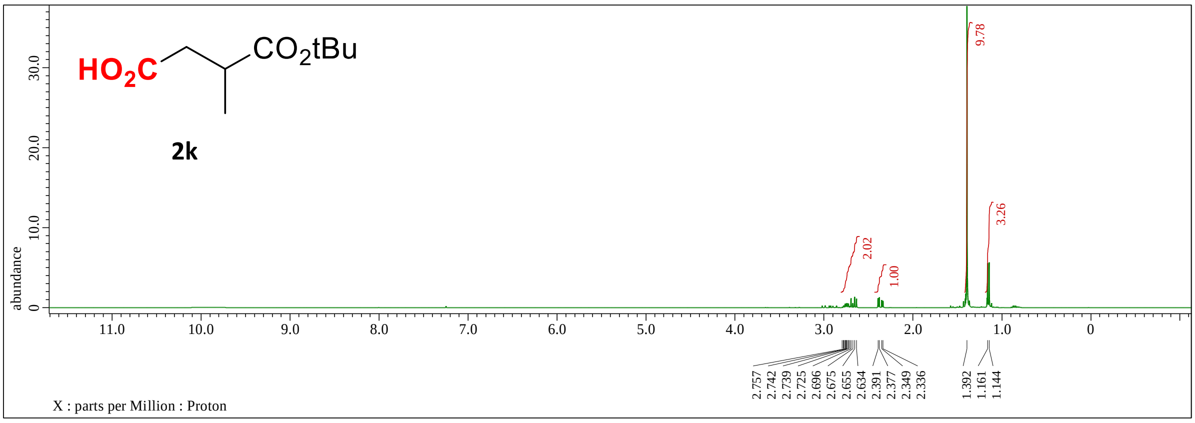

HO2C
CO2tBu
2k
abundance
9.78
3.26
2.02
1.00
2.757 2.742 2.739 2.725 2.696 2.675 2.655 2.634 2.391 2.377 2.349 2.336
1.392 1.161 1.144
X : parts per Million : Proton

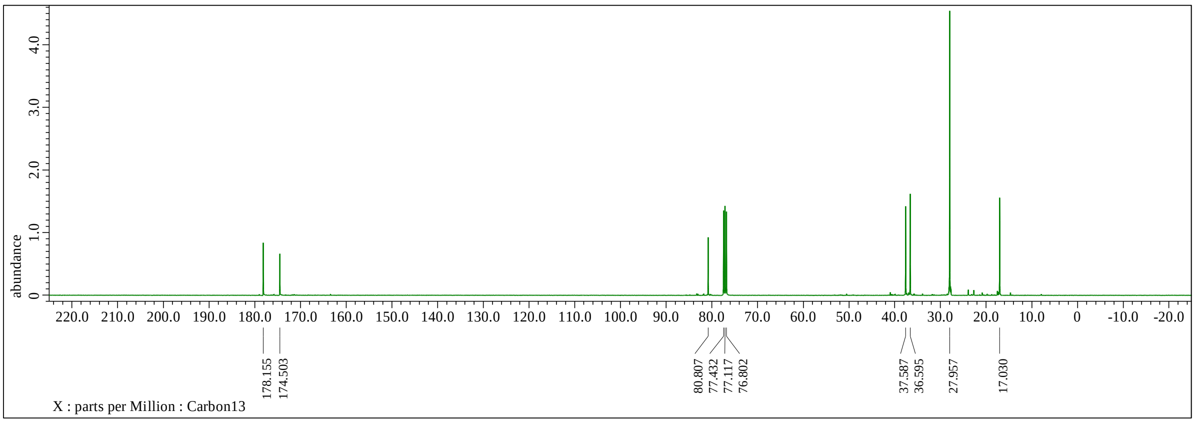

abundance
178.155 174.503
80.807 77.432 77.117 76.802
37.587 36.595
27.957
17.030
X : parts per Million : Carbon13

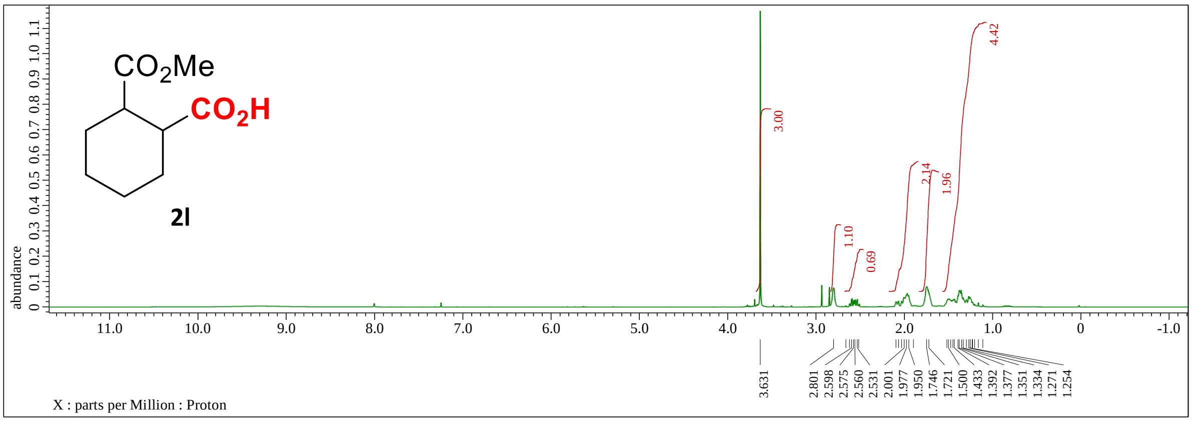

CO2Me
CO2H
2l
abundance
3.00
1.10
0.69
2.14
1.96
4.42
3.631
2.801 2.598 2.575 2.560 2.531 2.001 1.977 1.950 1.746 1.721 1.500 1.433 1.392 1.377 1.351 1.334 1.271 1.254
X : parts per Million : Proton

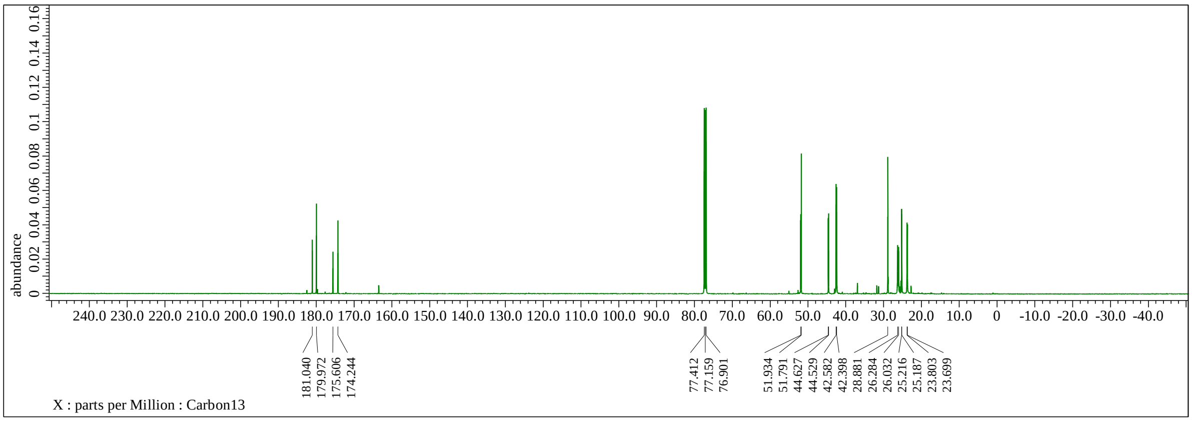

abundance
181.040 179.972 175.606 174.244
77.412 77.159 76.901
51.934 51.791 44.627 44.529 42.582 42.398 28.881 26.284 26.032 25.216 25.187 23.803 23.699
X : parts per Million : Carbon13

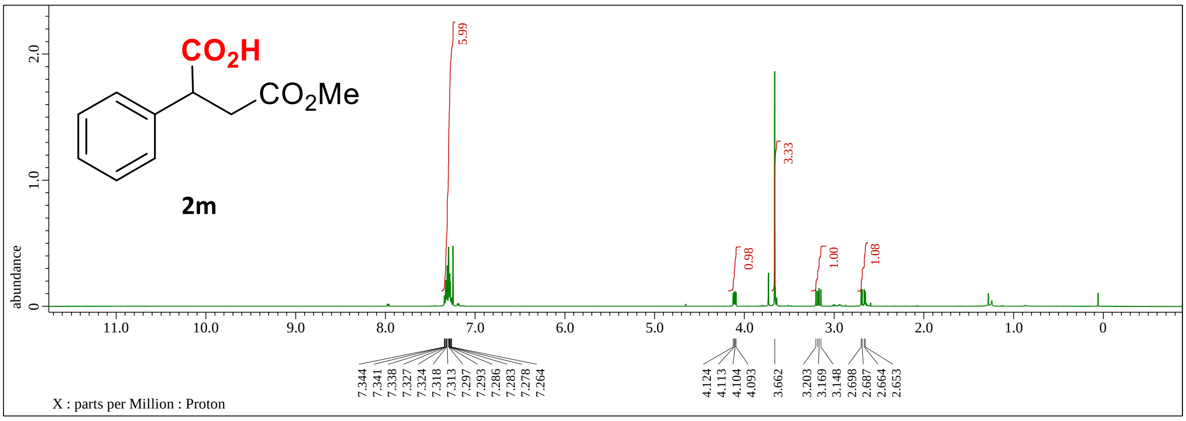
CO2H
CO2Me
2m
5.99
3.33
0.98
1.00
1.08
abundance
7.344 7.341 7.338 7.327 7.324 7.318 7.313 7.297 7.293 7.286 7.283 7.278 7.264
4.124 4.113 4.104 4.093
3.662
3.203 3.169 3.148 2.698 2.687 2.664 2.653
X : parts per Million : Proton

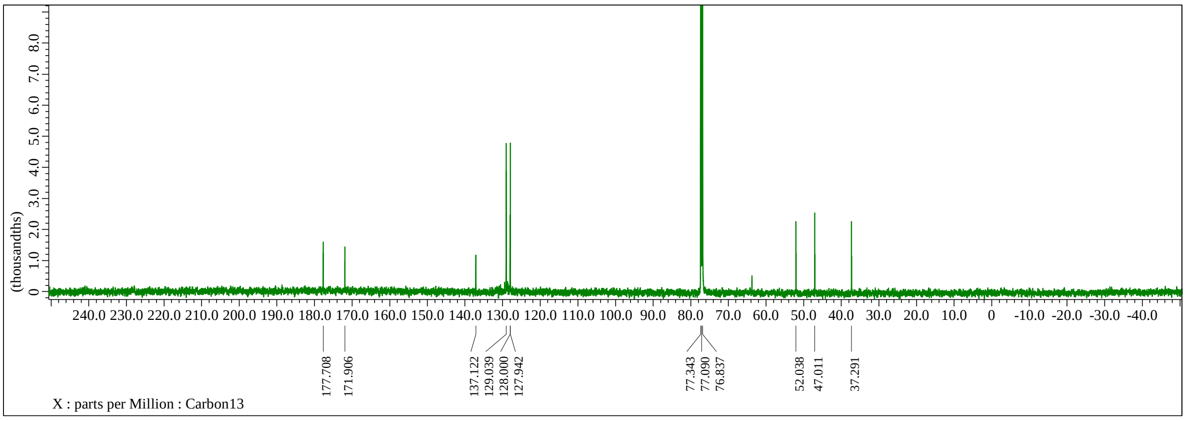
(thousandths)
177.708
171.906
137.122 129.039 128.000 127.942
77.343 77.090 76.837
52.038
47.011
37.291
X : parts per Million : Carbon13

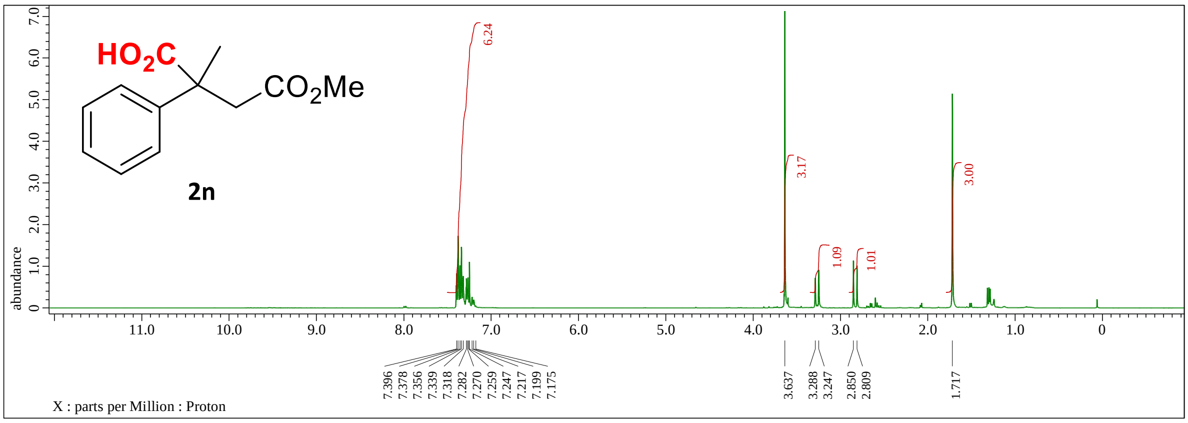
HO2C
CO2Me
2n
6.24
3.17
1.09
1.01
3.00
abundance
7.396 7.378 7.356 7.339 7.318 7.282 7.270 7.259 7.247 7.217 7.199 7.175
3.637
3.288 3.247
2.850 2.809
1.717
X : parts per Million : Proton

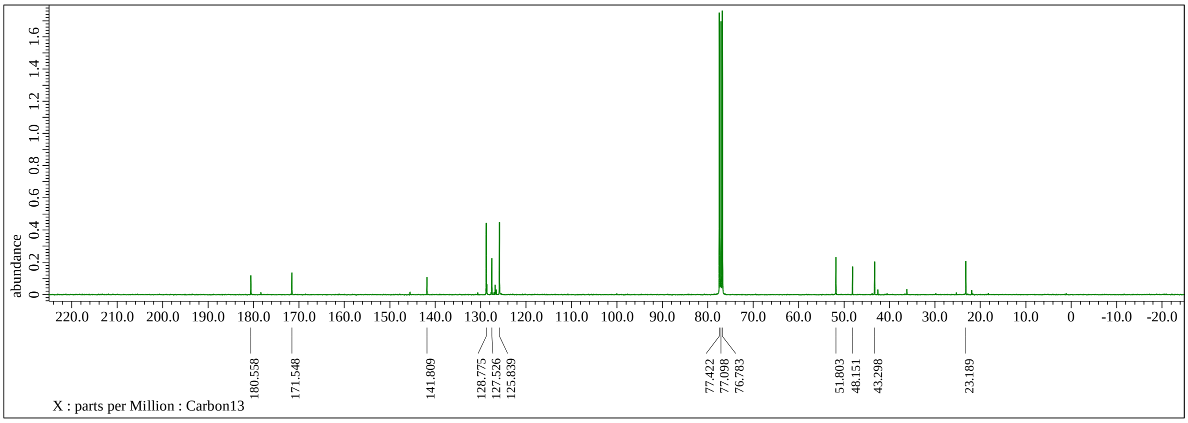
abundance
180.558
171.548
141.809
128.775 127.526 125.839
77.422 77.098 76.783
51.803 48.151 43.298
23.189
X : parts per Million : Carbon13

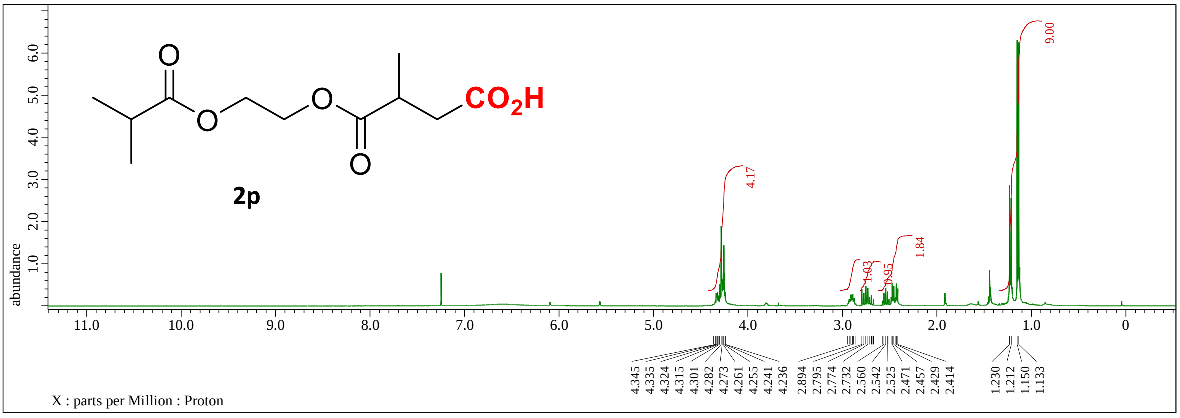
CO2H
2p
4.17
1.93
0.95
1.84
9.00
4.345 4.335 4.324 4.315 4.301 4.282 4.273 4.261 4.255 4.241 4.236
2.894 2.795 2.774 2.732 2.560 2.542 2.525 2.471 2.457 2.429 2.414
1.230 1.212 1.150 1.133
abundance
X : parts per Million : Proton

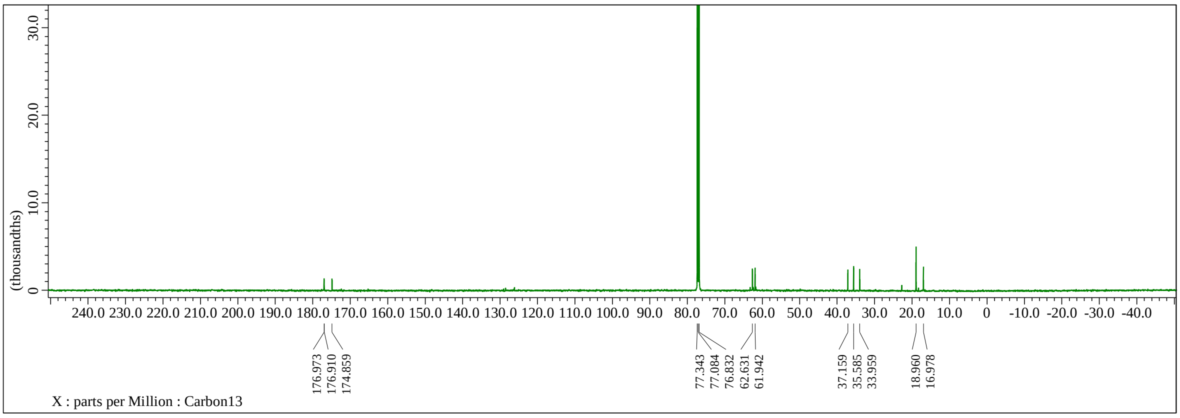
176.973 176.910 174.859
77.343 77.084 76.832 62.631 61.942
37.159 35.585 33.959
18.960 16.978
(thousandths)
X : parts per Million : Carbon13

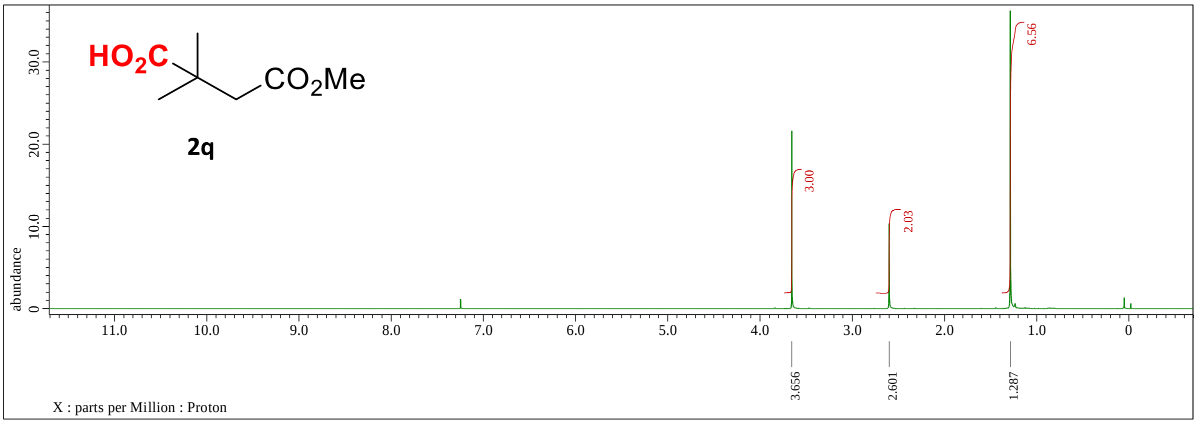
HO2C
CO2Me
2q
3.00
2.03
6.56
3.656
2.601
1.287
abundance
X : parts per Million : Proton

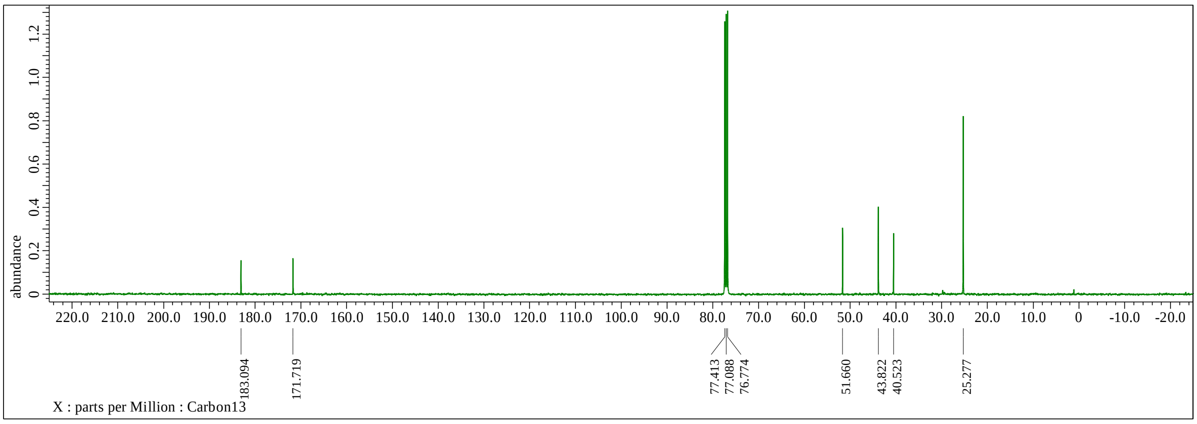
183.094
171.719
77.413 77.088 76.774
51.660
43.822 40.523
25.277
abundance
X : parts per Million : Carbon13

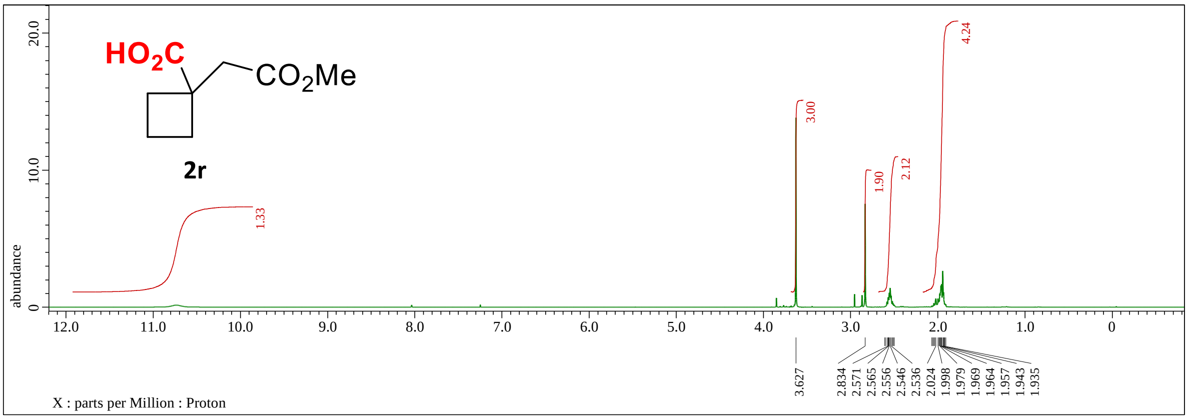
HO2C
CO2Me
2r
abundance
1.33
3.00
1.90
2.12
4.24
3.627
2.834
2.571
2.565
2.556
2.546
2.536
2.024
1.998
1.979
1.969
1.964
1.957
1.943
1.935
X : parts per Million : Proton

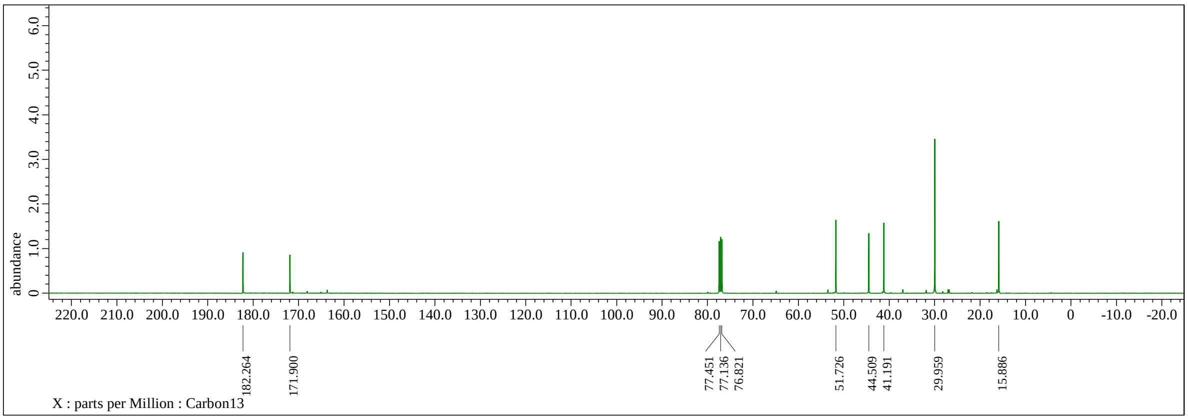
abundance
182.264
171.900
77.451
77.136
76.821
51.726
44.509
41.191
29.959
15.886
X : parts per Million : Carbon13

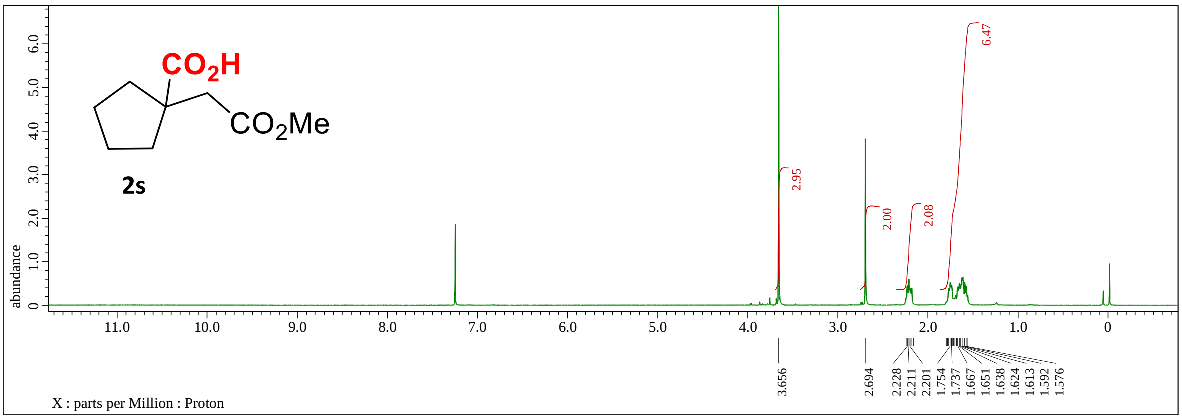
CO2H
CO2Me
2s
abundance
2.95
2.00
2.08
6.47
3.656
2.694
2.228
2.211
2.201
1.754
1.737
1.667
1.651
1.638
1.624
1.613
1.592
1.576
X : parts per Million : Proton

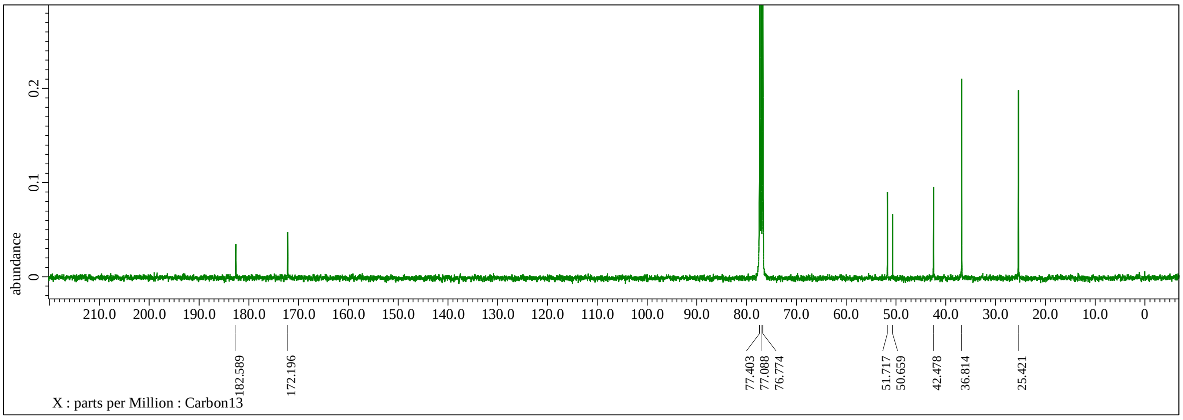
abundance
182.589
172.196
77.403
77.088
76.774
51.717
50.659
42.478
36.814
25.421
X : parts per Million : Carbon13

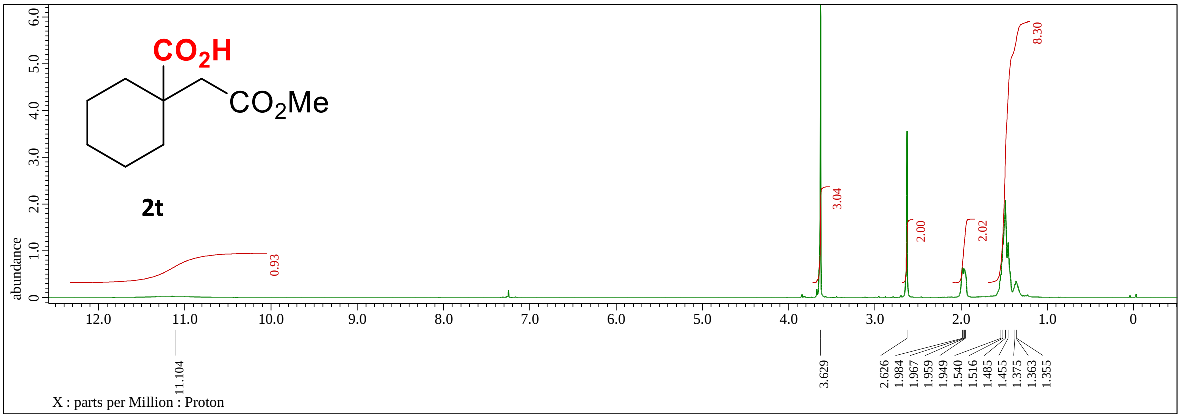
CO2H
CO2Me
2t
abundance
0.93
3.04
2.00
2.02
8.30
11.104
3.629
2.626 1.984 1.967 1.959 1.949 1.540 1.516 1.485 1.455 1.375 1.363 1.355
X : parts per Million : Proton

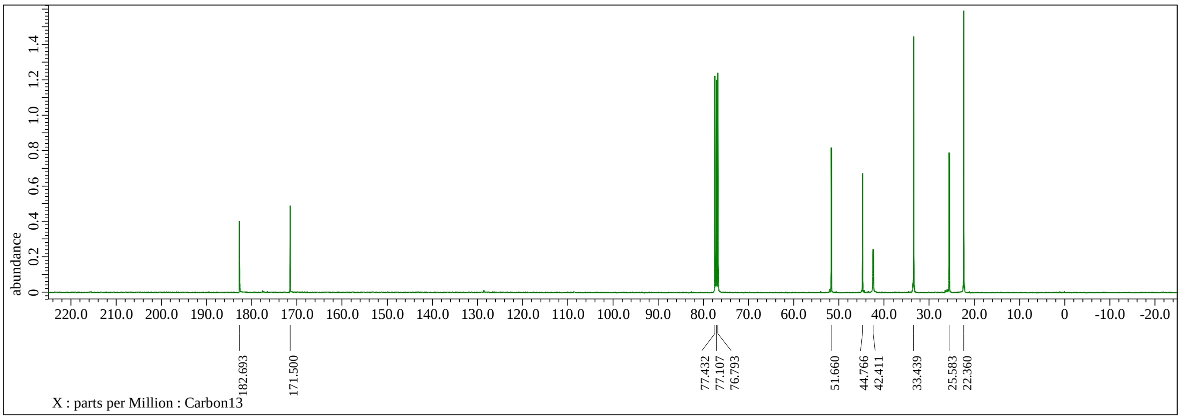
abundance
182.693
171.500
77.432 77.107 76.793
51.660
44.766 42.411
33.439
25.583 22.360
X : parts per Million : Carbon13

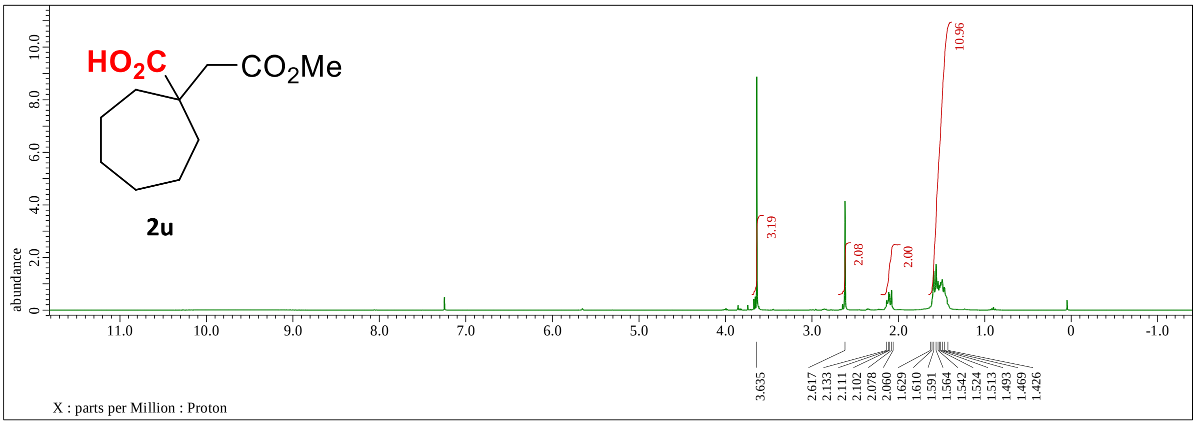
HO2C
CO2Me
2u
abundance
3.19
2.08
2.00
10.96
3.635
2.617 2.133 2.111 2.102 2.078 2.060 1.629 1.610 1.591 1.564 1.542 1.524 1.513 1.493 1.469 1.426
X : parts per Million : Proton

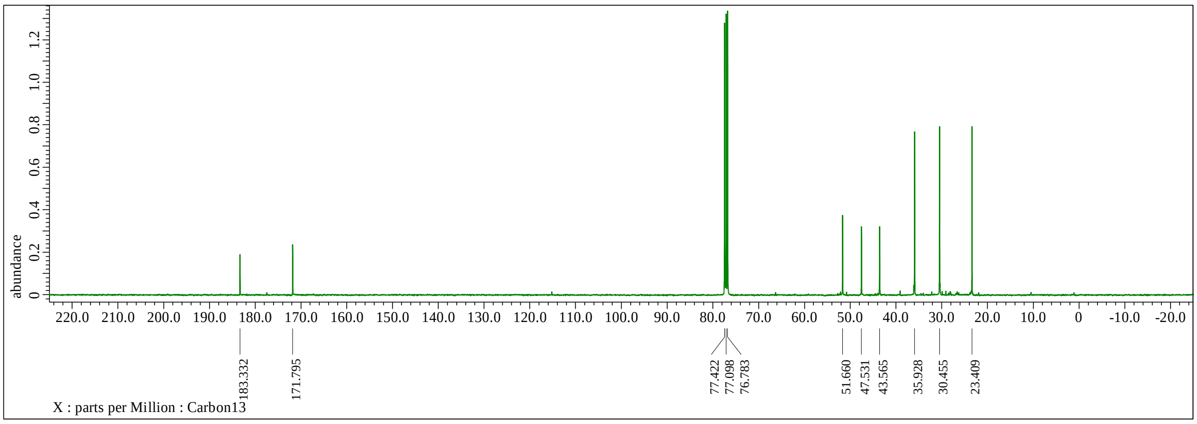
abundance
183.332
171.795
77.422 77.098 76.783
51.660 47.531 43.565
35.928
30.455
23.409
X : parts per Million : Carbon13

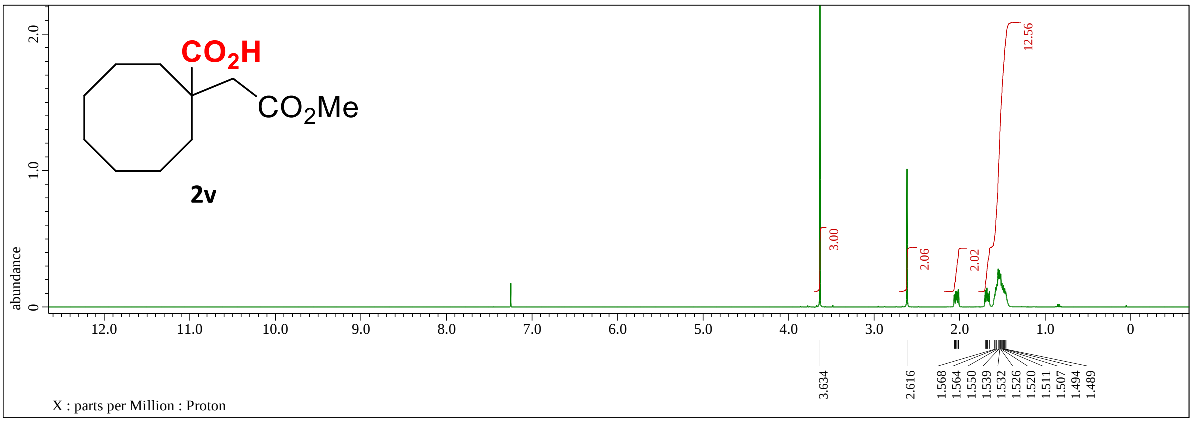
CO2H
CO2Me
2v
3.00
2.06
2.02
12.56
abundance
3.634
2.616
1.568 1.564 1.550 1.539 1.532 1.526 1.520 1.511 1.507 1.494 1.489
X : parts per Million : Proton

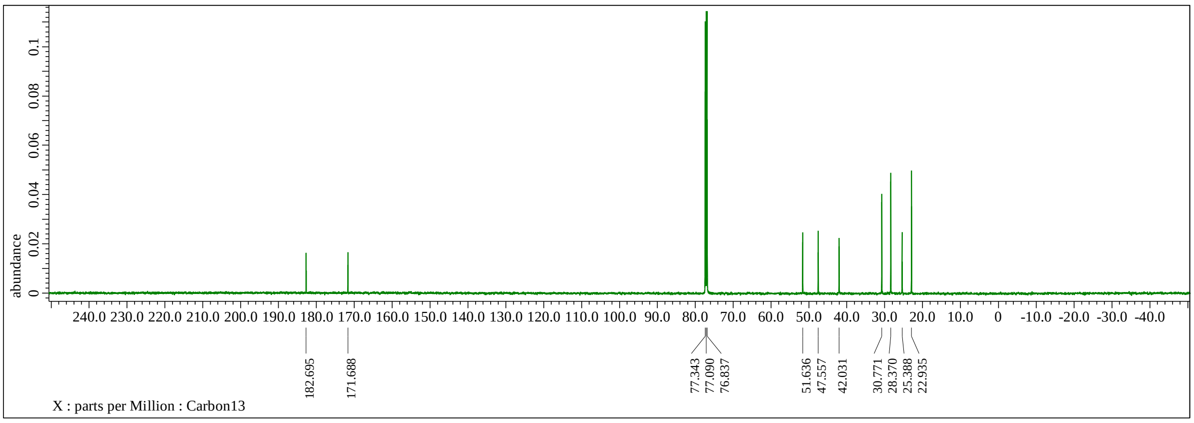
abundance
182.695
171.688
77.343 77.090 76.837
51.636 47.557 42.031
30.771 28.370 25.388 22.935
X : parts per Million : Carbon13

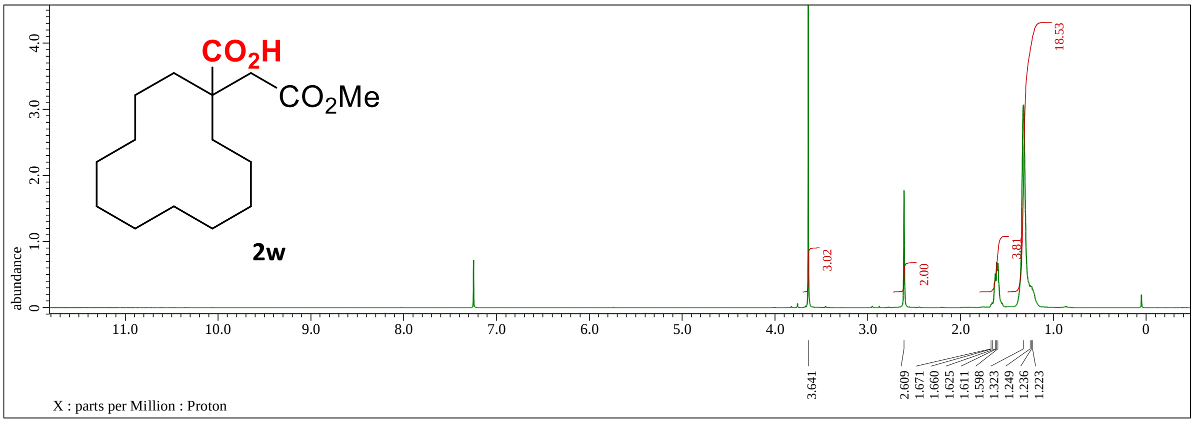
CO2H
CO2Me
2w
3.02
2.00
3.81
18.53
abundance
3.641
2.609 1.671 1.660 1.625 1.611 1.598 1.323 1.249 1.236 1.223
X : parts per Million : Proton

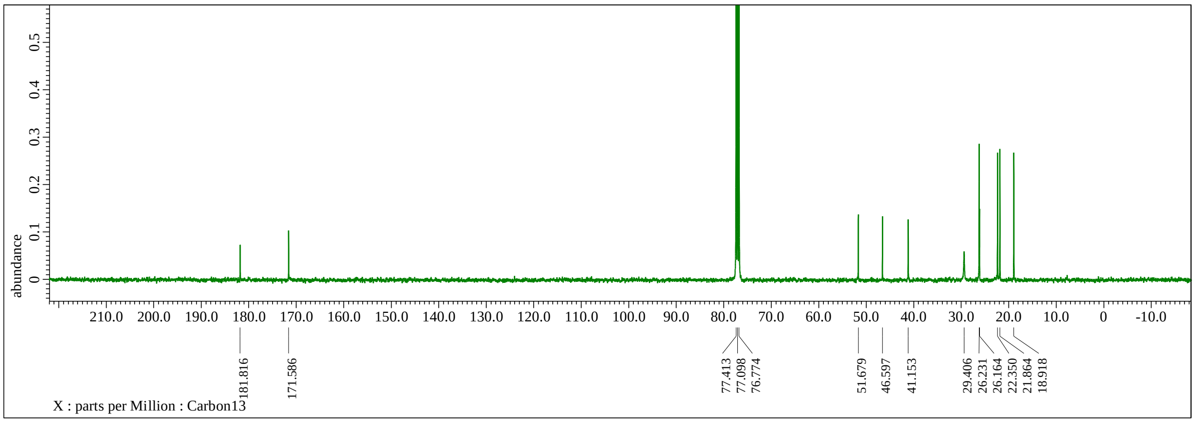
abundance
181.816
171.586
77.413 77.098 76.774
51.679 46.597 41.153
29.406 26.231 26.164 22.350 21.864 18.918
X : parts per Million : Carbon13

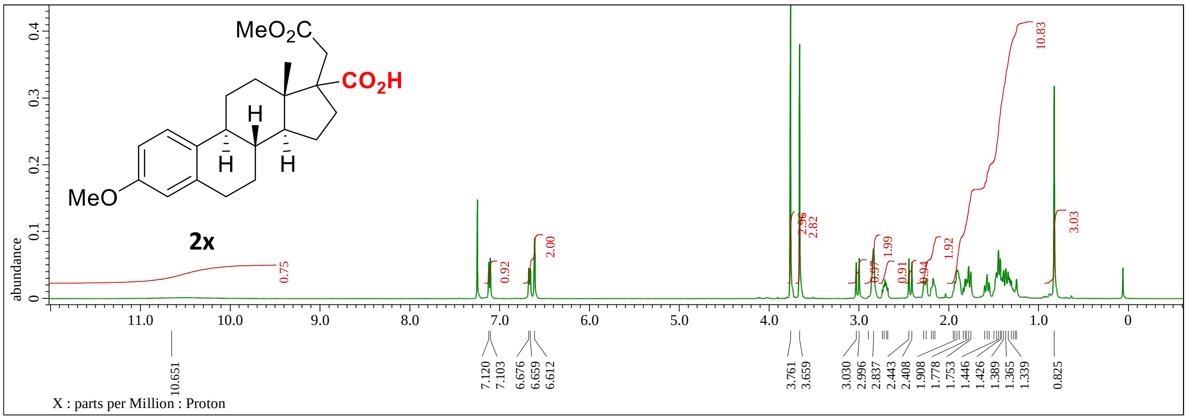

MeO2C
CO2H
H
H
H
MeO
2x
abundance
0.75
0.92
2.00
2.96
2.82
0.97
1.99
0.91
0.94
1.92
10.83
3.03
10.651
7.120
7.103
6.676
6.659
6.612
3.761
3.659
3.030
2.996
2.837
2.443
2.408
1.908
1.778
1.753
1.446
1.426
1.389
1.365
1.339
0.825
X : parts per Million : Proton

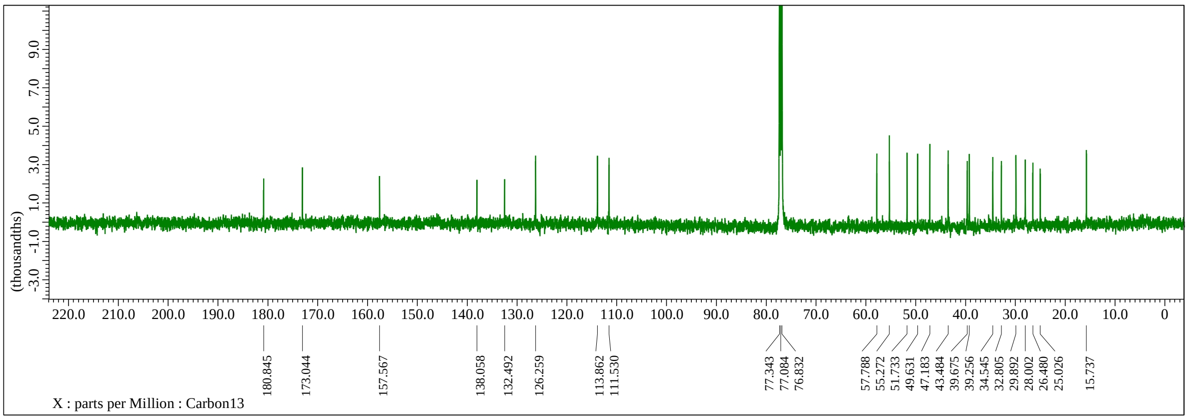

(thousandths)
180.845
173.044
157.567
138.058
132.492
126.259
113.862
111.530
77.343
77.084
76.832
57.788
55.272
51.733
49.631
47.183
43.484
39.675
39.256
34.545
32.805
29.892
28.002
26.480
25.026
15.737
X : parts per Million : Carbon13

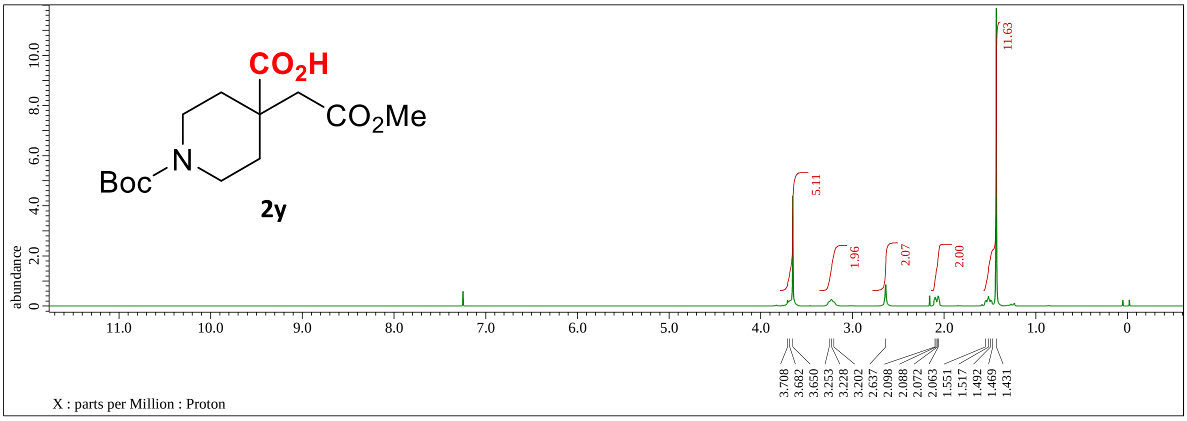

CO2H
CO2Me
N
Boc
2y
abundance
5.11
1.96
2.07
2.00
11.63
3.708
3.682
3.650
3.253
3.228
3.202
2.637
2.098
2.088
2.072
2.063
1.551
1.517
1.492
1.469
1.431
X : parts per Million : Proton

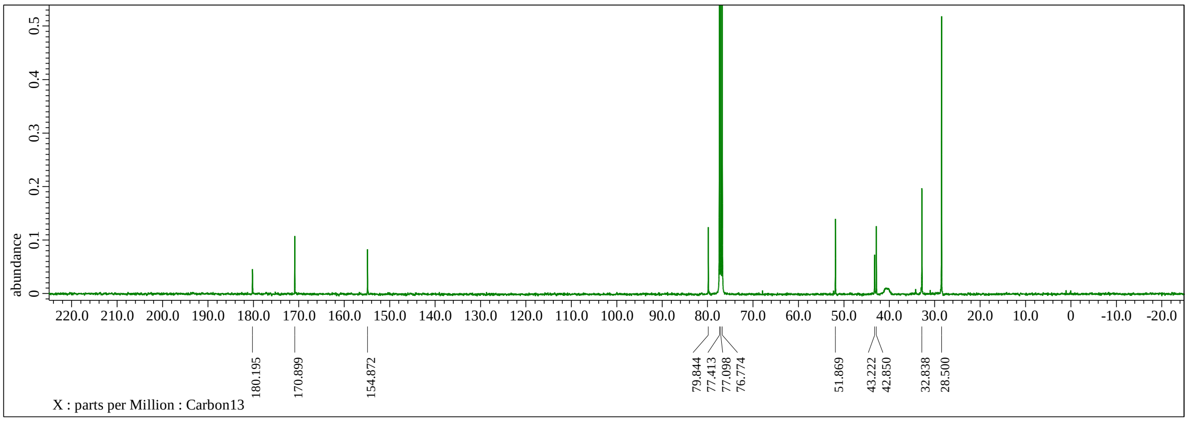

abundance
180.195
170.899
154.872
79.844
77.413
77.098
76.774
51.869
43.222
42.850
32.838
28.500
X : parts per Million : Carbon13

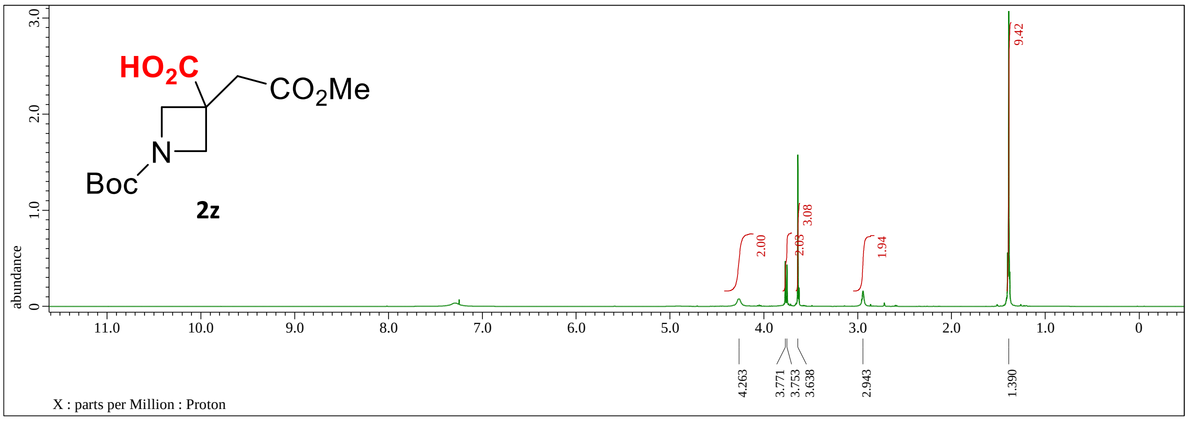
HO2C
CO2Me
N
Boc
2z
abundance
X : parts per Million : Proton
2.00
2.03
3.08
1.94
9.42
4.263
3.771
3.753
3.638
2.943
1.390

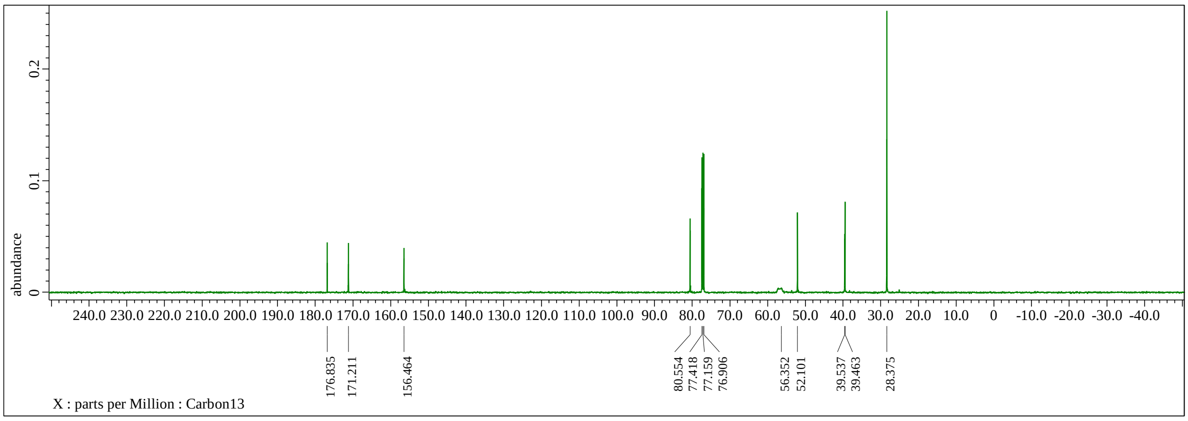
abundance
X : parts per Million : Carbon13
176.835
171.211
156.464
80.554
77.418
77.159
76.906
56.352
52.101
39.537
39.463
28.375

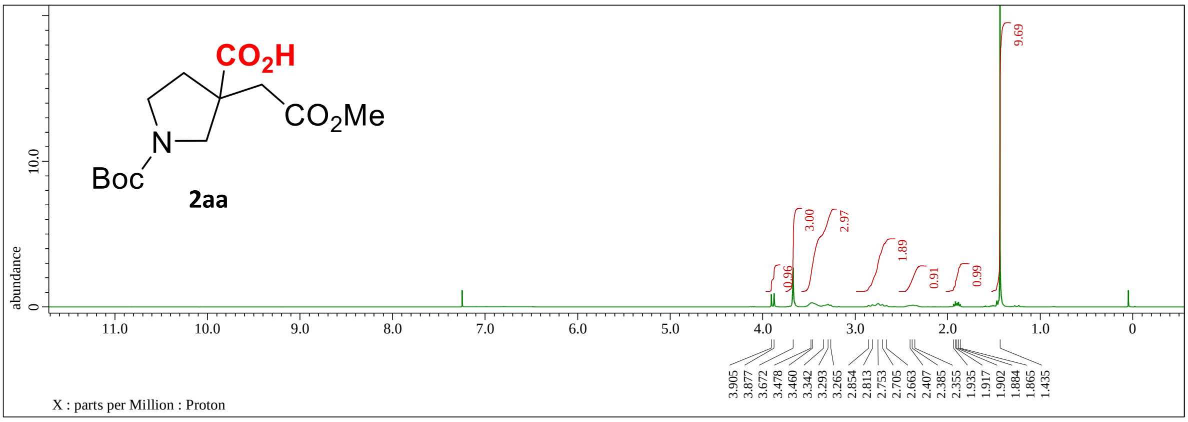
CO2H
CO2Me
N
Boc
2aa
abundance
X : parts per Million : Proton
0.96
3.00
2.97
1.89
0.91
0.99
9.69
3.905
3.877
3.672
3.478
3.460
3.342
3.293
3.265
2.854
2.813
2.753
2.705
2.663
2.407
2.385
2.355
1.935
1.917
1.902
1.884
1.865
1.435

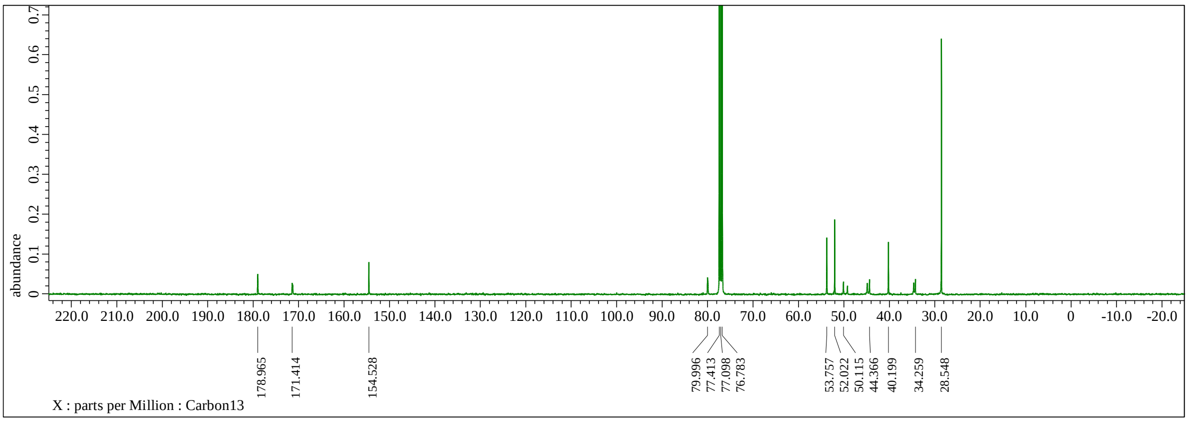
abundance
X : parts per Million : Carbon13
178.965
171.414
154.528
79.996
77.413
77.098
76.783
53.757
52.022
50.115
44.366
40.199
34.259
28.548

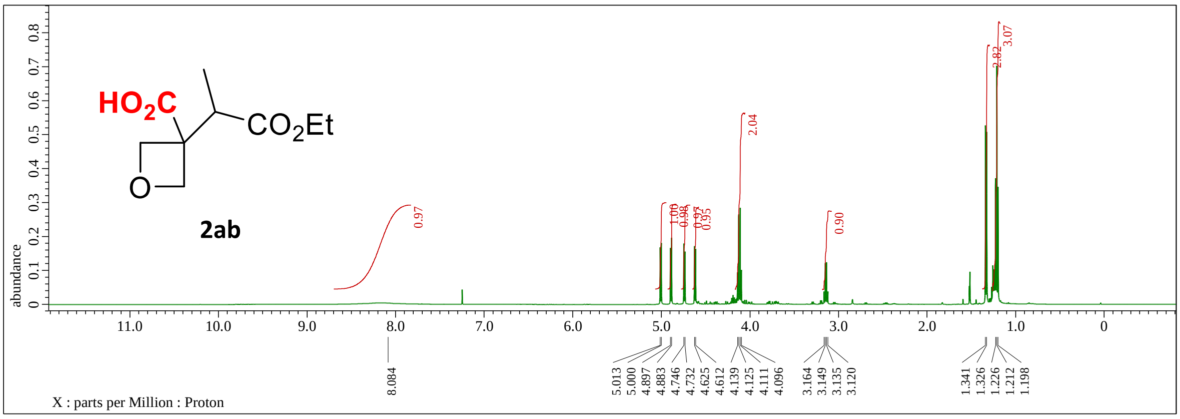
HO2C
CO2Et
O
2ab
0.97
2.04
0.90
3.07
8.084
5.013 5.000 4.897 4.883 4.746 4.732 4.625 4.612 4.139 4.125 4.111 4.096
3.164 3.149 3.135 3.120
1.341 1.326 1.226 1.212 1.198
abundance
X : parts per Million : Proton

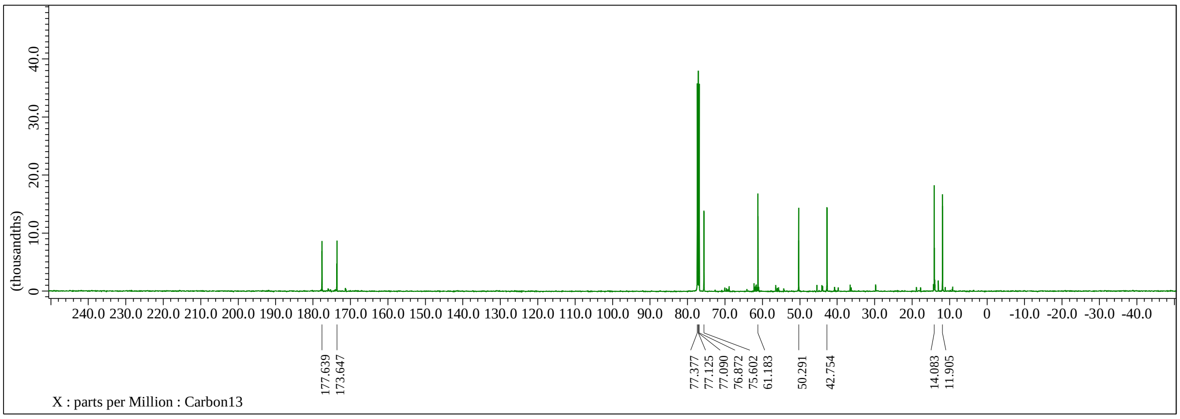
177.639 173.647
77.377 77.125 77.090 76.872 75.602 61.183
50.291 42.754
14.083 11.905
(thousandths)
X : parts per Million : Carbon13

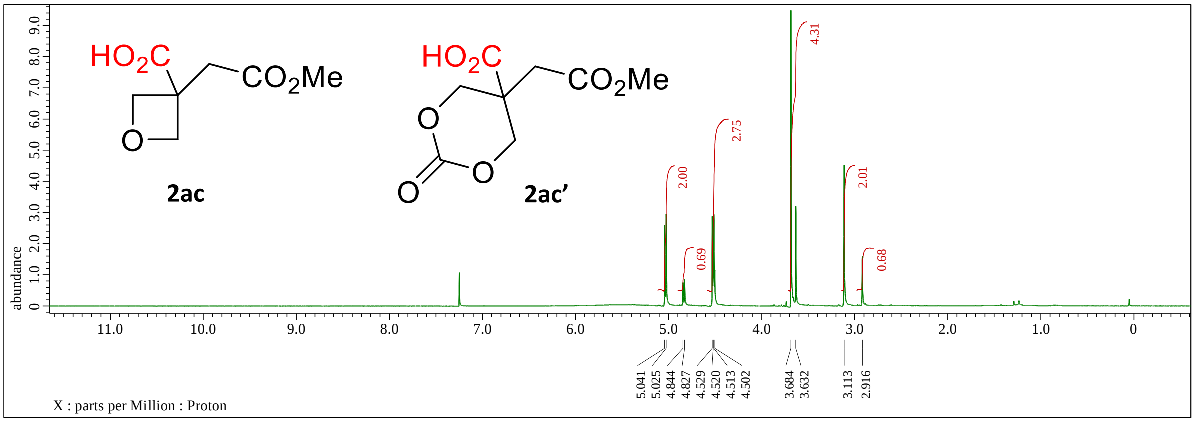
HO2C
CO2Me
O
2ac
HO2C
CO2Me
O
O
O
2ac'
4.31
2.75
2.00
0.69
2.01
0.68
5.041 5.025 4.844 4.827 4.529 4.520 4.513 4.502
3.684 3.632
3.113 2.916
abundance
X : parts per Million : Proton

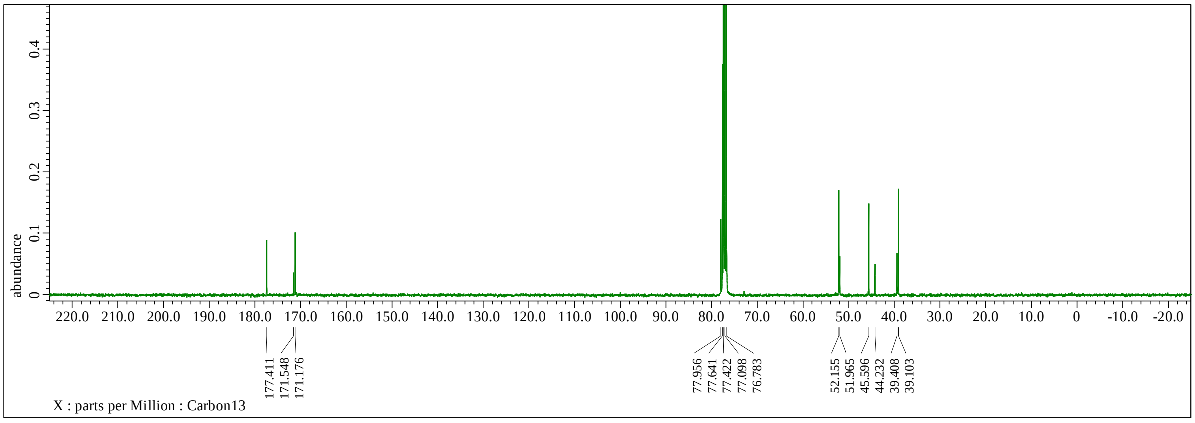
177.411 171.548 171.176
77.956 77.641 77.422 77.098 76.783
52.155 51.965 45.596 44.232 39.408 39.103
abundance
X : parts per Million : Carbon13

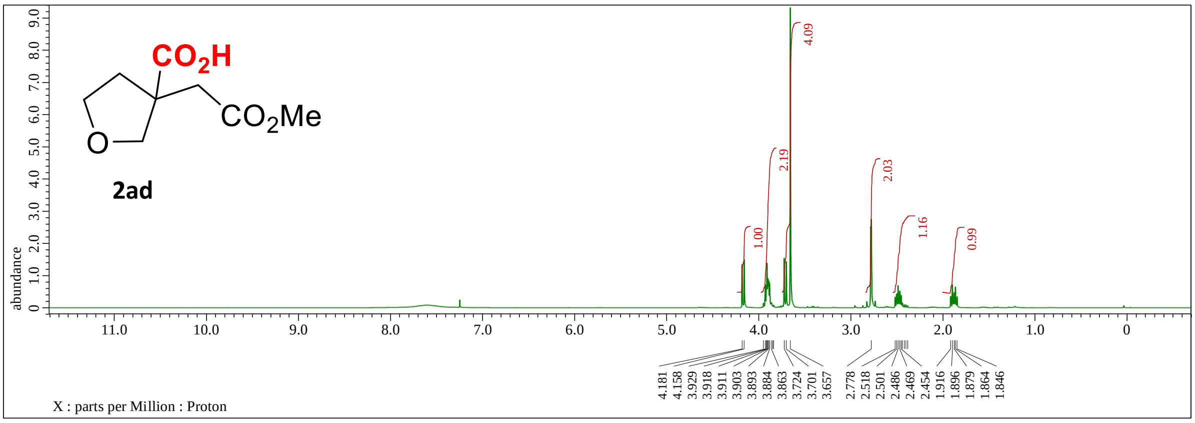
CO2H
CO2Me
O
2ad
4.09
2.19
1.00
2.03
1.16
0.99
4.181 4.158 3.929 3.918 3.911 3.903 3.893 3.884 3.863 3.724 3.701 3.657 2.778 2.518 2.501 2.486 2.469 2.454 1.916 1.896 1.879 1.864 1.846
abundance
X : parts per Million : Proton

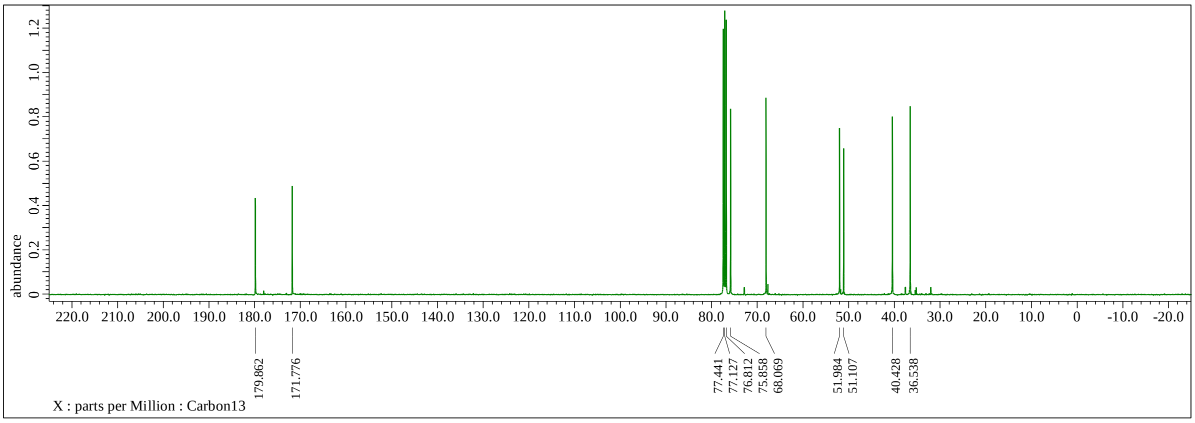
179.862
171.776
77.441 77.127 76.812 75.858 68.069
51.984 51.107
40.428 36.538
abundance
X : parts per Million : Carbon13

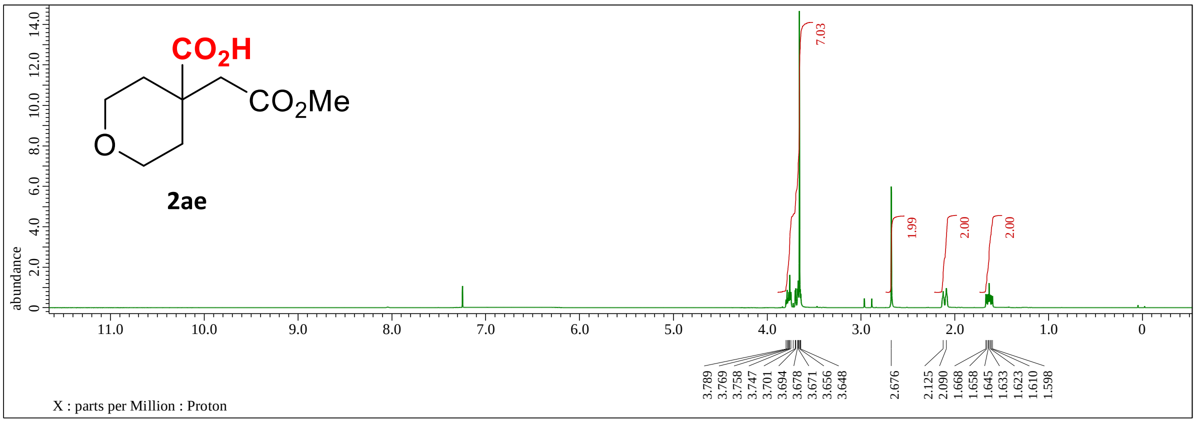
CO2H
CO2Me
O
2ae
7.03
1.99
2.00
2.00
3.789 3.769 3.758 3.747 3.701 3.694 3.678 3.671 3.656 3.648
2.676
2.125 2.090 1.668 1.658 1.645 1.633 1.623 1.610 1.598
abundance
X : parts per Million : Proton

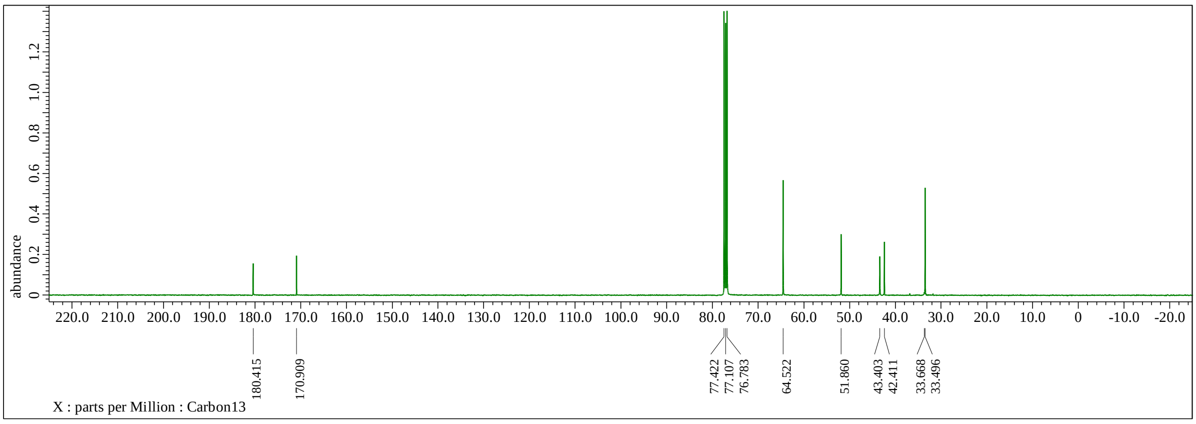
180.415
170.909
77.422 77.107 76.783
64.522
51.860
43.403 42.411
33.668 33.496
abundance
X : parts per Million : Carbon13

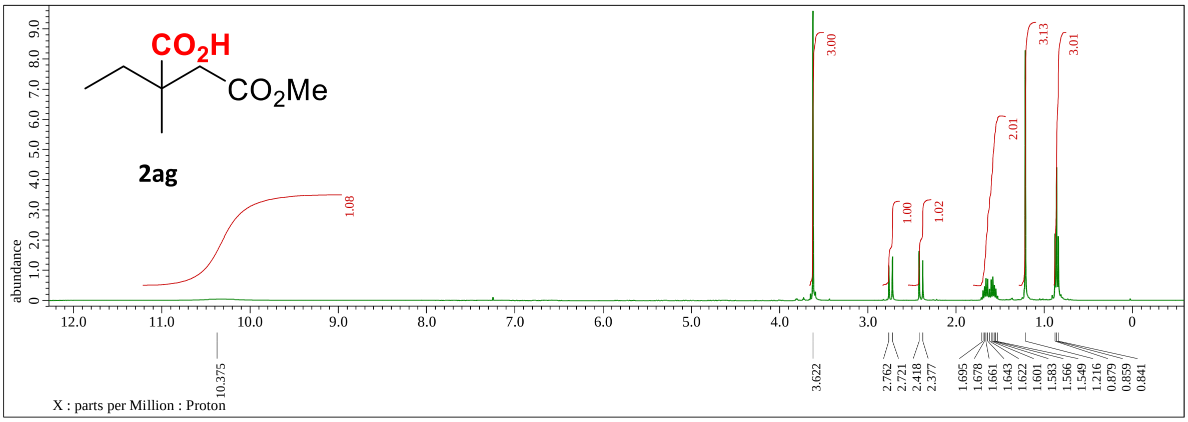
CO2H
CO2Me
2ag
abundance
X : parts per Million : Proton
10.375
3.622
2.762
2.721
2.418
2.377
1.695
1.678
1.661
1.643
1.622
1.601
1.583
1.566
1.549
1.216
0.879
0.859
0.841
1.08
3.00
1.00
1.02
2.01
3.13
3.01

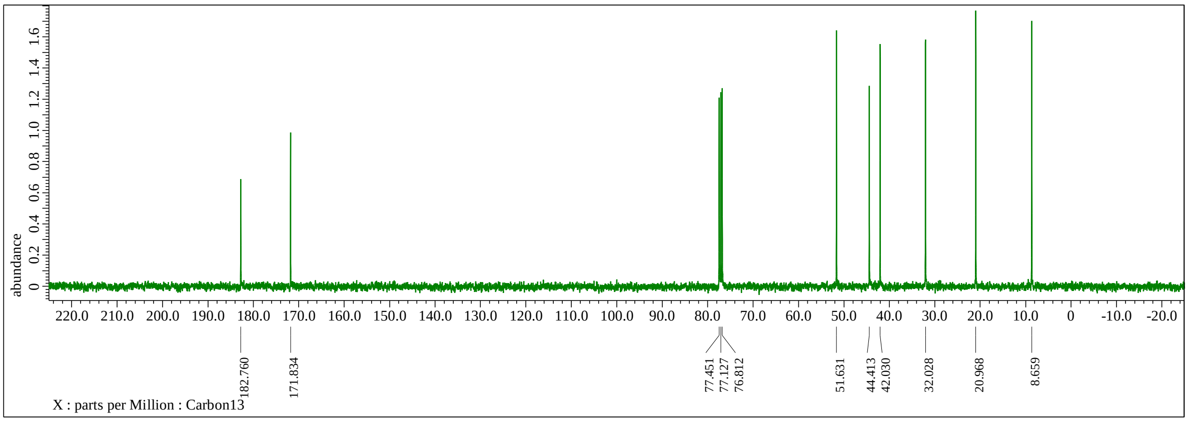
abundance
X : parts per Million : Carbon13
182.760
171.834
77.451
77.127
76.812
51.631
44.413
42.030
32.028
20.968
8.659

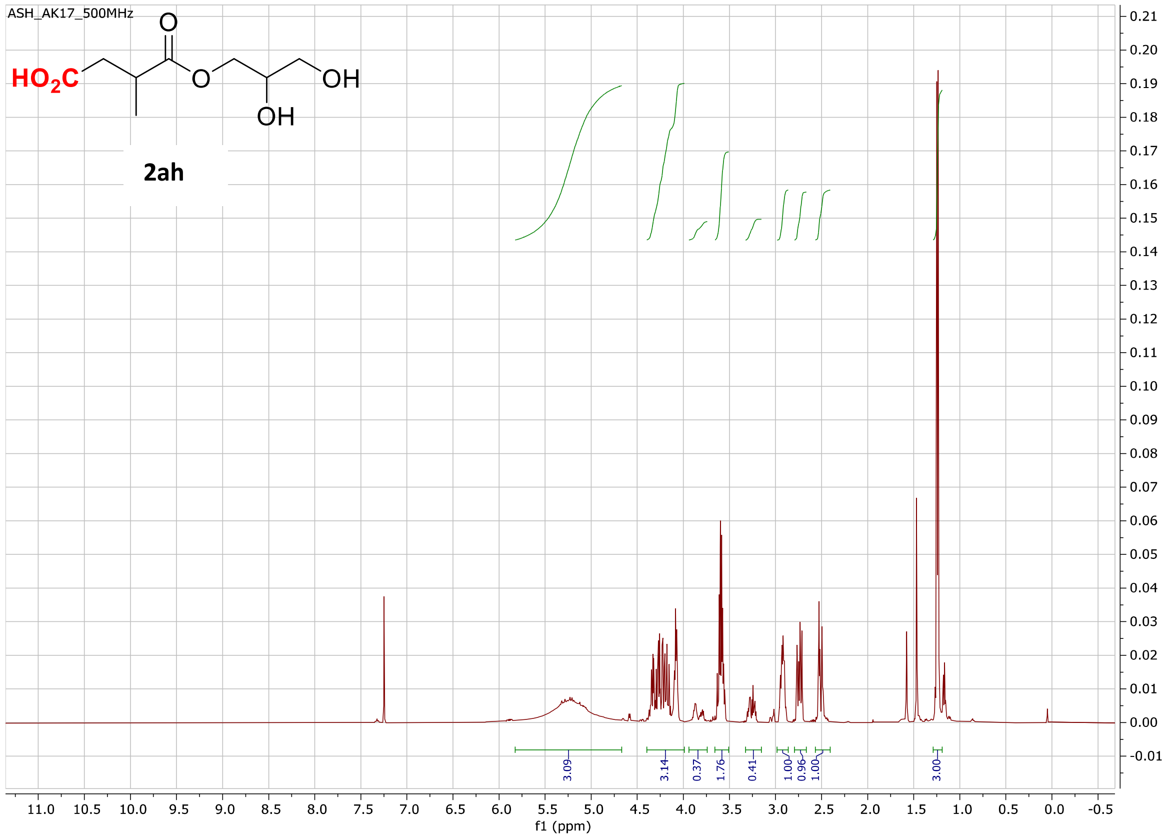
ASH_AK17_500MHz
HO2C
O
OH
OH
2ah
f1 (ppm)
3.09
3.14
0.37
1.76
0.41
1.00
0.96
1.00
3.00

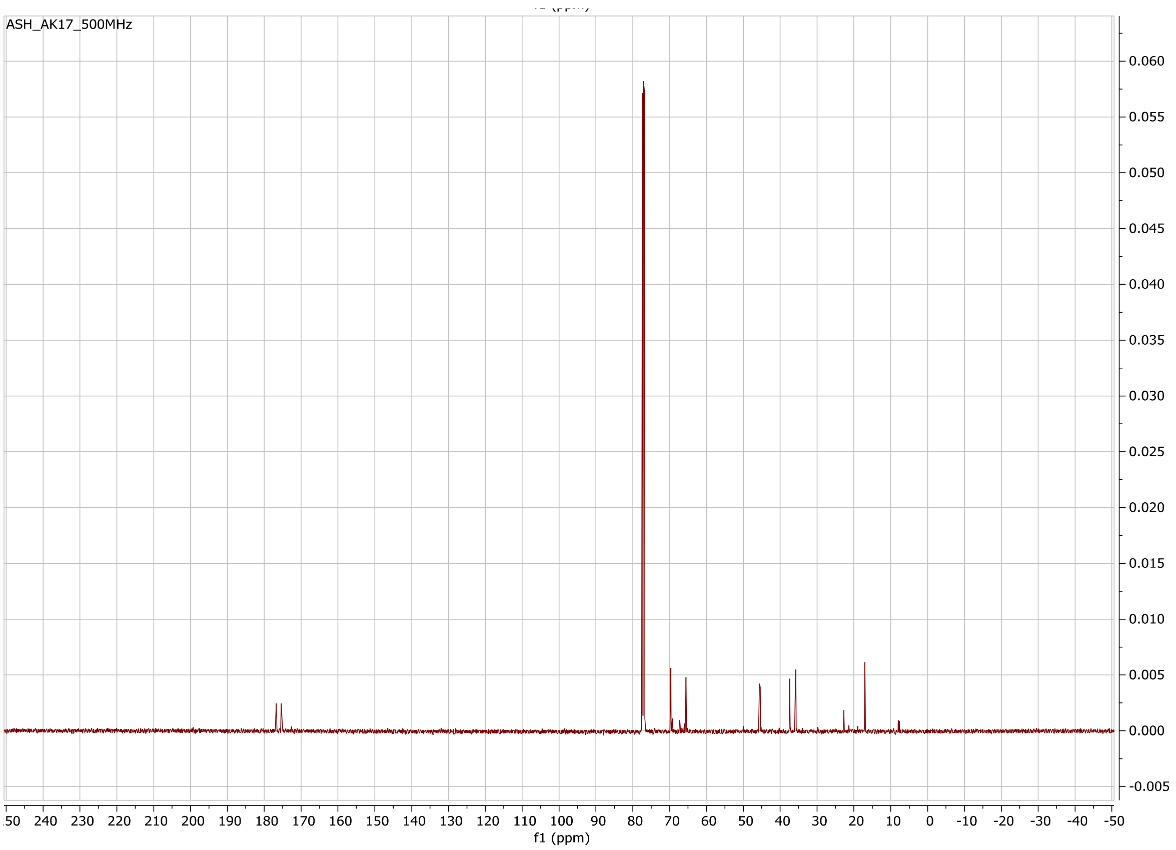

ASH_AK17_500MHz
0.060
0.055
0.050
0.045
0.040
0.035
0.030
0.025
0.020
0.015
0.010
0.005
0.000
-0.005
50 240 230 220 210 200 190 180 170 160 150 140 130 120 110 100 90 80 70 60 50 40 30 20 10 0 -10 -20 -30 -40 -50
f1 (ppm)
